# Acid Yellow 36, Methyl Red, and Methylene Blue adsorption using ammonia-modified red algae biochar: isotherm, kinetic, regeneration, and ANN studies

**DOI:** 10.1038/s41598-026-55117-4

**Published:** 2026-06-03

**Authors:** Mohamed A. Hassaan, Murat Yılmaz, Amany El Sikaily, Nehad A. Elmaghraby, Mohamed A. El-Nemr, Ahmed El Nemr

**Affiliations:** 1 Environment Division, National Institute of Oceanography and Fisheries (NIOF), Kayet Bey, Elanfoushy, Alexandria Egypt; 2 Bahçe Vocational School, Department of Chemistry and Chemical Processing Technologies, Osmaniye Korkut Ata University, Osmaniye, 80000 Turkey; 3 Department of Chemical Engineering, Faculty of Engineering, Minia University, Minia, 61519 Egypt; 4 The Higher Canal Institute of Engineering and Technology, Al Salam 1-Abu Bakr Al Siddiq Street, Suez, Egypt

**Keywords:** Adsorption kinetics, Ammonia-decorated biochar, Isotherm modeling, Red algae biochar, Azo dye removal, Chemistry, Environmental sciences, Materials science

## Abstract

This paper examined the removal of Acid Yellow 36 (AY36), Methyl Red (MR), and Methylene Blue (MB) dyes using a novel Ammonia-decorated Red Algae Biochar (RAB-A) synthesized from red algae (*Pterocladia capillacea*) via a reflux technique in the presence of 25% ammonium hydroxide (NH_4_OH). The physicochemical properties of the synthesized RAB-A, including its surface area, morphology, functional groups, elemental composition, and thermal stability, were comprehensively characterized through Brunauer–Emmett–Teller (BET) analysis, Fourier transform infrared (FTIR) spectroscopy, scanning electron microscopy (SEM) integrated with energy-dispersive X-ray (EDX) analysis, and thermogravimetric analysis (TGA). RAB-A demonstrated a low specific surface area (3.262 m^2^/g) and a monolayer adsorption capacity of 0.7495 cm^3^ (STP)/g. The adsorbent demonstrated an overall pore volume of 0.011 cm³/g, accompanied by an average pore diameter of 13.648 nm. Thermogravimetric analysis revealed an overall mass loss of 40.84% for RAB-A, demonstrating enhanced thermal stability relative to RAB, which showed a weight loss of 51.05%. FTIR analysis confirmed the presence of diverse functional moieties on the surface of RAB-A. Adsorption experiments targeting Acid Yellow 36 (AY36), Methyl Red (MR), and Methylene Blue (MB) were conducted in batch mode by independently adjusting the initial dye concentration (100–200 mg/L), contact time (5–180 min), solution pH (2–12), and adsorbent dosage (0.5–1.5 g/L). The adsorption equilibrium behavior was best described by the Langmuir isotherm, which indicated maximum uptake capacities of 222.22 mg/g for AY36, 192.31 mg/g for MR dye, and 833.33 mg/g for MB dye. Kinetic analyses revealed that the adsorption of all examined dyes was best described by a pseudo-second-order model, thereby demonstrating the high suitability of the synthesized RAB-A for the efficient elimination of dyes from aqueous solutions. Additionally, adsorption was predicted and adjusted utilizing artificial neural networks (ANN).

**Supplementary Information:**

The online version contains supplementary material available at 10.1038/s41598-026-55117-4.

## Introduction

In the 21st century, water contamination has become a major global issue, largely driven by accelerating industrial activity, growing urbanization, and the unsustainable use of natural resources—all of which place substantial pressure on ecological systems and human well-being^1^. Synthetic dyes constitute a significant class of environmental contaminants, owing to their widespread use across industries such as textiles, cosmetics, printing, food processing, paper manufacturing, and leather treatment^2,3^. Synthetic dyes play a vital role in the textile sector by providing color and enhancing the visual quality of fabrics. However, their extensive use also raises significant environmental and public health concerns. Globally, more than 10,000 synthetic dye varieties are manufactured, with annual production nearing 800,000 tons. A significant fraction of these dyes enters the environment through wastewater discharges, posing considerable ecological hazards^4,5^. Based on their dissociation behavior in aqueous media, synthetic dyes can be broadly categorized into three principal groups: anionic dyes, encompassing acid, direct, and reactive types; cationic dyes, predominantly associated with basic dyes; and nonionic dyes, which are primarily composed of disperse dye species^6^.

Metanil Yellow, commonly known as Acid Yellow 36 (AY36), belongs to the azo dye family and is widely applied in textile processes, particularly for coloring protein-based fibers like silk and wool. Beyond textile applications, AY36 is incorporated into products including soaps, shoe polishes, cleaning formulations, and various pigments. Although this dye is occasionally used in certain food products, including ice cream, sweets, and beverages, its use in food products is strictly forbidden owing to its well-documented carcinogenic properties^7,8^.

MR dye, an anionic azo dye, is widely used in various industrial processes and analytical laboratory applications. Nevertheless, its discharge into aquatic systems poses significant health risks, including irritation of the gastrointestinal tract, skin, and eyes^9–11^. The presence of benzene ring moieties in its molecular framework intensifies mutagenic effects under aerobic conditions while markedly hindering its biodegradation^12^. In light of these issues, the efficient removal of MR dye from polluted water is crucial before it is released into the environment.

Methylene Blue (MB), a cationic thiazine dye, is widely used in the textile, pharmaceutical, and printing industries. Nevertheless, its discharge into aquatic environments presents serious ecological risks. Even at low concentrations, MB can impair the photosynthetic efficiency of aquatic plants by reducing light penetration and interfering with chlorophyll activity, thereby disrupting oxygen generation and aquatic food webs^13,14^. In aquatic organisms, especially fish, exposure to Methylene Blue (MB) dye has been linked to gill irritation, behavioral changes, and histopathological effects, including oxidative stress and inhibition of enzymatic activity^15^. Prolonged exposure can also disrupt reproductive and developmental processes in aquatic organisms, potentially contributing to biodiversity decline^15^. Methylene Blue is regarded as safe at therapeutic doses below 2 mg/kg, whereas doses above 7 mg/kg have been linked to multiple adverse effects^16^. Given its toxicity and persistence, eliminating MB dye from wastewater prior to environmental release is essential.

Various methodologies have been established for the elimination of dyes, including the Fenton-like reaction^17^, photocatalytic degradation^18^, advanced oxidation processes^19^, membrane filtration^20,21^, biological treatments^22,23^, and adsorption^24,25^. Adsorption stands out among these methods owing to its straightforward operation, economic feasibility, high removal efficiency, versatility, and low production of secondary pollutants. It has been extensively utilized in the remediation of dye-contaminated actual wastewater^26–28^.

Activated carbon is widely recognized as a leading adsorbent for various dye-removal applications^29,30^. Despite its effectiveness, large-scale implementation is frequently constrained by elevated production expenses and challenges associated with material regeneration^31,32^. Consequently, numerous low-cost and readily available biomass residues—including spent coffee grounds^33^, sawdust^34^, pea pods^35^, date palm kernel^36^, watermelon peel^37^, cocoa pods^38^, rice husk^39^, palm petiole^40^, mandarin peel^24^, spinach waste^41^, and red algae^42^—have been explored as sustainable feedstocks for the synthesis of carbon-based adsorbents. These materials have shown promise for cost-effectively removing diverse contaminants from wastewater.

Recent research has increasingly explored the application of machine learning techniques for dye removal. For instance, Kumari et al.^43^ investigated the use of artificial neural networks (ANNs), adaptive neuro-fuzzy inference systems (ANFIS), and k-nearest neighbors (kNN) to eliminate crystal violet (CV) from wastewater. The study integrated a comprehensive statistical error analysis and reported a peak-removal efficiency of 97.46% under optimized experimental conditions. Although all models demonstrated reliable prediction of CV removal, the ANN achieved the highest accuracy among the tested approaches. In a separate study, Kumari et al.^44^ assessed the predictive efficacy of an ANN for adsorption-based treatment of simulated wastewater containing methylene blue (MB) and crystal violet (CV) using *Saccharum officinarum* L. They reported that, following a defined adsorption mechanism, maximum removal efficiencies of 98.3% for MB and 98.2% for CV were achieved^44,45^.

The efficiency of carbon-derived adsorbents in wastewater remediation is governed by the nature and abundance of surface functional moieties, which can be deliberately tailored through chemical modification strategies. In addition, approaches including nanoscale structural design, oxidative treatment, surface activation, and metal loading have been shown to further enhance adsorption efficiency^46^. The surface chemistry of carbon-based materials can be effectively engineered through treatments with acidic reagents (such as H₃PO₄, H₂SO₄, and HNO₃), alkaline compounds (e.g., KOH and NaClO), or oxidative agents (including NH₃·H₂O, (NH₄)₂S₂O₈, H₂O₂, KMnO₄, and NaClO), thereby increasing the abundance of functional groups on the carbon surface^47–49^.

In this paper, red algae (*Pterocladia capillacea*) were used as a sustainable raw material for the manufacture of highly porous carbon materials. The application of ammonia-modified, red algae–derived carbon as an adsorbent for the removal of aqueous pollutants represents a novel, environmentally benign remediation strategy. Red algae serve as a renewable, environmentally sustainable feedstock for carbon synthesis and can thrive in various aquatic environments, even those unsuitable for conventional use^42,50^. Algae-derived biochar that has been modified exhibits increased surface area and porosity, which play a key role in its ability to efficiently adsorb a wide range of contaminants, like dyes, heavy metals, and organic compounds from wastewater^51–53^. Moreover, the incorporation of nitrogen-based functional moieties substantially improves the surface chemical properties and adsorption performance of algae-derived biochar^54^. These results highlight the significant potential of biochar derived from chemically engineered red algae as an effective material for pollutant removal.

To the best of our knowledge, the adsorption efficacy of ammonium hydroxide–activated carbon synthesized under reflux conditions from red algae has not yet been reported for the removal of AY36, MR, and MB from aqueous systems. Although AY36 and MR are anionic dyes, whereas MB is a cationic dye, these pollutants differ markedly in their molecular structures, surface-active functional groups, and physicochemical properties. Examining the adsorption characteristics of these structurally diverse dyes enables a holistic assessment of RAB-A’s removal efficiency and selectivity for both anionic and cationic contaminants. This multi-dye evaluation strategy provides critical insight into the role of molecular and chemical heterogeneity in governing adsorption mechanisms, thereby confirming the adaptability and high performance of RAB-A as a promising adsorbent for industrial wastewater remediation.

This study examines the adsorption performance of RAB-A, an economical adsorbent derived from dried red algae, for the elimination of AY36, MR, and MB dyes from aqueous solutions. The influence of key operational variables, including initial dye concentration, solution pH, interaction time, and adsorbent loading, was systematically examined. Adsorption isotherm and kinetic models were used to elucidate the controlling mechanisms and assess the maximum adsorption capacity. Additionally, artificial neural networks (ANNs) were used to optimize adsorption conditions for all three dyes using RAB-A.

## Materials and methods

### Materials

#### Chemicals

Ammonium hydroxide solution (NH_4_OH, MW = 35 g/mol, 28% assay) was obtained from Sigma-Aldrich, USA. The standard stock solutions of Acid Yellow 36 Dye (C_18_H_14_N_3_NaO_3_S, MWt = 375.38 g, 98% assay), Methyl Red Dye (C_15_H_15_N_3_O_2_, MWt = 269.304 g, 98% assay) from Nice Chemicals, and Methylene blue (basic blue 9; C.I.52015, 99% assay), C_16_H_18_N_3_ClS.xH_2_O (Mwt = 319.85 g, 99% assay) from Honeywell Riedel-de Haën AG, SEELZE-HANNOVER, Germany. Sodium hydroxide (NaOH, minimum assay 96%) was procured from ADWIC, El Nasr Pharm. Chem. Co., Egypt, while hydrochloric acid (HCl, 30–34% assay) was procured from SD Fine-Chem Limited (SD FCL), Mumbai, India.

#### Sampling

Red algae collected from the Abu Qir region of Alexandria, Egypt, were extensively washed with deionized water to remove adhering epiphytes, microbial residues, sand particles, and other extraneous impurities. The red algae were identified by the Department of Taxonomy at our institute (NIOF). Following purification, the biomass was initially allowed to dry at ambient conditions for 1 h, then dried in an oven at 60 °C for 48 h, and finally ground to a fine particulate prior to biochar production.

All research and field studies involving red algae *Pterocladia capillacea* plants, including the collection of plant material, were conducted in accordance with relevant institutional, national, and international guidelines and legislation. The study also complied with the principles of the IUCN Policy Statement on Research Involving Species at Risk of Extinction and the Convention on International Trade in Endangered Species of Wild Fauna and Flora (CITES), where applicable.

#### Preparation of stock solution

**Fig. 1:**
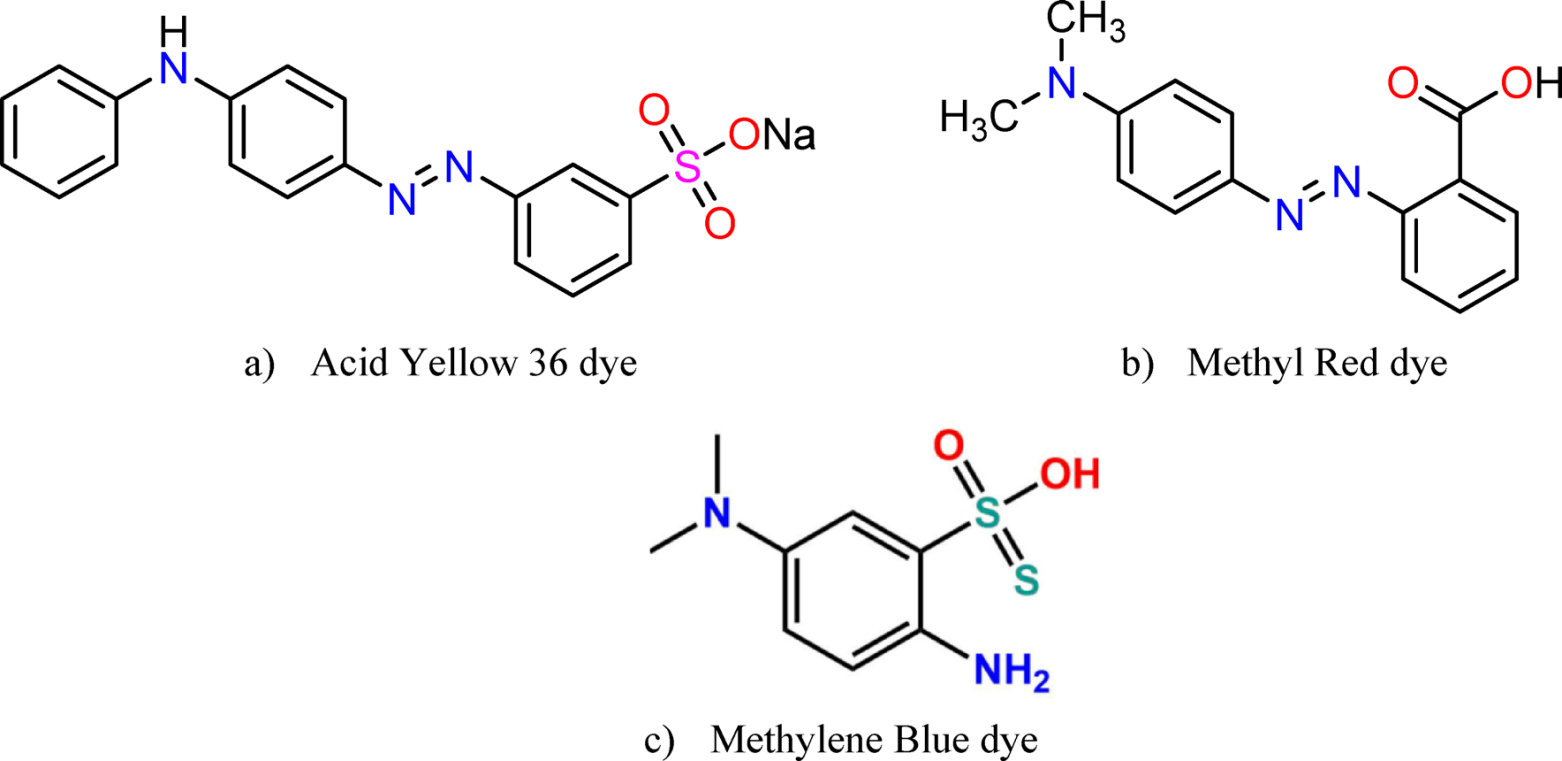
The chemical structure of (**a**) Acid Yellow 36 dye, (**b**) Methyl Red dye, and (**c**) Methylene Blue dye.

Individual stock solutions of Acid Yellow 36 (AY36), Methyl Red (MR), and Methylene Blue (MB) at 1000 mg/L were prepared by dissolving 1.0 g of each dye in 1000 mL of distilled water. Batch equilibrium studies were conducted by combining 100 mL of the dye solution with predetermined amounts of the synthesized adsorbent, then agitating on a mechanical shaker. Upon completion of the specified contact period, a 0.2 mL aliquot of the supernatant was withdrawn, and the residual dye concentration was determined using a UV–visible spectrophotometer at the respective maximum absorbance wavelength for each dye. The molecular structures of the investigated dyes are presented in Fig. 1.

### Preparation of red algae biochar (RAB)

A total of 300 g of red algae powder was introduced into a 5 L conical flask, and 1800 mL of concentrated H_2_SO_4_ (98%) was added gradually under continuous stirring. Thereafter, 200 mL of distilled water was gradually added, and the suspension was subjected to reflux at 300 °C for 4 h. Upon completion of the reflux process, the solid product was consecutively washed with 1 L of a standard NaHCO_3_ solution, followed by repeated rinsing with distilled water until neutral pH conditions were achieved, and finally rinsed with ethanol. The resulting biochar was subsequently dried in an oven at 105 °C to yield RAB (31% yield)^55^. For physical activation, the RA-derived biochar was suspended in water and treated using ultrasonic irradiation for 30 min. After sonication, the mixture was filtered, thoroughly rinsed with distilled water, and subsequently re-dried in an oven at 105 °C.

### Preparation of ammonia-modified red algae biochar (RAB-A)

The prepared RAB was further treated with ammonium hydroxide (NH_4_OH) for functionalization. Specifically, 20 g of RAB was refluxed in 100 mL of 25% NH_4_OH solution for 120 min. After cooling, the mixture was filtered, then washed with purified water and rinsed with ethanol. The functionalized biochar was then oven-dried at 105 °C for 48 h and designated RAB-A (yield: 25.5 g)^56^. Figure S1 provides a schematic illustration of the preparation of ammonia-modified red algae biochar (RAB-A) from red algae collected from the Abu Qir region of Alexandria, Egypt.

### Batch absorption procedure

The adsorption performance of ammonia-modified red algae biochar (RAB-A) for the elimination of AY36, MR, and MB dyes was investigated through batch experiments. The effects of contact time, initial dye concentration, adsorbent dosage, and solution pH on the adsorption capacity were systematically assessed under equilibrium conditions. Batch adsorption experiments were conducted using 100 mL Erlenmeyer flasks filled with dye solutions at initial concentrations of 100, 125, 150, 175, and 200 mg/L, while the RAB-A loading was adjusted between 0.5 and 1.5 g/L (0.5, 0.75, 1.0, 1.25, and 1.5 g/L), with all assays carried out at a constant temperature of 25 °C. The reaction mixtures were shaken in a temperature-controlled orbital shaker at 200 rpm until the adsorption equilibrium was reached. The residual dye concentrations were determined using a dual-beam UV-Vis spectrophotometer (PG Instruments, T80, UK) at their characteristic maximum absorption wavelengths: *λ*_max_ 434 nm for AY36 dye, *λ*_max_ 492 nm for MR dye, and *λ*_max_ 665 nm for MB dye.

To assess the effects of initial dye concentration and contact duration on adsorption efficiency, AY36, MR, and MB solutions were prepared at concentrations of 100–200 mg/L. The role of solution pH was examined by adjusting the initial pH values between 2 and 12. Equilibrium adsorption behavior was interpreted using multiple isotherm models, namely Langmuir (LIM), Freundlich (FIM), Temkin (TIM), Dubinin–Radushkevich (DRIM), Halsey (HIM), and Harkins–Jura (HJIM), to elucidate the underlying adsorption mechanism. Kinetic characteristics were analyzed by fitting the experimental data to the Pseudo-first-order (PFOM), Pseudo-second-order (PSOM), Elovich (EM), Intraparticle diffusion (IPDM), and Film diffusion (FDM) models. For each model, the best fit was assessed using the least-squares correlation coefficient (R^2^), along with the slope, intercept, and both calculated (*q*_*cal*_) and experimental (*q*_*exp*_) adsorption capacities. The equilibrium adsorption capacities (*q*_e_) were calculated using Eq. (1)^57^.

1 $$\:\:{q}_{e}=\frac{\left({C}_{0}-{C}_{e}\right)\:V}{m}$$

where the adsorption capacity (q_e_, mg/g) reflects the ability of the adsorbent to remove AY36, MR, and MB dyes from the aqueous phase at equilibrium conditions. In this expression, *C₀* (mg/L) denotes the initial dye concentration, whereas *C*_e_ (mg/L) represents the residual dye concentration in solution after the specified contact period. The removal efficiency of AY36, MR, and MB from water was determined according to Eq. (2).

2 $$\:Removal\:\%\:=\frac{\left({C}_{0}-{C}_{e}\right)\:}{{C}_{0}}\:\times\:100$$

### Characterization

The experiments were conducted using a JSOS-500 shaker, a T80 UV/Visible high-performance double-beam spectrophotometer, and a JENCO-6173 pH meter. The concentration of dyes was determined by UV–Vis spectrophotometry using a Jena SPEKOL 1300 instrument with 1 cm glass cuvettes.

The physicochemical characteristics of RAB-A were analyzed using a combination of techniques, including Fourier transform infrared (FTIR) spectroscopy, Brunauer–Emmett–Teller (BET) surface area analysis, X-ray diffraction (XRD), Scanning electron microscopy (SEM), Thermogravimetric analysis (TGA), and Energy-dispersive X-ray spectroscopy (EDX). X-ray diffraction (XRD) analysis was performed using a Bruker Meas Srv diffractometer (D2 PHASER, D2-208219/D2-2082019) operated at 30 kV and 10 mA with Cu Kα radiation (λ = 1.54 Å), scanning a 2θ interval from 5° to 80°. The morphology and particle size were examined with a scanning electron microscope (LEO 1450 VP). Samples were mounted on carbon tape and imaged in secondary electron mode at accelerating voltages of 5–20 kV, with magnifications ranging from 160× to 15,000×. The elemental composition of the samples was analyzed by EDX spectroscopy coupled with SEM.

The specific surface area of the RAB-A biochar was evaluated through nitrogen adsorption–desorption measurements. Prior to analysis, the samples were degassed at 300 °C for 3 h under a nitrogen atmosphere using a BELPREP-vac II unit (BEL Japan, Inc.). Pore structure measurements were then conducted using a BELSORP-mini 2 porosimeter (BEL Japan, Inc.) at 77 K with high-purity nitrogen (99.999%). The BET method was applied to ascertain the specific surface area (*S*_*BET*_), total pore volume (*V*_*T*_), and mean pore diameter (*D*_*P*_). The mesopore surface area (*S*_*me*_) and mesopore volume (*V*_*me*_) were derived using the Barrett–Joyner–Halenda (BJH) method, according to the BELSORP analysis software^58,59^. The average pore radius was calculated from Eq. (3).

3 $$\:r=\frac{{2V}_{T\:}(\mathrm{m}\mathrm{l}/\mathrm{g})}{{S}_{BET}({\mathrm{m}}^{2}/\mathrm{g})}\times\:1000$$

Where (*r*) represents the average pore radius (nm), (*V*_T_) denotes the total pore volume, and (*S*_BET_) signifies the surface area. Nitrogen adsorption–desorption isotherms were assessed at the boiling point of nitrogen (77 K). FTIR spectra were obtained in the wavenumber range of 400–4000 cm^–1^ using a VERTEX 70 FTIR spectrometer (V-100 model) fitted with a platinum attenuated total reflectance (ATR) accessory, enabling the identification of infrared-active functional groups on the carbon surface. Proximate analysis of RAB-A was carried out using a thermogravimetric analyzer (TGA, model SDT650). TGA was used to quantify the proportions of moisture, volatile matter, fixed carbon, and ash, with the total composition normalized to 100%. Thermal analysis was performed using a Thermal Analyzer over a temperature range of 50–1000 °C, with a heating rate of 5 °C per 60 s. X-ray photoelectron spectroscopy (XPS) was performed to determine the elemental composition using a Thermo Fisher Scientific K-Alpha spectrometer operated at a pass energy of 50 eV under an ultra-high vacuum with a base pressure of approximately 10⁻⁹ mbar.

### Artificial neural networks (ANN) modeling

ANNs use neurons to simulate biological brain networks and predict input-output relationships. The back-propagation neural network (BPNN) is among the most widely used neural network architectures. It consists of an input layer (IL) corresponding to the independent variables, one or more hidden layers (HNs), and an output layer (OL) that reflects the dependent variables. The removal performance of AY36, MR, and MB dyes by RAB-A was modeled using an ANN implemented in MATLAB R2015b and trained with the Levenberg–Marquardt (LM) optimization algorithm. The dataset was randomly divided into training (70%), validation (15%), and testing (15%) subsets. The optimal back-propagation neural network (BPNN) architecture comprised two hidden layers, each containing five neurons. In this model, the input parameters included the RAB-A dosage (g/L), contact time (min), and initial dye concentration (mg/L), while the corresponding output variable represented the removal efficiency of AY36, MR, and MB dyes^60–64^.

## Results and discussion

### Physico-chemical studies of RAB-A

**Fig. 2:**
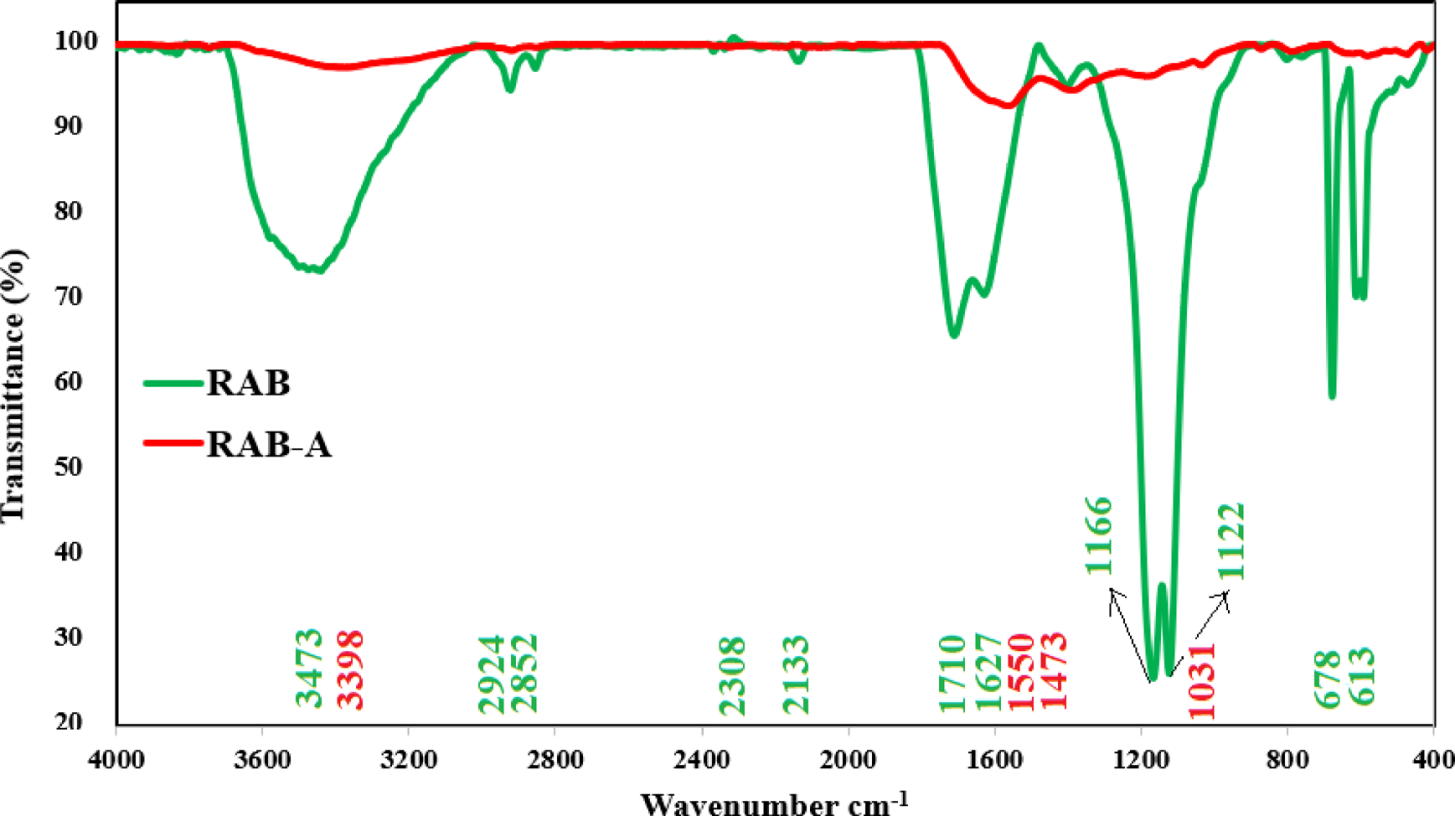
Comparison of FTIR profiles of RAB and RAB-A.

Fourier transform infrared (FTIR) spectroscopy was employed to identify the surface functional groups present on red algae–derived biochar (RAB) and its ammonia-modified counterpart (RAB-A). Comparison of the corresponding FTIR spectra (Fig. 2) revealed pronounced alterations in the nature and distribution of functional groups following activation. The broad absorption band observed at 3473 cm^–1^ in RAB is ascribed to the superposition of O–H stretching vibrations from hydroxyl groups and N–H stretching modes associated with amine or amide functionalities. In contrast, the slight shift and variation in band intensity to 3398 cm^–1^ in RAB-A suggest surface modification, likely involving the consumption, redistribution, or reorganization of hydroxyl and amine groups during the activation process. Asymmetric and symmetric C–H stretching of aliphatic –CH_2_/–CH_3_ groups from cellulose, hemicellulose, and lignin is represented by the peaks at 2924 and 2852 cm^–1^ in RAB; their reduction or smoothing in RAB-A indicates partial aliphatic chain breakdown and enhanced aromaticity upon activation. The absorption features observed in the 1627–1570 cm^–1^ region are attributed to aromatic C = C stretching vibrations and/or amide C = O groups, reflecting the presence of lignin-related structures and protein-derived constituents. Conversely, the band appearing near 1710 cm^–1^ in RAB is characteristic of C = O stretching associated with carboxylic acid, ester, or aldehyde functionalities. Following activation (RAB-A), deprotonation, decarboxylation, or condensation of carbonyl and aromatic groups are indicated by the significant decrease or disappearance of the 1710 cm^–1^ band and changes around 1627–1570 and 1473 cm^–1^, which are consistent with increased surface stabilization and potential generation of new acidic/basic sites. In RAB, C–O–C stretching of polysaccharides and C–O stretching of alcohols, phenols, and ethers are linked to strong bands in the 1200–1000 cm^–1^ area (e.g., 1166 and 1122 cm^–1^); the sharp peak at 1031 cm^–1^ further represents glycosidic C–O vibrations of cellulose/hemicellulose. Attenuation and mild shifting of these bands (1166, 1122, and 1031 cm^–1^) for RAB-A indicate breakage or rearrangement of C–O–C bonds and the formation of more condensed or cross-linked structures, which can create additional adsorption sites and generally improve surface heterogeneity. The absorption bands detected at 678 and 613 cm^–1^ in RAB are commonly attributed to backbone skeletal vibrations of polysaccharides or to out-of-plane C–H bending modes in substituted aromatic structures, thereby supporting the presence of an aromatic–polysaccharide framework. Overall, RAB-A’s much flatter spectrum (higher transmittance, lower band intensities) indicates that activation eliminated or altered several labile functional groups while preserving important O–H, C–O, and aromatic sites. These sites are usually involved in dye/metal binding through hydrogen bonding, electrostatic attraction, and π–π interactions^65,66^.

**Fig. 3:**
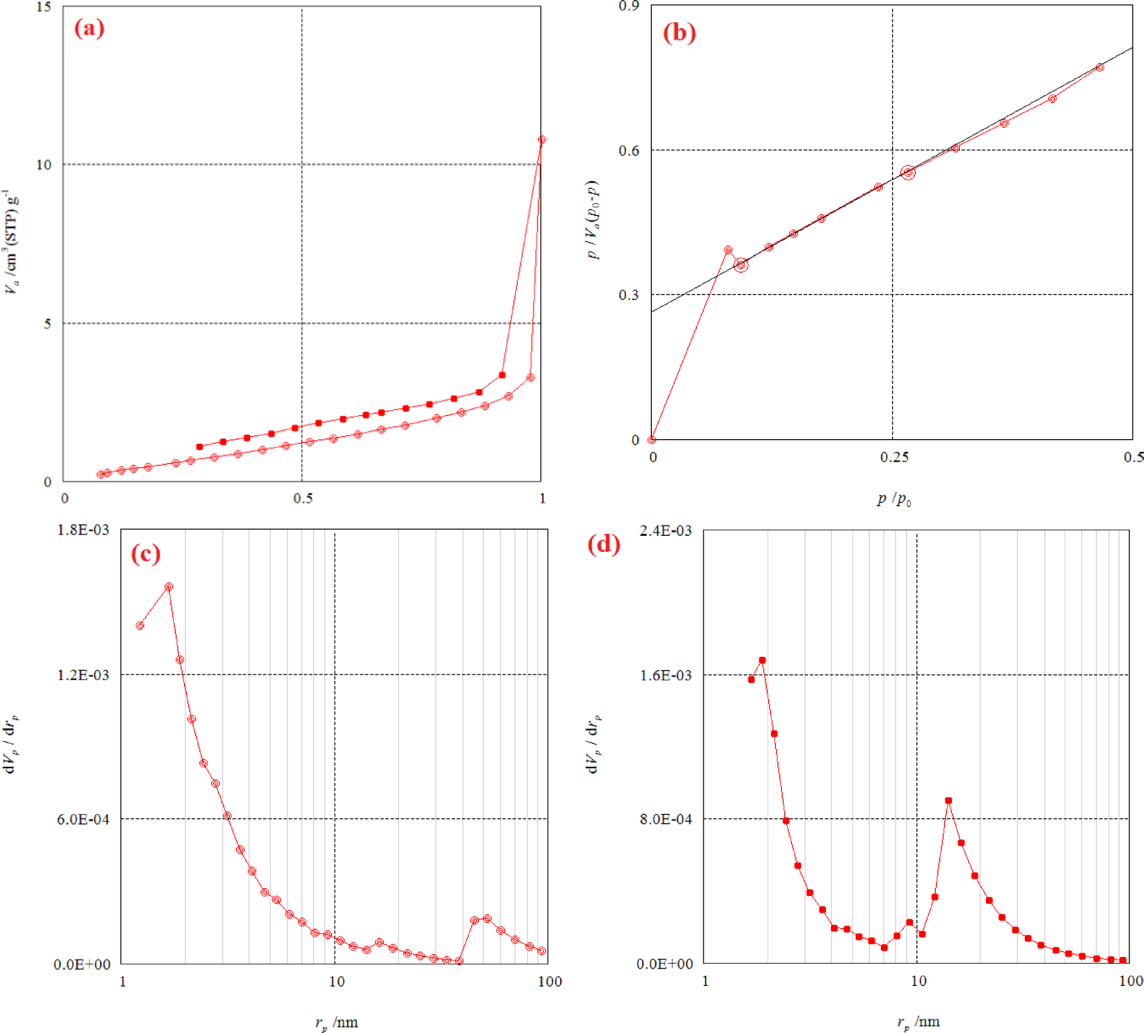
(**a**) Adsorption-desorption graph of RAB-A, (**b**) BET analysis graph of RAB-A, (**c**) BJH adsorption analysis graph of RAB-A, and (**d**) BJH desorption analysis graph of RAB-A.

The surface characteristics of RAB-A following NH₄OH modification were assessed through analysis of its N₂ adsorption–desorption isotherms. The specific surface area was calculated using the Brunauer–Emmett–Teller (BET) approach, whereas mesopore-related properties were evaluated through the Barrett–Joyner–Halenda (BJH) method. Key textural properties, such as the mean pore diameter, mesopore surface area, mesopore volume, total pore volume, monolayer capacity, and the peak of the mesopore size distribution, are presented in Table 1. Red algae-derived biochar-ammonia (RAB-A) is a mostly mesoporous material with a bimodal pore-size distribution focused in the 1–5 nm and 10–20 nm ranges, according to the nitrogen adsorption–desorption data. It also has a comparatively low specific surface area. The BET model’s applicability to N₂ at 77 K is confirmed by the BET plot’s linearity over a relative pressure range of roughly 0.05–0.30, and by the correlation line fitted through the red data points. The plot shows that the BET surface area (*a*_s, BET_) is 3.26 m^2^/g, the monolayer capacity *V*_m_ is 0.7354 cm^3^(STP)/g, and the BET constant (*C*) is roughly 5.15, showing significant adsorbate–adsorbent interactions typical of low–surface area carbons with little microporosity. The adsorption–desorption isotherm exhibits a prominent hysteresis loop between the adsorption and desorption branches as well as a progressive increase in adsorbed volume at low to intermediate relative pressures, followed by a rapid uptake at p/p_0_ ≈ 1. This behavior is consistent with a type IV isotherm with H_3_/H_4_-type hysteresis, which is typical of mesoporous solids with aggregates of plate-like particles or slit-like pores. This suggests that the RAB-A biochar has interparticle voids and mesopores produced by the carbonized algal framework. Two primary pore populations are visible in the BJH desorption branch: a broad contribution that extends into the 10–20 nm range, consistent with coexisting narrow mesopores and wider mesopores/macropore onsets, and a conspicuous peak about 1.9 nm (reported rp, peak ≈ 1.88). With a major peak at about 1.7 nm (reported rp, peak ≈ 1.66) and a tail toward greater radii, the BJH adsorption branch offers a comparable but slightly skewed distribution that supports the existence of ink-bottle or restricted pores where desorption is delayed relative to adsorption. In addition to the low BET surface area, the total pore volume near *p*/*p*_0_ = 0.99 is roughly 0.011–0.015 cm^3^/g, indicating that RAB-A is a low-porosity biochar compared to highly activated carbons, yet it still offers accessible mesoporous volume. The BET report’s mean pore diameter is approximately 13.909 nm, which is more than the BJH peak positions. This indicates that wider mesopores and interparticle voids, rather than only the dominant small mesopores, have a significant impact on the average. RAB-A may have a modest capacity for small gas molecules in comparison to high-surface area activated carbons, but its mesoporous structure with limited microporosity suggests that it will be more appropriate for adsorption of relatively large molecules (such as dyes, organic contaminants, and metal complexes) where steric access to pores larger than 2 nm is advantageous (Fig. 3). When interpreting equilibrium capacities and regeneration behavior in water-treatment experiments using this biochar, it is crucial to note that the moderate BET constant (*C*) value and the hysteresis pattern also suggest that adsorption will be more controlled by surface functional groups and pore connectivity than by strong physisorption in ultra-micropores^37,67,68^.

**Table 1:** Analysis of the surface area of RAB-A adsorbent.

Method	Parameter	Value	Unit
BET analysis	Monolayer volume (*V*_m_)	0.7354	cm^3^ (STP)/g
	Energy constant (the first layer) (*C*)	5.1532	
	Mean pore diameter	13.909	nm
	BET specific surface area (*a*_s, BET_)	3.262	m^2^/g
	Total pore volume	0.01130	cm^3^/g
BJH analysis Adsorption	Pore volume (*V*_p_)	0.01184	cm^3^/g
	The pore specific surface area (*a*_p_)	4.0021	m^2^/g
	Micropore radius (cylindrical shape) (*r*_p, peak_ (Area))	1.66	nm
BJH analysis Desorption	Pore volume (*V*_p_)	0.04471	cm^3^/g
	The pore specific surface area (*a*_p_)	3.0799	m^2^/g
	Micropore radius (cylindrical shape) (*r*_p, peak_ (Area))	1.88	nm

**Fig. 4:**
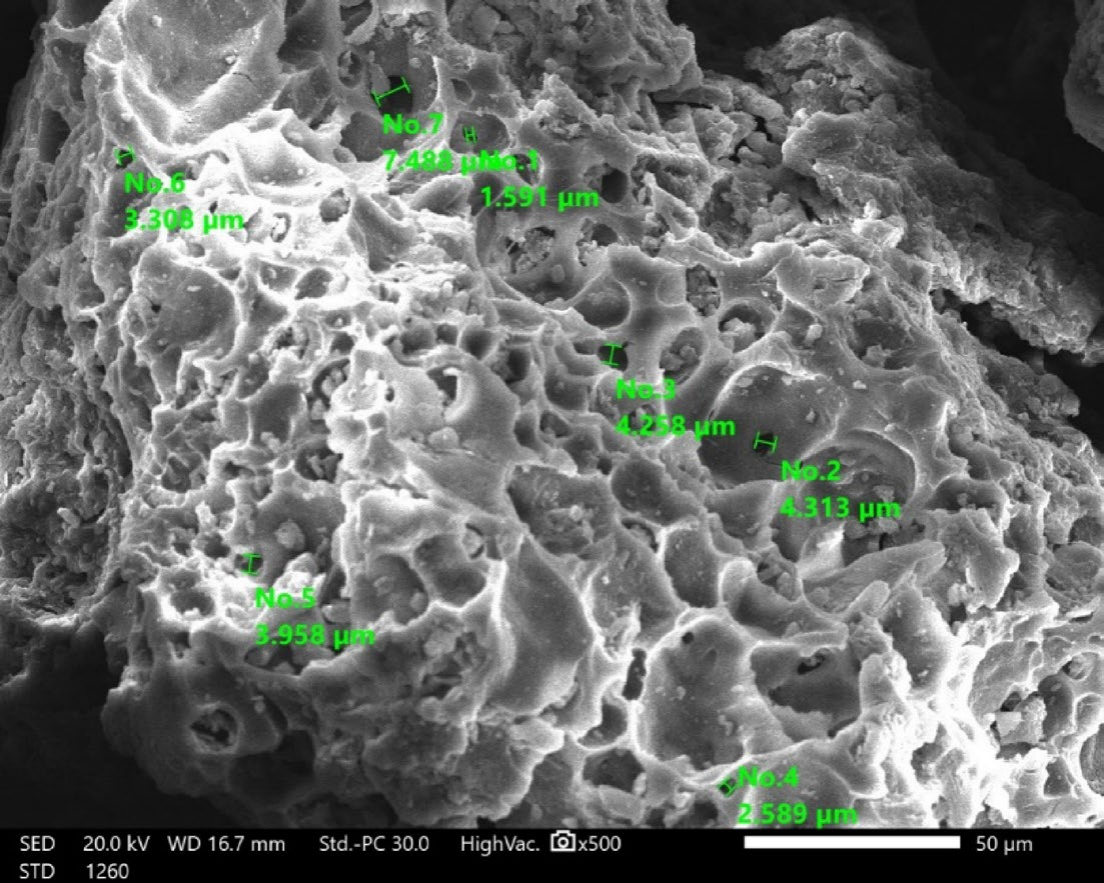
Surface morphology of RAB-A examined using SEM (20.0 kV, ×500).

Figure 4 displays the SEM images of RAB-A, demonstrating a clean morphology with no discernible impurities. Notably, the porous architecture of RAB-A remained intact even after the rigorous NH₄OH modification process^66^. RAB-A’s SEM micrograph shows a highly porous, sponge-like surface morphology with irregular cavities scattered throughout the matrix and a linked network of macropores (Fig. 4). With many measured diameters (e.g., around 1.5–4.5 μm) labeled on the image, the pores show a broad size variation in the micrometer range, showing significant textural variability over the particle surface. This rough, curved topology, with plenty of pits, channels, and protrusions, suggests a highly accessible surface area. This is good for mass transport and offers many potential sites for adsorption and surface interactions in water treatment applications. The presence of both blind cavities and open pores suggests that RAB-A probably has a complex internal structure, with smaller constrictions improving solute retention within the matrix and linked gaps facilitating fluid penetration. RAB-A is a highly structured, micro to macroporous material with an uneven surface and pore architecture that is ideally suited for usage as an active sorbent or catalytic support in environmental remediation systems, according to the SEM picture.

**Fig. 5:**
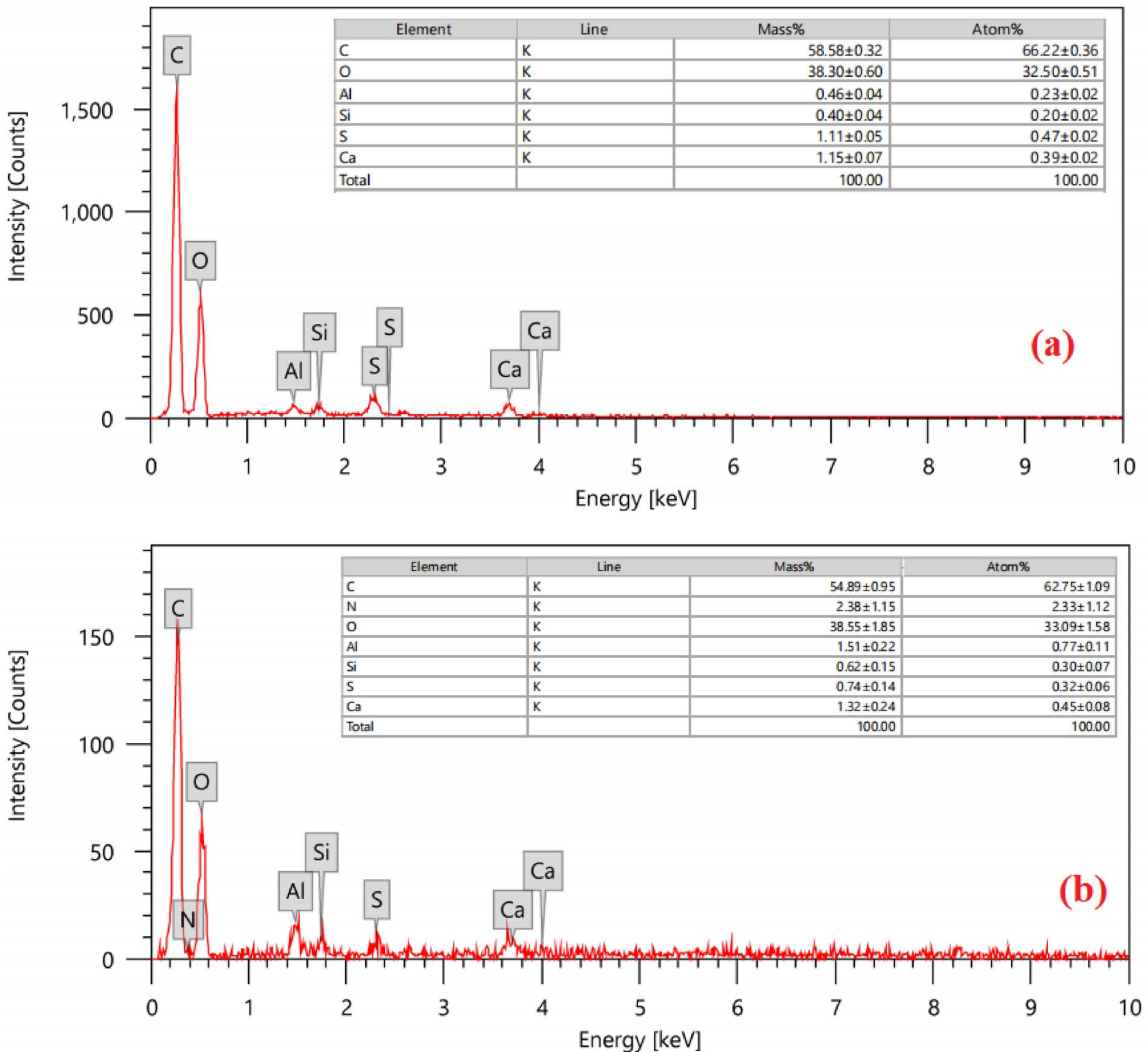
EDX analysis of (**a**) RAB and (**b**) RAB-A.

Energy-dispersive X-ray (EDX) analysis was used to characterize the elemental composition of the RAB and RAB-A adsorbent materials. As illustrated in Fig. 5a, carbon was the predominant element, comprising 58.58 ± 0.32% of the RAB. Oxygen (38.30 ± 0.60%), aluminum (0.46 ± 0.04%), silicon (0.40 ± 0.04%), sulfur (1.11 ± 0.05%), and calcium (1.15 ± 0.07%) were identified in significant quantities. Figure 5b illustrates that carbon was the predominant element, comprising 54.89 ± 0.95% of the RAB-A. Notable quantities of oxygen (38.55 ± 1.85%), nitrogen (2.38 ± 1.15), aluminum (1.51 ± 0.22%), silicon (0.62 ± 0.15%), sulfur (0.74 ± 0.14%), and calcium (1.32 ± 0.24%) were also identified^67^. The absence of nitrogen in RAB but its presence in RAB-A indicates that the NH_4_OH modification was successful.

**Fig. 6:**
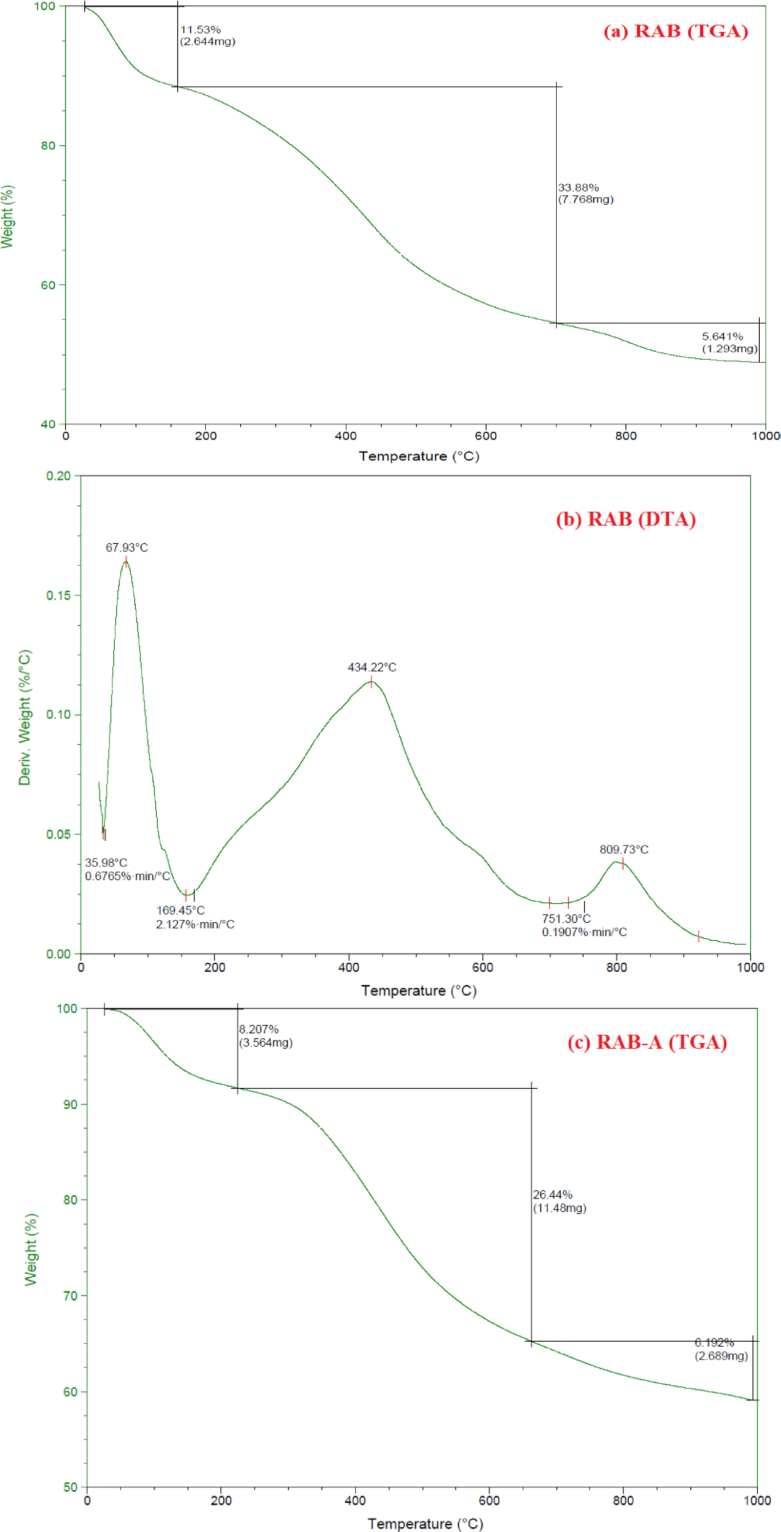

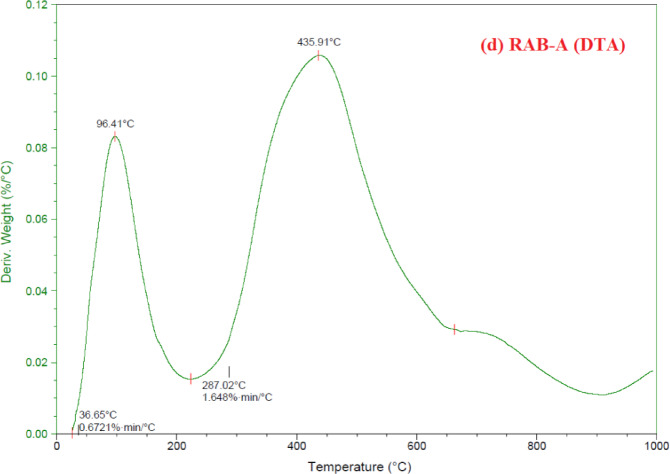
TGA curves of (**a**) RAB, and (**c**) RAB-A; DTA curves of (**b**) RAB, and (**d**) RAB-A.

TGA analysis was performed to evaluate the influence of modification on the thermal degradation profile and stability of RAB and RAB-A (Fig. 6a–d). The materials were heated from 50 to 1000 °C under a N_2_ atmosphere. The resulting TGA, DTA, and DSC profiles are shown in Fig. 6. An initial mass loss below 200 °C was observed for both materials and is attributed to the removal of physically adsorbed moisture. At temperatures above 200 °C, additional weight loss occurred due to the breakdown of oxygenated acidic functional groups. RAB showed three distinct decomposition stages within the temperature intervals of 25–160, 160–700, and 700–990 °C, resulting in a total mass loss of 51.05% (Fig. 6a). In contrast, RAB-A exhibited three degradation regions between 25 and 225, 225–665, and 665–990 °C, with a lower cumulative mass loss of 40.84%, reflecting enhanced thermal stability relative to RAB (Fig. 6c). The stabilization of the TGA curves was observed at temperatures above 435.91 °C for RAB-A and 67.93 °C for RAB, which can be attributed to the progressive degradation of the carbonaceous matrix (Fig. 6b,d)^67^.

The DTA curves corresponding to RAB and RAB-A are depicted in Fig. 6b,d, respectively. In the case of RAB, three well-defined thermal events were detected at 67.93, 434.22, and 809.73 °C (Fig. 6b), whereas the DTA profile of RAB-A displayed two prominent peaks located at 96.41 and 435.91 °C (Fig. 6d). The DTA profiles indicate that the conversion of RAB to RAB-A proceeded through two principal dehydration-related degradation stages. Importantly, after modification with 25% NH₄OH, the characteristic degradation signals of RAB-A shifted toward higher temperatures, demonstrating that ammonia treatment markedly improved the material’s thermal stability^67^. Both RAB and RAB-A’s DTA profiles reveal multi-step thermal processes, including the removal of physically adsorbed water, dehydroxylation/organic burn-off, and high-temperature carbonate decomposition. At low temperatures, RAB-A exhibits somewhat better thermal stability. The temperature differences between the sample and the inert reference are measured by differential thermal analysis, and each peak corresponds to an endothermic or exothermic event, such as a phase transition, combustion, dehydration, or dehydroxylation. Endothermic peaks below 200 °C are usually attributed to the loss of physically adsorbed and interlayer water, peaks between 400 and 450 °C to the breakdown of hydroxides such as Ca(OH)_2_, and peaks between 700 and 800 °C to the breakdown of carbonates such as CaCO_3_. Due to the desorption of physically adsorbed moisture and loosely bound water from pores and surface sites, RAB displays a prominent low-temperature peak with a maximum near 68 °C (beginning around 36 °C), followed by a minor feature near 160 °C. In comparison to RAB, RAB-A exhibits its first major peak displaced to ~ 96 °C (onset ~ 37 °C), indicating slightly greater water binding or less free moisture. This is consistent with partial structural alteration or a decrease in hydrophilic areas following the treatment. The broad peak around 434 °C for RAB represents an endothermic process linked to the dehydroxylation of structural hydroxyl groups and/or the breakdown of metal hydroxides (like Ca(OH)_2_) and organic residues that are still present in the raw material. The activation step reorganizes hydroxylated phases and organic species, potentially promoting a more homogeneous dehydroxylation pathway. The minor intermediate feature observed in RAB vanishes (about 287 °C shows exclusively in RAB-A), while RAB-A exhibits a similar dominant peak at ~ 436 °C. In RAB, carbonate decomposition—such as CaCO_3_ → CaO + CO_2_, which often appears between 700 and 900 °C in DTA/TG curves—is characterized by a distinct high temperature peak about 809.73 °C and a slight shoulder near 751 °C. Although it is less intense, RAB A still exhibits a broad high-temperature endotherm, which would be compatible with previous decarbonation or phase conversion. This suggests a decreased carbonate concentration or partial transformation of carbonate phases during activation. Together, these characteristics imply that the activation process that transforms RAB into RAB-A eliminates a portion of the weakly bound water and carbonate fractions and rearranges hydroxylated and organic species, resulting in a more thermally stable, potentially more porous structure that is advantageous for high-temperature applications such as adsorption or catalysis (Fig. 6).

**Fig. 7:**
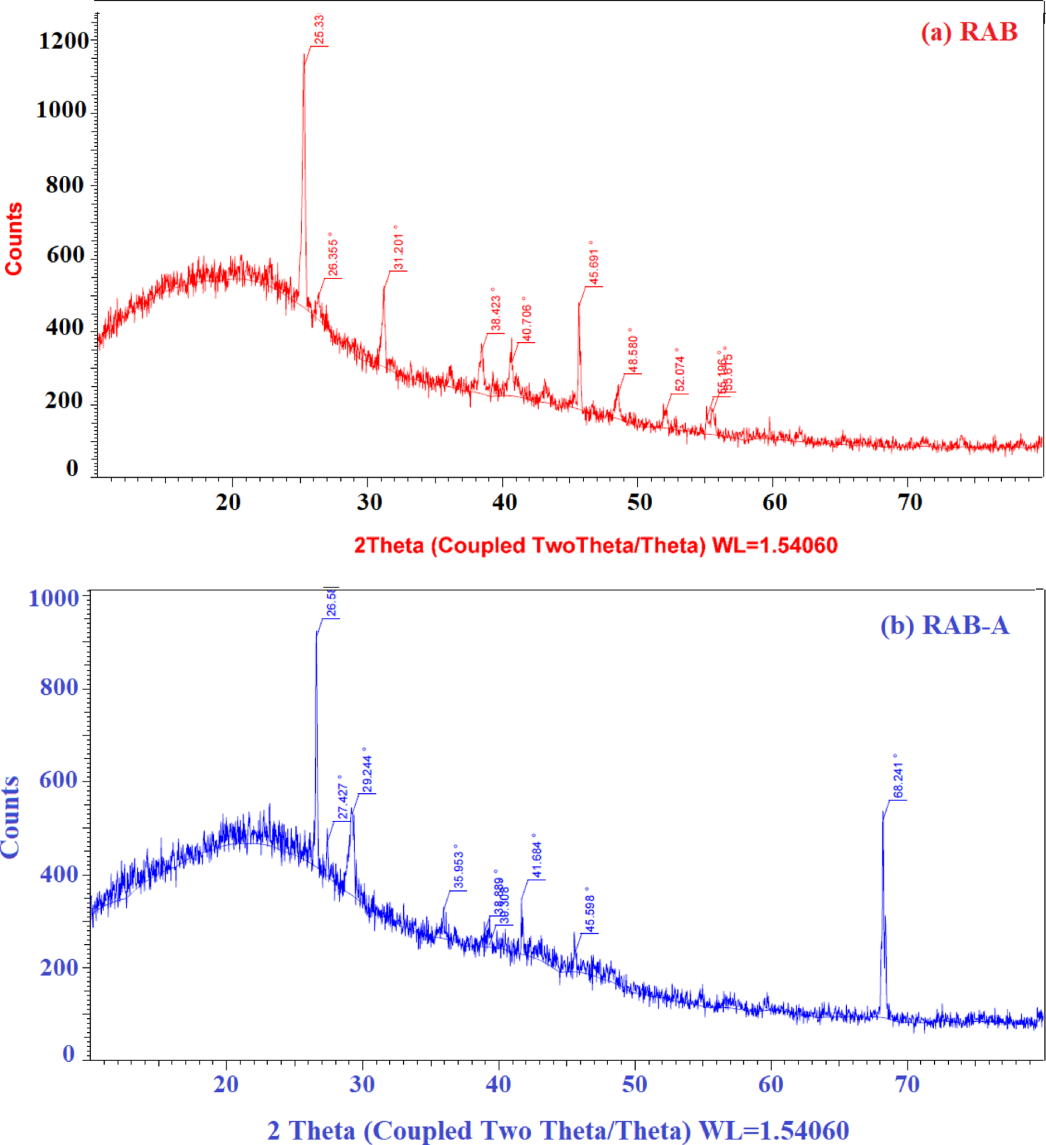
XRD analysis of (**a**) RAB, and (**b**) RAB-A.

The XRD patterns of RAB and RAB-A show that while the phase composition of both materials is generally comparable, there are differences in peak intensities and crystallinity, which may imply minor structural or compositional changes during the conversion of RAB to RAB-A. The XRD patterns of RAB-A shown in Fig. 7 reveal a predominantly amorphous carbon framework with disordered aromatic stacking. A broad reflection centered at 2θ ≈ 24.9° is attributed to the C (002) plane, whereas a weaker diffraction signal is observed at 2θ = 42.54°. Furthermore, the presence of a weak diffraction peak at approximately 2*θ* ≈ 15.4°, corresponding to the (101) reflection, indicates the persistence of crystalline cellulose. This peak may additionally be attributed to minor inorganic constituents, such as quartz and albite, which is a typical member of the plagioclase feldspar group^50,67,69,70^. A partly crystalline aluminosilicate-type matrix with both ordered and amorphous domains is characterized by a large hump at low angles superimposed with multiple sharp reflections in the diffraction profiles of RAB and RAB-A. The primary peak positions in both patterns coincide (within experimental error), suggesting that RAB-A maintains the parent RAB material’s framework structure and that no new crystalline phase emerges upon alteration. The presence of a sizable amorphous fraction is confirmed by the sharp reflections in both diffractograms, which are situated on a comparatively high background. Nonetheless, there are differences in the full width at half maximum (FWHM) and relative strength of the major peaks between RAB and RAB-A, indicating variations in microstrain and/or crystallite size related to the process employed to produce RAB-A. A higher degree of preferred orientation or more clearly defined stacking of the major crystallographic planes in the original material is indicated by the fact that RAB exhibits a more intense reflection at low 2*θ* (around the first strong peak) than RAB-A^67–70^. On the other hand, RAB-A exhibits several medium-angle peaks that are comparatively more prominent, indicating a partial redistribution of electron density or cation sites within the structure instead of a full phase shift. Both patterns show a wide halo extending roughly from the mid-teens to the mid-thirties in 2*θ*, suggestive of glassy or disordered aluminosilicate phases, which are usually the result of incomplete crystallization or thermal activation. A little increase in structural ordering or elimination of amorphous impurities during the modification stage is suggested by the somewhat lower background in RAB-A compared to RAB in this area. From a functional standpoint, the coexistence of an amorphous matrix (which provides additional defect sites and surface heterogeneity) and crystalline domains (which offer specific lattice sites and well-defined channels) is beneficial for adsorption or catalytic applications anticipated for these materials. The modification may produce more structurally distorted or accessible sites, which can promote ion exchange, surface complexation, or active site exposure without compromising the overall structural stability inherited from RAB, according to the slight decrease in crystallinity and the relative enhancement of some secondary peaks in RAB-A^50,67,69,70^.

**Fig. 8:**
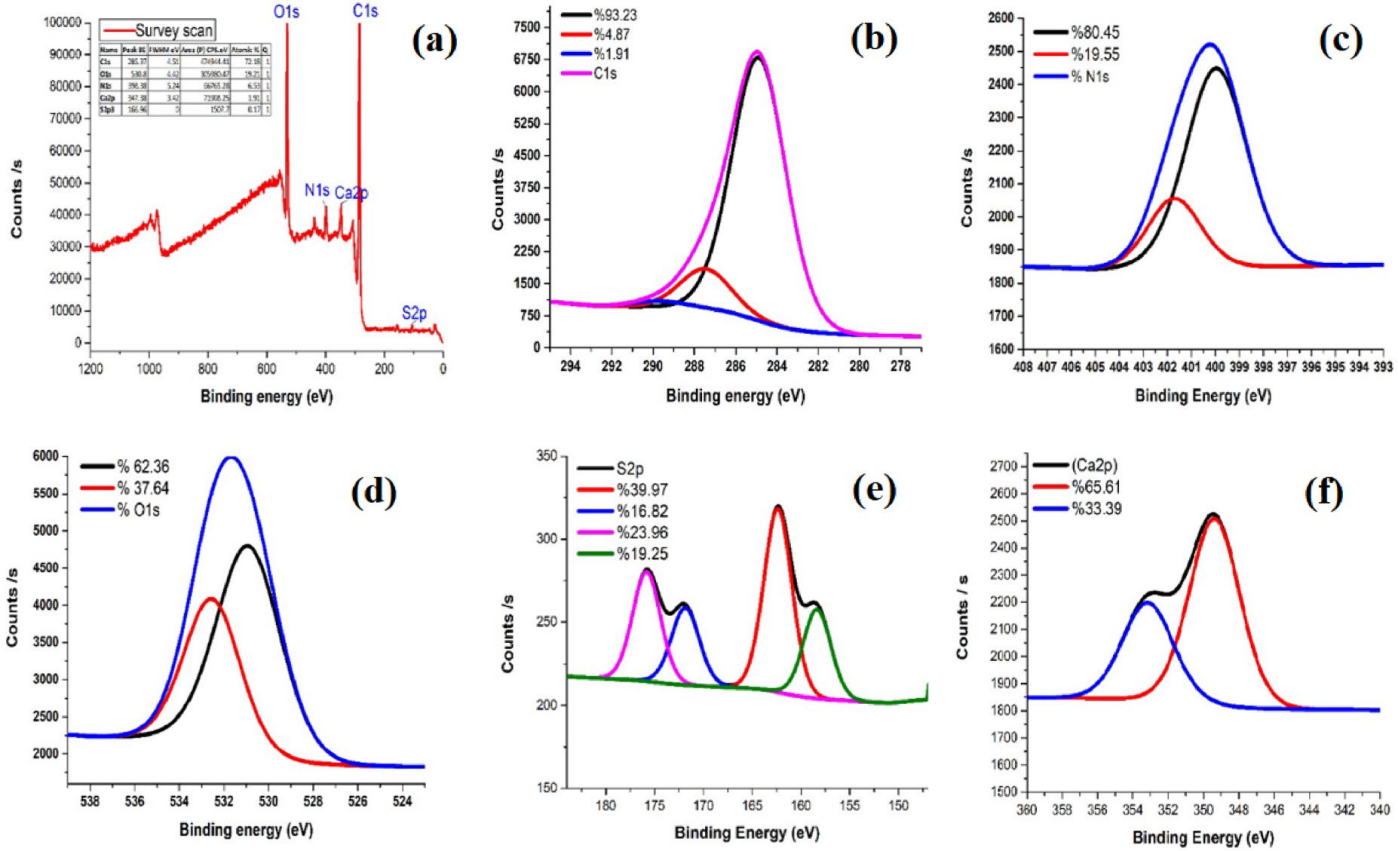
XPS core level spectra of the whole RAB-A spectrum.

The surface chemical functionalities of the activated carbon samples were qualitatively characterized using X-ray photoelectron spectroscopy (XPS). Figure 8a presents the wide-scan XPS spectra of the precursor and RAB-A samples. The spectra of RAB-A exhibit distinct signals corresponding to C 1s, O 1s, N 1s, Ca 2p, and S 2p, confirming the successful incorporation and retention of nitrogen species on the RAB-A surface. The peaks located in 285.37, 398.38, 530.8, 347.38, and 166.96 eV correspond to C1s, O1s, N1s, Ca2p, and S2p, respectively. In Fig. 8b, the C 1s spectrum was deconvoluted into three components at 284.98 eV (C–C/C = C), 288.05 eV (C = O, including amide-like species), and 289.68 eV (O–C = O), indicating a predominantly graphitic carbon structure with minor oxygen-containing functional groups after ammonia treatment^71,72^. Deconvolution of the N 1s XPS spectrum of RAB-A revealed the presence of two distinct nitrogen functionalities, as illustrated in Fig. 8c. The fitted peaks located at binding energies of 399.96 eV and 401.7 eV are assigned to pyridinic nitrogen and graphitic nitrogen species, respectively^71–73^. As illustrated in Fig. 8d, the O1s XPS spectrum of RAB-A is deconvoluted into two distinct components located at binding energies of 530.9 eV and 532.53 eV, corresponding to carbonyl/quinone (C = O) and hydroxyl/ether (C–O) functionalities, respectively^72^. S2p XPS spectrum shows four peaks at 158.35, 162.36, 171.85, and 175.82 eV, corresponding to reduced sulfur species (metal–S/thiolate), thiophene-like sulfur (C–S–C), and oxidized sulfur species such as sulfonate and sulfate, respectively (Fig. 8e). Ca2p XPS spectrum exhibits two peaks at 349.37 eV (Ca 2p_3/2_) and 353.16 eV (Ca 2p_1/2_), indicating the presence of Ca^2+^ species, such as calcium carbonate or calcium oxide, naturally inherited from the red algae biomass (Fig. 8f).

### Adsorption of AY36 dye and MR dye on GASC

#### pH_PZC_ and Effect of pH

**Fig. 9:**
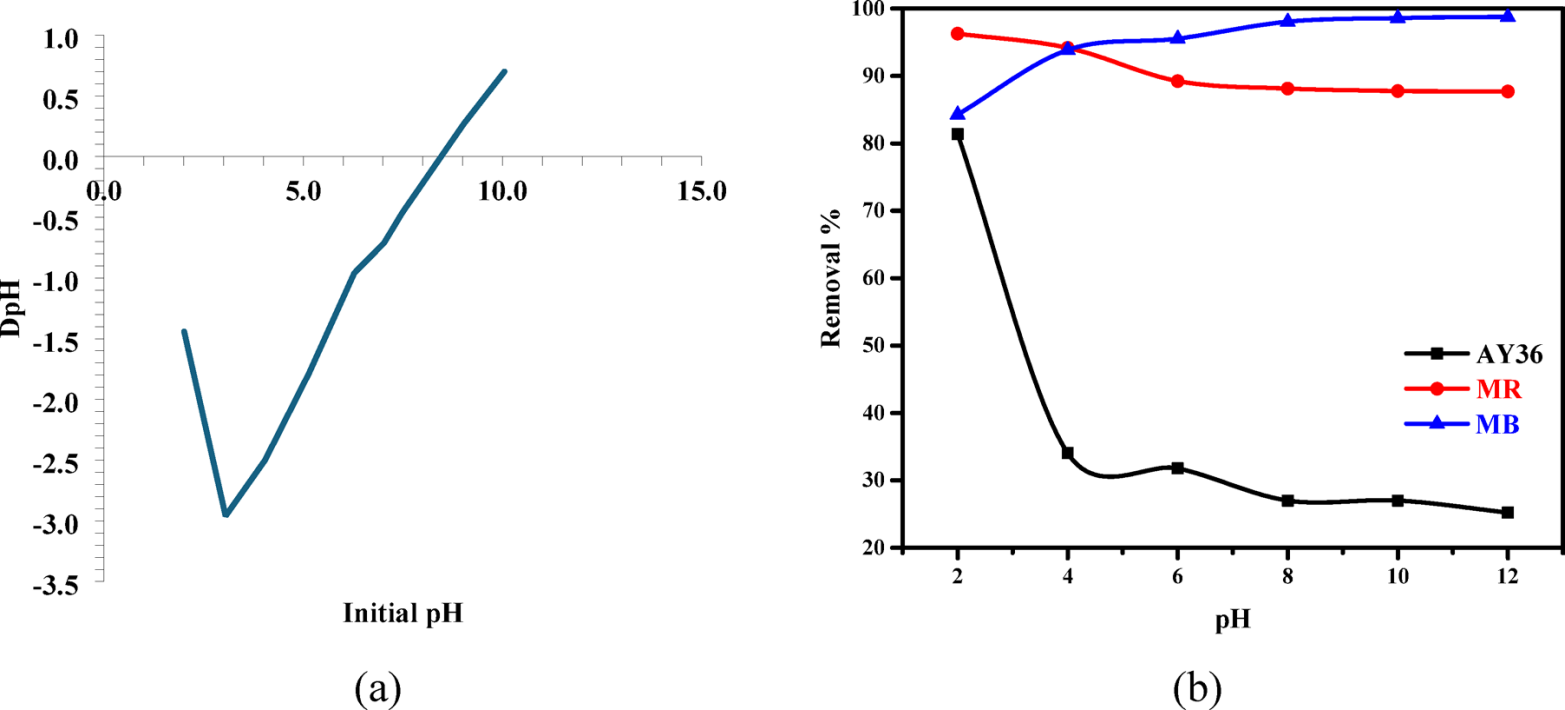
(**a**) pH_PZC_, (**b**) The pH-dependent removal of AY36, MR, and MB dyes using RAB-A (*C₀* = 100 mg/L, adsorbent 1.0 g/L, time = 180 min, T = 25 °C).

Figure 9a shows the point of zero charge (pH_PZC_) and how ΔpH changes based on the initial solution pH. This is how we find the point of zero charge for RAB-A. The pH_PZC_ was estimated to be around 8.8, which is the same as the point where ΔpH = 0. This means that RAB-A’s surface is positively charged at pH values below 8.8 because protons are added to its functional groups. At pH levels above 8.8, however, it becomes negatively charged because protons are removed. The relatively high pH_PZC_ value indicates that RAB-A has a positive surface charge over a wide pH range, from acidic to near-neutral. This is effective for adsorbing anionic dyes. The surface charge behavior aligns with the pH-dependent adsorption findings, indicating that AY36 and MR exhibited optimal removal in acidic environments, whereas MB adsorption intensified in alkaline settings. The pH_ZPC_ analysis shows that electrostatic interactions are crucial in determining how dyes bind to RAB-A. The pH_PZC_ analysis shows that electrostatic interactions are crucial in determining how dyes bind to RAB-A.

Textile effluents exhibit wide pH fluctuations, which strongly influence adsorption behavior. The pH of the aqueous medium governs adsorption by modulating the protonation states of surface functional groups on biochar, including amino, hydroxyl, and carboxyl moieties. In this study, the removal performance of AY36, MR, and MB dyes was evaluated at 25 °C under fixed experimental conditions, employing an initial dye concentration of 100 mg/L and an RAB-A adsorbent loading of 1 g/L. The experiments were carried out for 180 min over a pH range of 2 to 12. As shown in Fig. 9b, the highest elimination efficiencies for AY36 and MR were achieved under strongly acidic conditions (pH 2), whereas MB exhibited maximum adsorption at alkaline pH (pH 12), with corresponding removal efficiencies of 81.4%, 96.27%, and 98.77%, respectively. A progressive decline in adsorption efficiency with increasing pH was observed for AY36 and MR, whereas MB showed a continuous increase in removal efficiency with increasing pH. These findings are consistent with previous reports emphasizing the decisive role of solution pH in the adsorption performance of azo dyes. Previous studies have consistently reported a pronounced dependence of azo dye adsorption on solution pH. Song et al.^74^ reported that the uptake capacity of Sunset Yellow declined markedly when the pH was raised from 2 to 4. Similarly, Khaled et al.^75^ documented a drastic loss in adsorption efficiency for Direct Yellow 12, with removal percentages dropping from 98.1% to 11.1% as the pH increased from 1.5 to 11.1. In another related study, Eleryan et al.^25^ investigated the pH-dependent adsorption behavior of Acid Yellow 11 onto Mandarin Biochar-TETA (MBT) derived from *Citrus reticulata* peels and found that the removal efficiency decreased substantially, from 66.5% at pH 1.5 to only 1.3% at pH 12. Conversely, Shoaib et al.^76^ reported an opposite trend in MB dye adsorption onto nitrogen-doped *Ulva lactuca* biochar, with removal efficiency increasing markedly from 42% to 95% as the pH rose from 2 to 12. In line with the aforementioned findings, the adsorption experiments in this work revealed that a solution pH of 2 provides the most favorable conditions for the uptake of AY36 and MR dyes by RAB-A, while the highest removal efficiency for MB was obtained under strongly alkaline conditions (pH 12).

At elevated pH values, an increased abundance of hydroxide ions (OH⁻) hinders the adsorption of anionic dyes, including AY36 and MR, by competing for active sites on the RAB-A surface, thereby reducing overall adsorption performance. Due to their elevated mobility and abundance, OH⁻ ions are preferentially adsorbed, further limiting dye uptake. Conversely, at low pH, protonation of the RAB-A surface increases the density of positively charged adsorption sites, thereby intensifying electrostatic interactions with anionic dye species. This strengthened attraction explains the markedly improved removal efficiencies of AY36 and MR obtained under highly acidic conditions (pH 2) (Fig. 9b). The hydrophobic characteristics of ammonia-modified biochar also contribute to this behavior. Upon contact with water, hydrogen ions bind to the carbon surface, imparting a positive charge that facilitates electrostatic adsorption of anionic dyes. In the case of the cationic MB dye, adsorption is unfavorable under strongly acidic conditions (e.g., pH 2) because electrostatic repulsion arises between the positively charged RAB-A surface and the dye ions. When the solution pH exceeds the isoelectric point (pH 12 in this work), deprotonation of surface acidic functionalities imparts a net negative charge to RAB-A, thereby strengthening electrostatic attraction toward MB and enhancing adsorption capacity^76^.

The pH of the solution has a big effect on how well RAB-A adsorbs dye. This is due to the adsorbent’s surface charge and the ionic nature of the dye molecules. The point of zero charge (pH_PZC_) for RAB-A was about 8.8. This means that the adsorbent surface remains positively charged at pH values below 8.8 and becomes negatively charged at pH values above 8.8. The adsorption tests were done at 25 °C and a pH range of 2 to 12. They used an initial dye concentration of 100 mg/L and an adsorbent dosage of 1.0 g/L for 180 min. The best removal efficiencies for AY36 and MR were 81.4% and 96.27%, respectively, at pH 2. The best removal efficiency for MB was 98.77% at pH 12. The greater adsorption of AY36 and MR in acidic conditions is due to the protonation of the amino, hydroxyl, and carboxyl functional groups on the RAB-A surface, creating positively charged active sites that make these anionic dyes more likely to bind. As the pH increased, the adsorption efficiencies of AY36 and MR decreased slowly. As the pH increased, the adsorption efficiencies of AY36 and MR decreased slowly. This was because the adsorbent surface lost protons lowering positive surface charge, and OH^−^ ions competed for the available adsorption sites. In contrast, methylene blue showed the opposite behavior during adsorption. The positively charged RAB-A surface repels the cationic MB molecules at very acidic pH. This makes the adsorption performance worse. At pH values above the pH_PZC_, the removal of protons from surface acidic groups gives the adsorbent a net negative charge. This makes it more likely to attract MB via electrostatic forces, thereby improving adsorption at alkaline pH. These results show that the pH-dependent surface charge properties of the adsorbent and the charge properties of the dye species in solution significantly affect the extent of RAB-A adsorption. This behavior aligns with prior studies indicating that the adsorption of anionic azo dyes is facilitated in acidic environments, whereas the removal of cationic dyes is improved in alkaline conditions.

#### Effect of adsorbent dosage

**Fig. 10:**
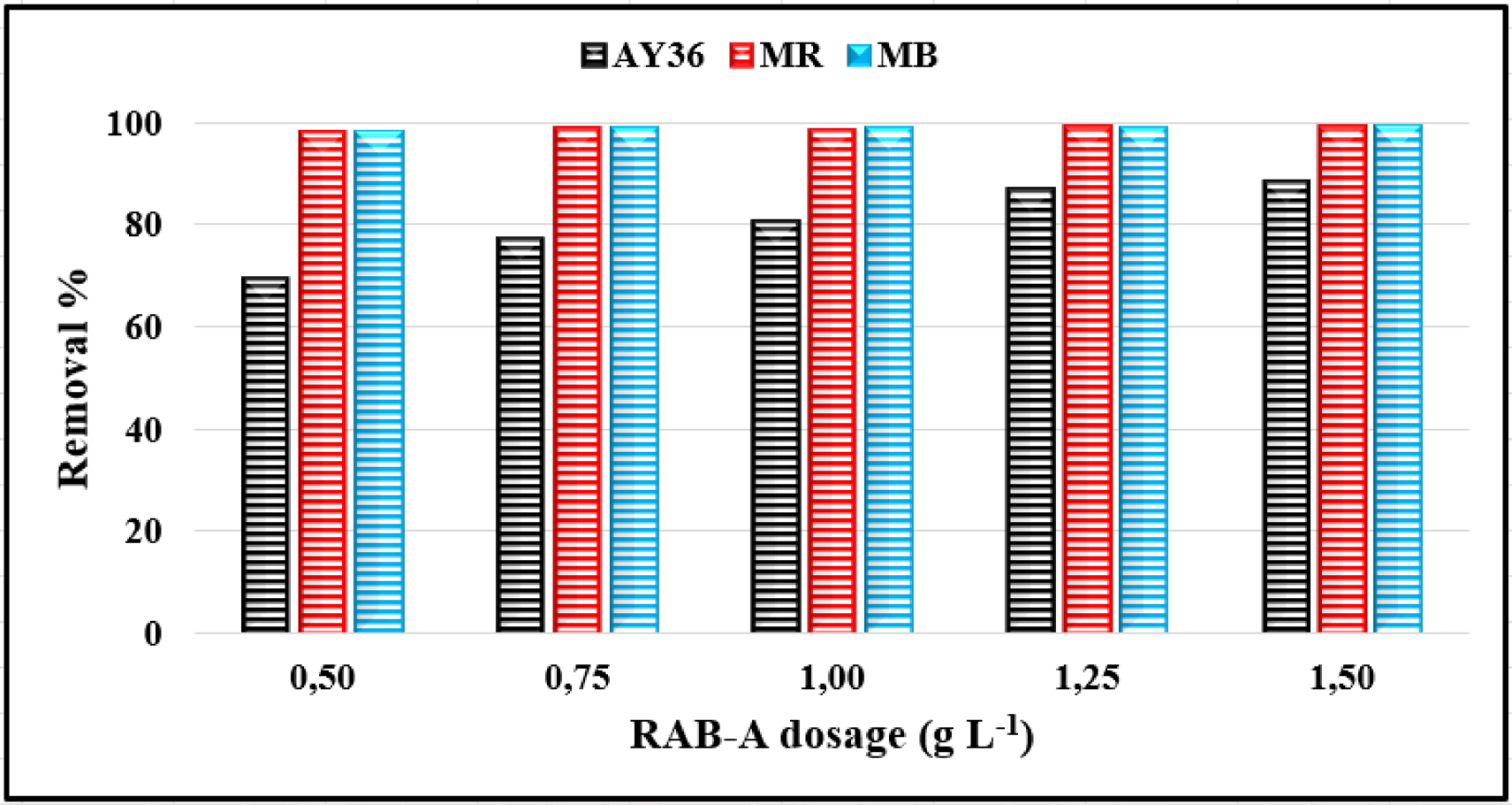
Effect of RAB-A dosage on adsorption of AY36, MR, and MB dyes (C_0_ = 100 mg/L; pH 2 for AY36/MR, pH 12 for MB; time = 180 min; T = 25 °C).

The amount of RAB-A applied plays a pivotal role in the adsorption process, as it determines the availability of active sites for interaction with the target dyes at a given initial concentration. In this study, the effect of varying RAB-A dosages on the removal efficiency of AY36, MR, and MB dyes was investigated while keeping all other experimental conditions unchanged. Figure 10 shows that increasing the adsorbent dosage enhanced the removal efficiency for all three dyes. The positive relationship between RAB-A amount and dye adsorption was particularly pronounced for AY36, whereas the enhancement was comparatively less significant for MR and MB. At an initial dye concentration of 100 mg/L, the maximum removal efficiencies were achieved with a RAB-A dosage of 1.5 g/L, reaching 88.43%, 99.25%, and 99.25% for AY36, MR, and MB, respectively.

The enhanced removal of MR and MB dyes is due to the greater number of accessible adsorption sites and the increased surface area associated with elevated RAB-A doses. Moreover, maintaining a constant dye concentration while increasing the adsorbent amount further improved adsorption performance by offering additional surface area for interactions between the dye molecules and the biochar^34,36^. According to the findings, a RAB-A concentration of 1.5 g/L was determined to be the most effective for achieving efficient removal of all the dyes evaluated.

#### Effect of initial solution concentration

**Fig. 11:**
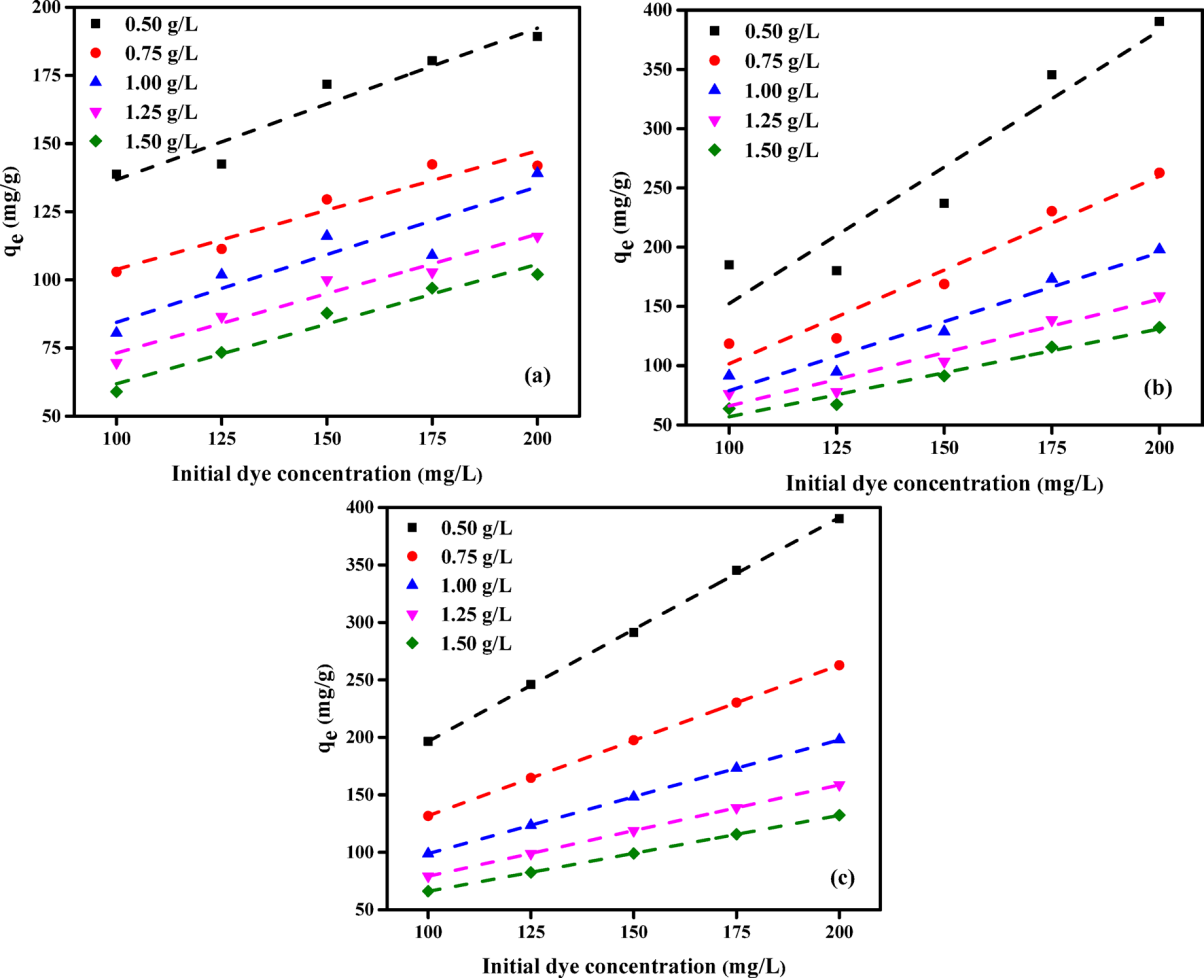
Effect of initial dye concentration on the adsorption capacity (*q*_e_) of RAB-A for (**a**) AY36, (**b**) MR, and (**c**) MB under 180 min contact time, at pH 2 for AY36 and MR, pH 12 for MB, and 25 °C.

This work investigated the effect of varying starting concentrations of AY36, MR, and MB dyes on the adsorption performance of RAB-A. Experiments were conducted with a constant contact time of 180 min, with pH set at 2 for AY36 and MR and at 12 for MB, employing initial dye concentrations of 100, 125, 150, 175, and 200 mg/L alongside RAB-A dosages ranging from 0.50 to 1.50 g/L. The experimental results are summarized in Fig. 11. An increase in adsorption capacity (*Q*_e_, mg/g) was observed for AY36 and MR as their initial concentrations increased, demonstrating a positive correlation. In contrast, MB dye adsorption exhibited an inverse trend. For example, at an RAB-A dosage of 0.50 g/L, *Q*_e_ rose from 138.77 to 189.32 mg/g when the initial concentration of AY36 increased from 100 to 200 mg/L. Likewise, adsorption capacities for RAB-A doses of 0.75, 1.00, 1.25, and 1.50 g/L ranged from 102.92 to 141.83, 80.48 to 139.11, 69.59 to 115.95, and 58.95 to 102.01 mg/g, respectively, across the corresponding concentration range. It was observed that raising the RAB-A dosage from 0.50 to 1.50 g/L at a constant AY36 concentration of 200 mg/L led to a reduction in the maximum adsorption capacity, decreasing from 189.32 to 102.01 mg/g (Fig. 11a).

The equilibrium adsorption capacity (*Q*_e_, mg/g) for MR dye rose from 185.10 to 390.41 mg/g as the starting dye concentration increased from 100 to 200 mg/L, while the RAB-A dosage remained constant at 0.5 g/L (Fig. 11b). Likewise, for RAB-A dosages of 0.75, 1.00, 1.25, and 1.50 g/L, *Q*_e_ values increased from 118.55 to 262.69, 91.46 to 197.96, 76.33 to 158.70, and 63.75 to 132.28 mg/g, respectively, across the same range of dye concentrations. In contrast, when the initial MR concentration was maintained at 200 mg/L, increasing the RAB-A dosage from 0.5 to 1.5 g/L resulted in a decline in the maximum adsorption capacity from 390.41 to 132.28 mg/g.

For MB dye, the equilibrium adsorption capacity (*Q*_e_, mg/g) increased from 196.43 to 390.41 mg/g when the RAB-A dosage was maintained at 0.5 g/L and the initial dye concentration was elevated from 100 to 200 mg/L (Fig. 11c). Likewise, for RAB-A dosages of 0.75, 1.00, 1.25, and 1.50 g/L, *Q*_e_ values rose from 131.60 to 262.69, 98.84 to 197.96, 79.25 to 158.70, and 66.16 to 132.28 mg/g, respectively, across the corresponding concentration range. In contrast, maintaining a constant starting MB concentration of 200 mg/L and increasing the RAB-A dosage from 0.5 to 1.5 g/L decreased the maximum adsorption capacity from 390.41 to 132.28 mg/g.

It is noteworthy that RAB-A consistently demonstrated a higher adsorption capacity for the MR dye than for the AY36 dye at all studied doses. These results indicate that adsorption efficiency is highly dependent on the initial dye concentration. This pattern is attributed to the limited number of active surface sites on the adsorbent, which may become saturated at high dye concentrations. At lower concentrations, the dye molecules have greater access to available adsorption sites, leading to more efficient adsorption.

#### Effect of contact time using RAB-A

**Fig. 12:**
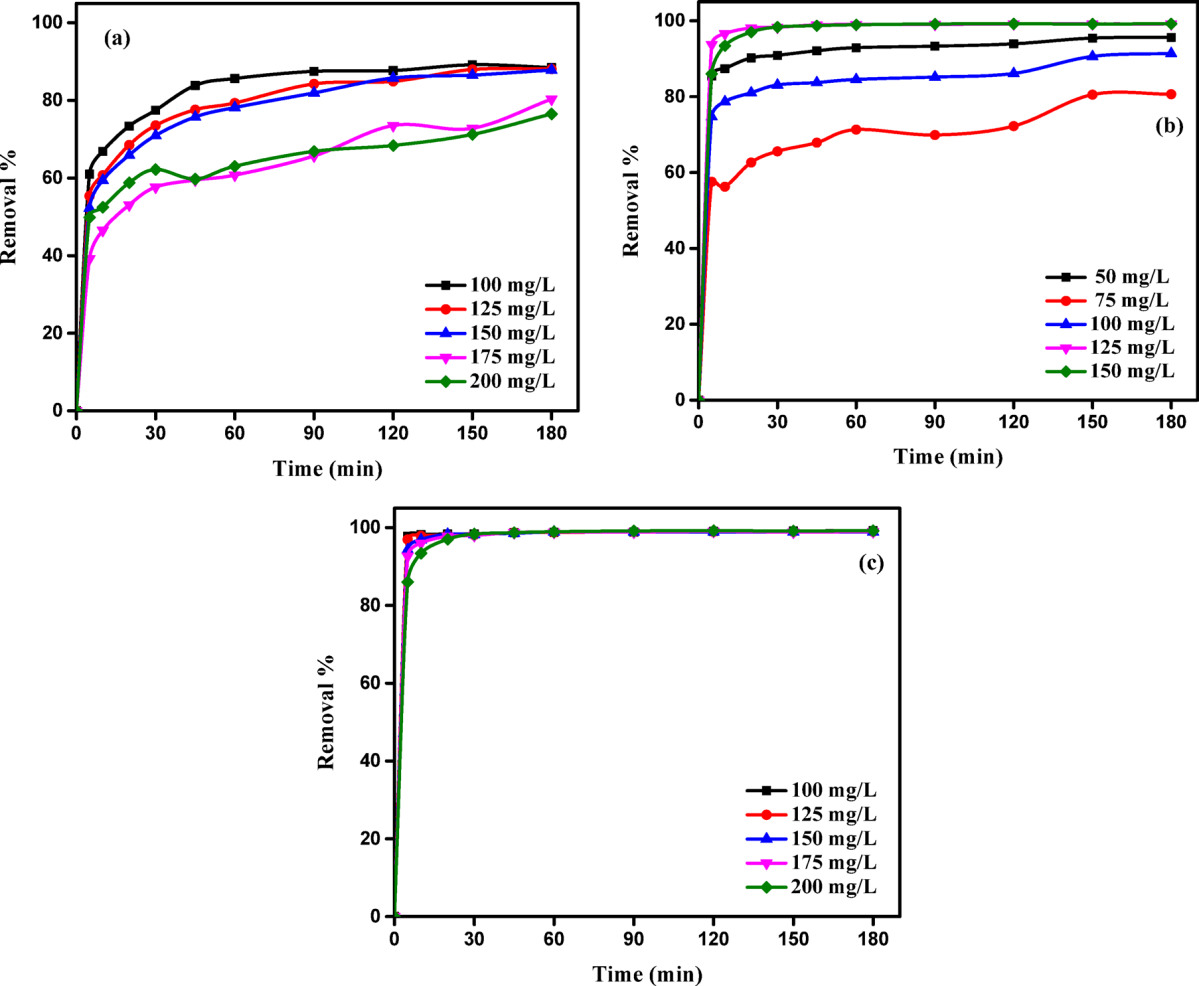
Effect of contact time on the adsorption of (**a**) AY36, (**b**) MR, and (**c**) MB onto RAB-A (adsorbent dose: 1.5 g/L, pH 2 for AY36 and MR, pH 12 for MB, 25 °C).

Duration of contact is a critical factor affecting mass transfer processes, such as adsorption. Figure 12 illustrates the impact of contact duration on the adsorption efficiencies of AY36, MR, and MB dyes by RAB-A during a 180-minute interval. As shown, the time to achieve equilibrium is influenced by both the type of dye and its initial concentration, with marked variations observed across dyes. In Fig. 12a, a substantial fraction of AY36 was removed during the initial five minutes. At an initial concentration of 100 mg/L, the removal efficiency reached 60.96%, whereas increasing the concentration to 200 mg/L resulted in a slightly lower efficiency of 49.86%. Following this rapid initial uptake, dye removal continued to increase substantially until approximately 45 min, after which the adsorption rate gradually declined, reaching equilibrium at around 180 min.

MR dye shows a similar trend as depicted in Fig. 12b. During the initial five minutes, a significant portion of MR was removed, achieving removal efficiencies of 85.44% and 86.03% at starting concentrations of 100 mg/L and 200 mg/L, respectively. A similar behavior was observed for MB dye (Fig. 12c), with rapid adsorption within the first 5 min, achieving removal efficiencies of 97.74% and 86.03% at 100 mg/L and 200 mg/L, respectively. Thereafter, the removal efficiency continued to increase gradually until reaching near-equilibrium at 180 min. At lower dye concentrations, the relatively large availability of adsorbent surface area per dye molecule diminished the effect of the initial concentration on adsorption. At higher concentrations, this percentage was reduced, resulting in a more pronounced dependence on the starting dye concentration. These findings suggest that dye removal efficiency is strongly linked to the initial concentration, in agreement with previous studies^24,77^.

### Adsorption isotherm studies

**Fig. 13:**
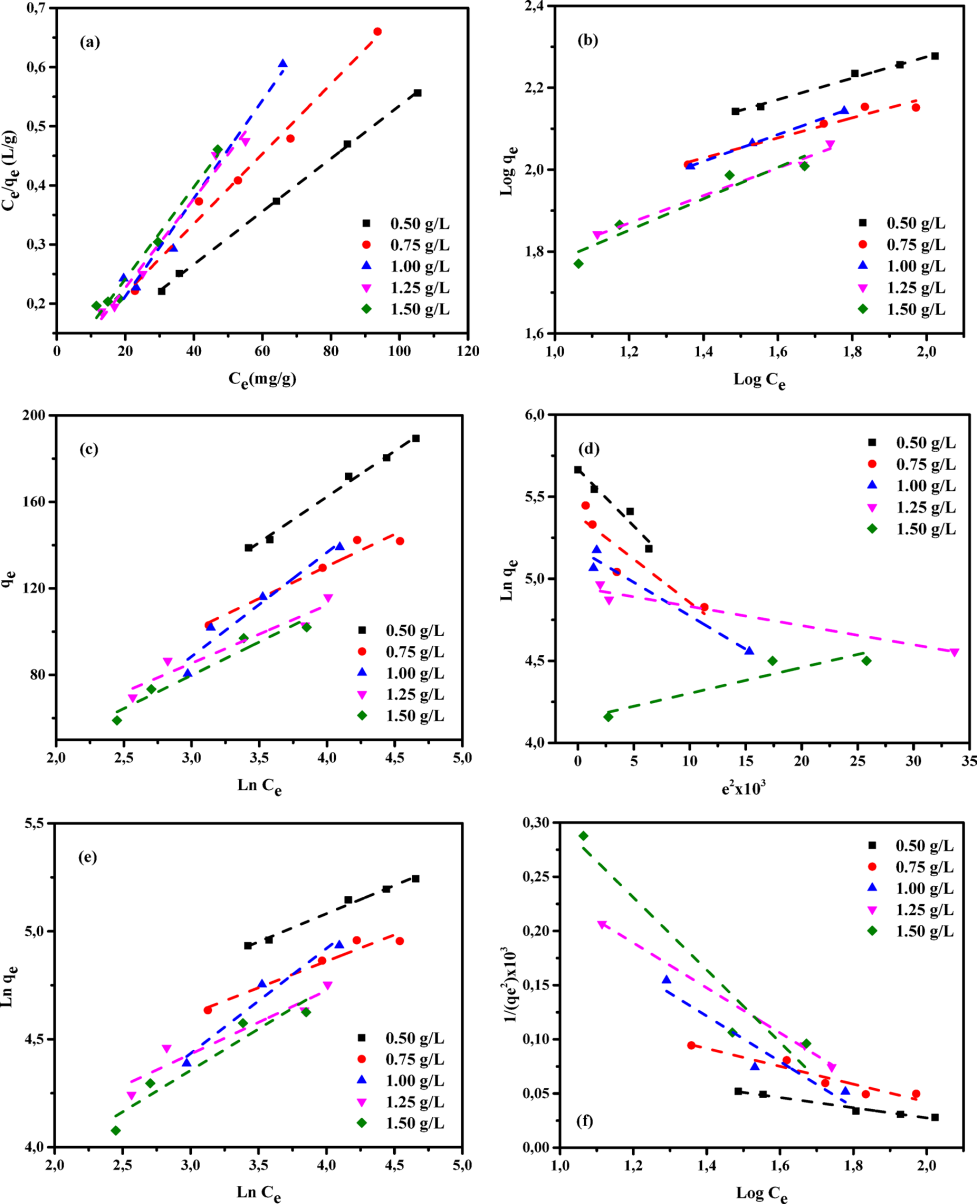
Adsorption isotherms of AY36 dye on RAB-A: (**a**) LIM, (**b**) FIM, (**c**) TIM, (**d**) DRIM, (**e**) HIM, and (**f**) HJIM (*C*₀ = 100–200 mg/L, adsorbent = 0.5–1.5 g/L, 25 °C, 180 min).

**Fig. 14:**
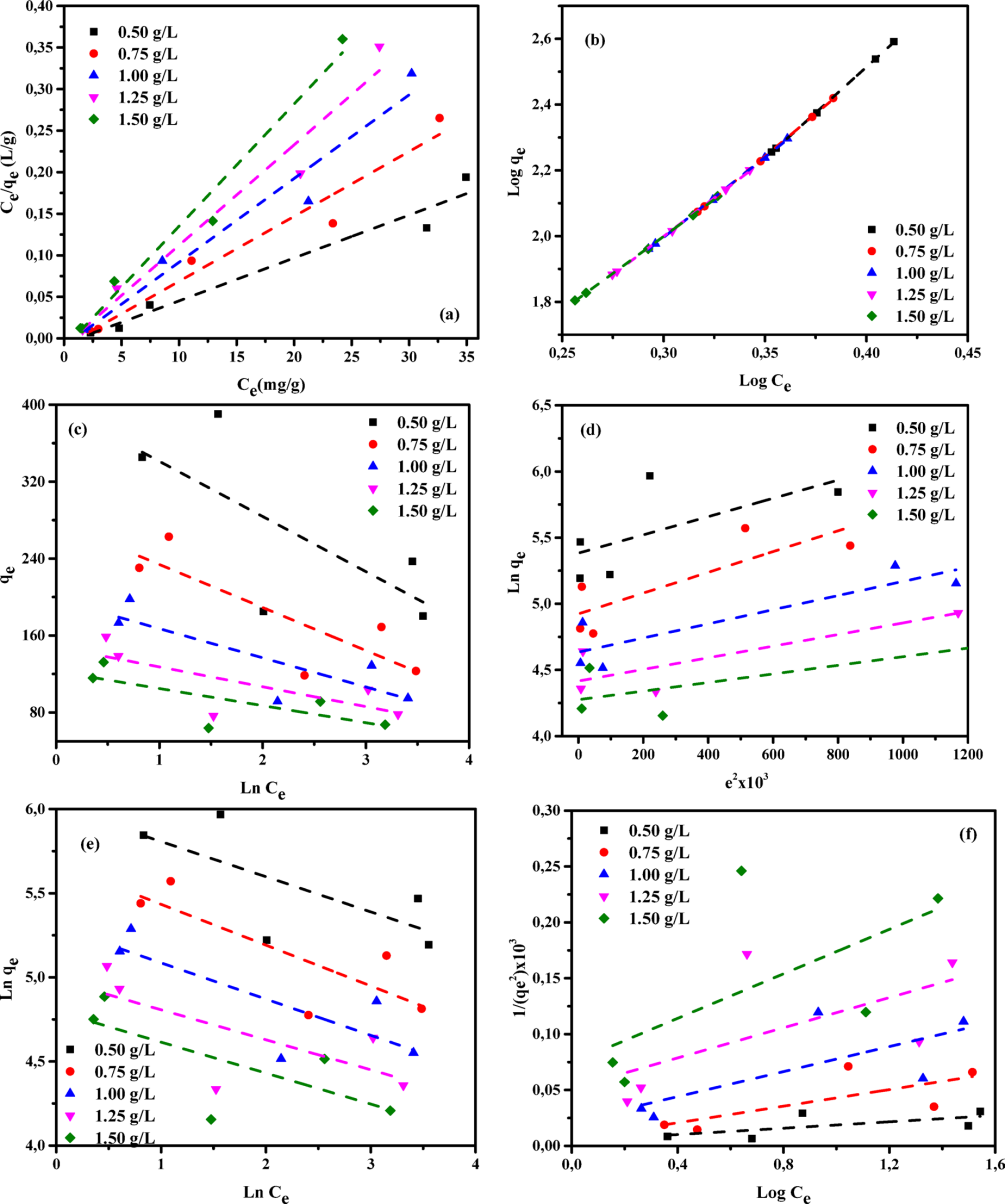
Adsorption isotherms of MR dye on RAB-A: (**a**) LIM, (**b**) FIM, (**c**) TIM, (**d**) DRIM, (**e**) HIM, and (**f**) HJIM (*C*_0_ = 100–200 mg/L, adsorbent = 0.5–1.5 g/L, 25 °C, 180 min).

**Fig. 15:**
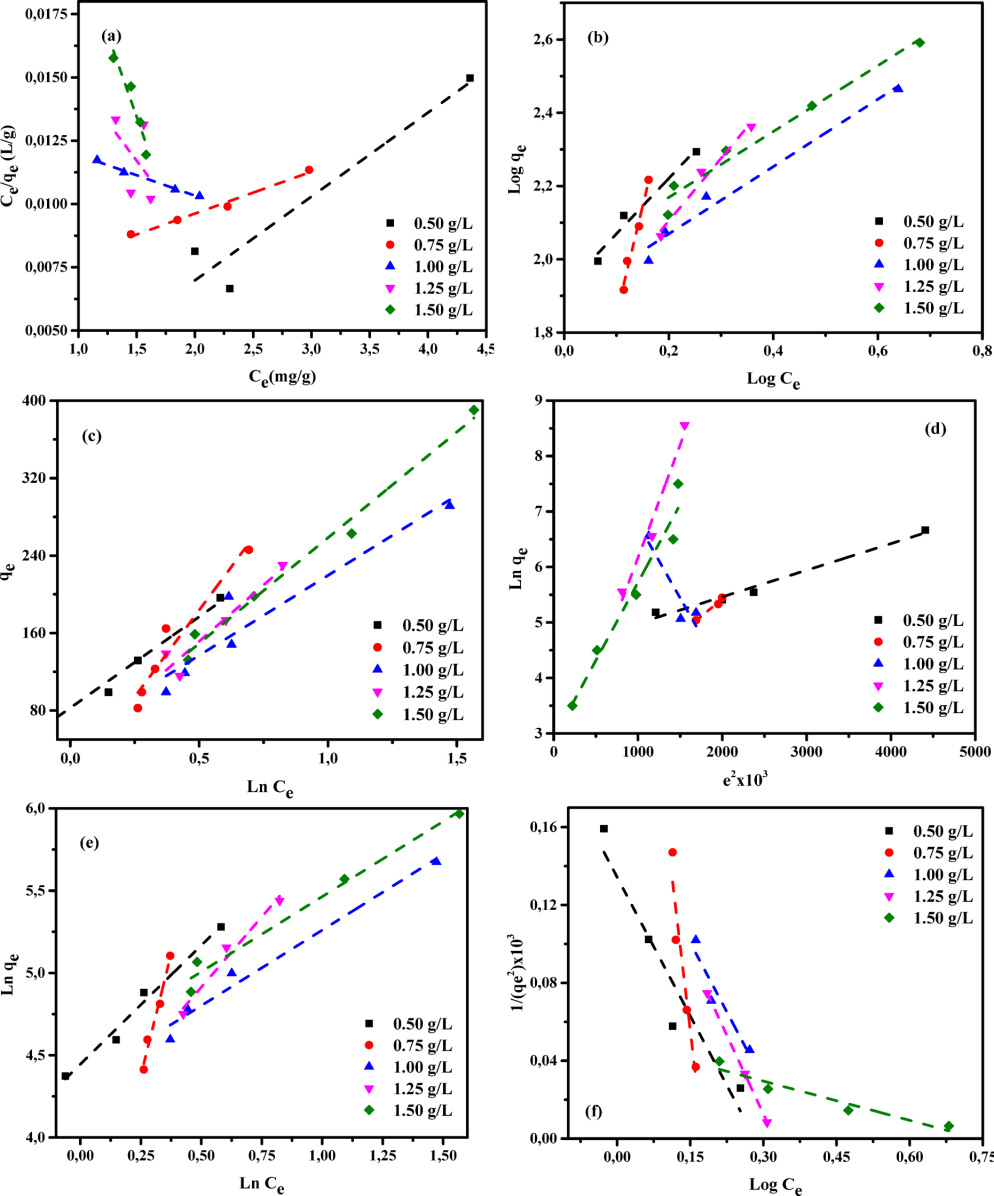
Adsorption isotherms of MB dye on RAB-A: (**a**) LIM, (**b**) FIM, (**c**) TIM, (**d**) DRIM, (**e**) HIM, and (**f**) HJIM (*C*_0_ = 100–200 mg/L, adsorbent = 0.5–1.5 g/L, 25 °C, 180 min).

Adsorption isotherms provide fundamental models for analyzing the processes by which substances migrate from aqueous solutions or porous materials onto solid surfaces under steady conditions^78,79^. Adsorption equilibrium is established when the quantity of adsorbate retained on the surface of the adsorbent no longer changes relative to its concentration in the surrounding liquid phase. This state is attained after an adequate contact time, allowing interactions between the adsorbent and adsorbate to stabilize. Under equilibrium conditions, the pollutant concentration in the bulk solution remains unchanged and is identical to that at the solid–liquid boundary^80,81^. The relationship describing the dependence of adsorbed mass on solute concentration in the aqueous phase was originally developed and experimentally confirmed by Shoaib et al.^76^. This concept continues to serve as a fundamental principle in adsorption research. It provides an essential framework for theoretical evaluations, guides process-optimization strategies, and underpins the engineering design of efficient contaminant-removal systems. As emphasized by Chen et al.^41^, a detailed assessment of thermodynamic aspects and physicochemical interactions is essential for elucidating adsorption mechanisms, particularly surface properties and the selectivity of adsorbents toward target pollutants. Consequently, the experimental results were interpreted using multiple adsorption isotherm models, including the Langmuir (LIM), Freundlich (FIM), Temkin (TIM), Dubinin–Radushkevich (DRIM), Halsey (HIM), and Harkin–Jura (HJIM) Eqs^42,82–84^. These six models were systematically examined to determine their applicability in describing the adsorption of AY36, MR, and MB dyes onto RAB-A. The resulting parameters are presented in Tables S1–S3 and depicted in Figs. 13, 14 and 15.

#### Langmuir isotherm

The Langmuir isotherm model (LIM), first introduced by Langmuir in 1916^85^, characterizes solute adsorption from the liquid phase onto a homogeneous adsorbent surface by assuming that adsorption occurs as a single molecular layer. In this model, adsorbed species do not interact with or migrate across the surface. In addition, the model assumes adsorption occurs at a finite set of energetically equivalent sites^35^. In this work, the LIM was applied to determine the maximum adsorption capacity (*Qm*, mg/g), which reflects the formation of complete monolayer coverage on the adsorbent surface. The corresponding mathematical expression of the LIM is presented in Eq. (4).

4 $$\:\frac{{C}_{e}}{{q}_{e}}=\frac{1}{{K}_{L}}{Q}_{m}+\frac{1}{{Q}_{m}}x{C}_{e}$$

where *Ce* (mg/L) refers to the equilibrium concentration of the adsorbate in the liquid phase, while *qe* (mg/g) corresponds to the amount adsorbed per unit mass of adsorbent at equilibrium. The parameter *K*_*L*_ (L/mg) is the Langmuir adsorption constant, and *Qm* (mg/g) defines the maximum adsorption capacity associated with monolayer formation.

The parameters associated with the LIM are compiled in Tables S1–S3, and the corresponding linear Langmuir plots for AY36, MR, and MB dyes are presented in Figs. 13a, 14a, and 15a, respectively. The adsorption behavior of RAB-A was well described by the LIM, as reflected by the high determination coefficients (*R*^2^), which varied between 0.972 and 1.00 for AY36, 0.958 and 0.978 for MR, and 0.922 and 0.992 for MB. These results demonstrate that RAB-A exhibits effective adsorption characteristics toward all dyes investigated. The estimated monolayer adsorption capacities (*Q*_*m*_) were 222.22 mg/g for AY36, 192.31 mg/g for MR, and 833.33 mg/g for MB. The LIM parameters (1/*Q*_m_ and 1/*Q*_*m*_*K*_*L*_) were derived from the slope and intercept values obtained from the linear plots of *Ce/q*_e_ versus *Ce*, as illustrated in Figs. 13a, 14a, and 15a. The obtained *K*_*L*_ values varied between 0.0515 and 0.1789 L/mg for AY36, 0.0008 and 0.0014 L/mg for MR, and 0.0001 and 0.0083 L/mg for MB, reflecting favorable adsorbate–adsorbent interactions on the RAB-A surface. Collectively, these findings demonstrate that the LIM provides an excellent description of AY36 adsorption onto RAB-A, while its suitability for MR and MB is comparatively weaker. Furthermore, the results support the predominance of monolayer coverage for all dyes on the RAB-A surface.

#### Freundlich-isotherme

This work first applied the Freundlich isotherm model (FIM)^86^ to evaluate adsorption characteristics. As an empirical model with an exponential relationship, the FIM describes a proportional increase in surface adsorption as the solute concentration in the liquid phase rises. The governing equation for this model is given by Eq. (5).

5 $$\:Ln\:{q}_{e=\:}\mathrm{ln}{K}_{F}+\frac{1}{n}\mathrm{ln}{C}_{e}$$

where *K*_F_ and *n* (mg^1–1/n^ g^–1^ L^1/n^) denote the FIM constants, representing the capacity and intensity of adsorption, respectively.

The FIM parameters derived in this study are summarized in Tables S1–S3, and the corresponding FIM plots for AY36, MR, and MB dyes are depicted in Figs. 13b, 14b, and 15b, respectively. The FIM parameters, *K*_F_ and 1/*n*, were calculated from the slope and intercept values derived from the linear plots of ln(*q*_e_) versus ln(*C*_e_) shown in the corresponding figures. The constant *K*_F_ (L/g) represents the adsorption capacity and interaction strength between the RAB-A adsorbent and the dye molecules at equilibrium. According to Fan et al.^87^, values of 1/*n* lower than unity are indicative of favorable adsorption and enhanced adsorbent–adsorbate affinity. In the present work, the 1/*n* values reported in Tables S1–S3 are below one for AY36, suggesting efficient adsorption dominated by physical interactions, whereas such behavior was not observed for MR and MB dyes. Correlation coefficients (R²) for the FIM model were obtained from the linear ln(*q*_e_)–ln(*C*_e_) plots shown in Figs. 13b, 14b, and 15b. Notably, RAB-A showed greater conformity with the FIM model for MR and MB dyes, with higher *R*^2^ values (0.999–1.000 for MR and 0.971–0.979 for MB) than those obtained with the LIM, indicating a superior fit. For AY36 dye, *R*^2^ values ranged from 0.907 to 0.971, indicating a better fit with the LIM.

#### Temkin isotherm

The Temkin isotherm model (TIM)^88^ characterizes adsorption by considering indirect interactions between adsorbent and adsorbate molecules. According to this model, the adsorption heat decreases linearly with increasing surface coverage, reflecting the influence of adsorbate–adsorbent interactions, while the associated binding energies are assumed to be uniformly distributed up to a defined upper limit. The TIM is mathematically represented in Eq. (6) and is often reformulated into a more convenient linear expression, as shown in Eq. (7)^35^.

6 $$\:{Q}_{e}=\frac{RT}{{B}_{T}}\mathrm{l}\mathrm{n}\left({A}_{T}{C}_{e}\right)$$

7 $$\:{Q}_{e}=\frac{RT}{{B}_{T}}\mathrm{ln}\left({A}_{T}\right)+\frac{RT}{{B}_{T}}\mathrm{l}\mathrm{n}\left({C}_{e}\right)$$

where *A*_T_ (L/mg) corresponds to the TIM constant, whereas *B*_T_ (J g mol^–1^ mg^–1^) represents the factor associated with variations in adsorption energy (heat of adsorption). In addition, *R* (8.314 J mol^–1^ K^–1^) is the universal gas constant, and *T* (K) refers to the absolute temperature.

The TIM parameters, *A*_*T*_ and *B*_*T*_, were obtained from the linear dependence of *q*_e_ on ln *C*_e_, as shown in Figs. 13c, 14c, and 15c. In this linear representation, the slope yields the *A*_*T*_ constant (g/L), whereas the intercept corresponds to *B*_*T*_. A detailed compilation of the resulting TIM constants is provided in Tables S1–S3. The derived correlation coefficients demonstrate a strong fit between the model and experimental data across the investigated temperature range, with *R*^2^ values of 0.987–1.000 for AY36 and 0.912–0.987 for MB dye, thereby confirming the applicability of the TIM to the adsorption behavior. The same interpretation is not possible for the MR dye, and *R*^2^ values ranging from 0.510 to 0.700 did not show a good fit for this model. The parameter *B*_T_, which reflects the adsorption heat and the energy liberated through interactions between the adsorbate and the adsorbent, is crucial for interpreting the underlying adsorption mechanism. The *B*_T_ values obtained for AY36 dye suggest that its adsorption onto the RAB-A surface occurs at relatively low energies, consistent with a predominantly physical adsorption process. In contrast, higher *B*_T_ values for the MR and MB dyes indicate that their adsorption is mainly governed by chemical interactions.

#### The Dubinin–Radushkevich (DRIM) isotherm

The Dubinin–Radushkevich isotherm model (DRIM) is commonly used to evaluate the apparent free energy governing adsorption processes and to characterize the porous nature of the adsorbent material^37,89^. Unlike isotherm models that assume a homogeneous surface or constant adsorption energy, the DRIM employs a more generalized approach that eliminates the need for such assumptions. The original expression of this model is presented in Eq. (8), whereas its linearized form is provided in Eq. (9).

8 $$\:{q}_{e}={Q}_{m}\mathrm{e}\mathrm{x}\mathrm{p}(-K{\epsilon\:}^{2})$$

9 $$\:ln{q}_{e}=ln{Q}_{m}-{K\epsilon\:}^{2}$$

where *Q*_*m*_ (mg/g) represents the theoretical maximum adsorption capacity at saturation, *K* (mol^2^ (kJ^2^)^–1^) is a constant associated with adsorption energy, and ε denotes the Polanyi potential, which is determined according to Eq. (10):

10 $${\varepsilon}=\mathrm{R}\mathrm{T}\times\:\mathrm{l}\mathrm{n}(1+\frac{1}{{\mathrm{C}}_{e}})$$

For the linearized DRIM, the constant *K* (mol²·kJ⁻²) is obtained from the slope of the *q*_e_ versus ε² plot, while the intercept corresponds to the maximum adsorption capacity, *Q*_*m*_ (mg/g). Based on the derived *K* value, the mean adsorption free energy (*E*)—defined as the energy necessary to transfer one mole of solute from the bulk solution to the adsorbent surface—was calculated using Eq. (11).

11 $$\:E=1/\sqrt{\left(2K\right)}$$

The DRIM was applied to the equilibrium data to assess whether the adsorption of AY36, MR, and MB dyes onto RAB-A is governed predominantly by physical or chemical interactions. Within the framework of Polanyi’s potential theory underlying this model, adsorption proceeds until the adsorbent’s pore structure is saturated. The correlation coefficients and DRIM constants that were determined at various adsorbent concentrations are summarized in Tables S1–S3. The mean adsorption energy (E) is a critical metric for distinguishing between adsorption mechanisms. According to commonly accepted criteria, mean adsorption energy (*E*) values below 8 kJ/mol are indicative of physisorption, values in the range of 8–16 kJ/mol are associated with ion-exchange mechanisms, whereas values exceeding 16 kJ/mol reflect chemisorption behavior^37,89^. Analysis of the calculated *E* values shows that, across all tested dosages, AY36 dye exhibits *E* > 8 kJ/mol, MR dye has *E* > 16 kJ/mol, and MB dye displays *E* < 8 kJ/mol. These results indicate that AY36 adsorption involves both ion exchange and chemical interactions. MR adsorption is primarily chemical, and MB adsorption is predominantly physical. Correlation coefficients (*R*^2^) from the DRIM ranged from 0.951 to 0.991 for AY36 dye and from 0.856 to 0.982 for MB dye, demonstrating good agreement with the experimental data. In contrast, MR dye showed lower *R*^2^ values (0.420–0.801), indicating a poor fit (Figs. 13d, 14d, and 15d; Tables S1–S3). The highest monolayer adsorption capacities (*Q*_*m*_) of RAB-A were determined as 307.60 mg/g for AY36 dye, 217.67 mg/g for MR dye, and 128.02 mg/g for MB dye.

#### Halsey isotherm

The Halsey isotherm (HIM) model is applicable for characterizing multilayer adsorption systems, such as metal ions or dyes adsorbing at considerable distances from the surface^90,91^, with the adsorption capacity calculated using Eq. (12).

12 $$\:\mathrm{ln}\left({q}_{e}\right)=\left[\left(\frac{1}{{n}_{H}}\right)ln\left({K}_{H}\right)\right]-\left(\frac{1}{{n}_{H}}\right)ln\left(\frac{1}{{C}_{e}}\right)$$

Where *K*_*H*_ and *n*_*H*_ represent the HIM constants, which can be determined from the slope and intercept, respectively, of the linear plot of ln(q_e_) versus ln (C_e_).

Figures 13e, 14e, and 15e depict the HIM for the adsorption of AY36, MR, and MB dyes onto RAB-A. The corresponding HIM parameters were determined and are presented in Tables S1–S3. In Tables S1-S3, it can be seen that the R2 values are centered at 0.933–0.969 for the AY36 dye and 0.973–0.983 for the MB dye, indicating good agreement with adsorption on RAB-A. It is not possible to make the same interpretation for the MR dye, as its values remain within the range of 0.493–0.660.

#### Harkins-Jura isotherm

According to previous reports^90,91^, the Harkins–Jura isotherm model (HJIM) primarily characterizes multilayer adsorption and the presence of heterogeneous pore distributions on adsorbent surfaces, and it can be expressed as Eq. (13):

13 $$\:\left[\frac{1}{{q}_{e}^{2}}\right]=\left[\frac{{B}_{HJ}}{{A}_{HJ}}\right]-\left[\frac{1}{{A}_{HJ}}\right]log\left({C}_{e}\right)$$

Here, *B*_*HJ*_ and *A*_*HJ*_ are the HJIM constants, which can be obtained from the slope and intercept, respectively, of the linear plot of 1/q_e_² versus log(C_e_). The HJIM model for the adsorption of AY36, MR, and MB dyes onto RAB-A is illustrated in Figs. 13f, 14f, and 15f, with the corresponding isotherm parameters calculated and summarized in Tables S1–S3. In Tables S1-S3, the *R*^2^ values are centered at 0.933–0.969 for the AY36 dye and 0.973–0.983 for the MB dye, indicating good agreement with adsorption on RAB-A. It is not possible to make the same interpretation for the MR dye, as its values remain within the range of 0.493–0.660. However, since these MR dye values fall within the range of 0.399 to 0.542, they do not conform to the multilayer adsorption rule.

These results indicate that the DRIM provides a comparatively weaker description of AY36 adsorption behavior, especially compared with the LIM and TIM isotherm models, whereas the FIM model provides a more suitable description of the adsorption of MR and MB dyes.

### Examination of error functions for the optimal isotherm model

To identify the isotherm model that most accurately represents the adsorption of AY36, MR, and MB dyes onto RAB-A, the correlation coefficients (R²) obtained from the LIM, FIM, TIM, DRIM, HIM, and HJIM models were compared against the experimental equilibrium data. Alongside *R*^2^, model performance was assessed through various error metrics, including average percent error (APE), average relative error (ARE), chi-square (χ^2^), sum of squared errors (ERRSQ), hybrid error function (HYBRID), Marquardt’s percent standard deviation (MPSD), sum of absolute errors (EABS), and root mean square error (RMS), which collectively measure the discrepancy between predicted and experimental values^92^. The comparative assessment of these error metrics is presented in Table S4. Analysis of the results indicates that, based on error functions, FIM best describes AY36 adsorption, DRIM provides the optimal fit for MR, and HJIM offers the best representation for MB, as evidenced by the lowest values across all error criteria (APE, ERRSQ, χ^2^, RMS, EABS, ARE, HYBRID, and MPSD). While correlation coefficients suggest that LIM and TIM also closely match the AY36 data, error-function analysis indicates that FIM is the superior model. For MR and MB dyes, FIM showed the highest correlation coefficients, whereas DRIM for MR and HJIM for MB were identified as the most accurate models based on error function evaluation^57^.

### Adsorption kinetic studies

**Fig. 16:**
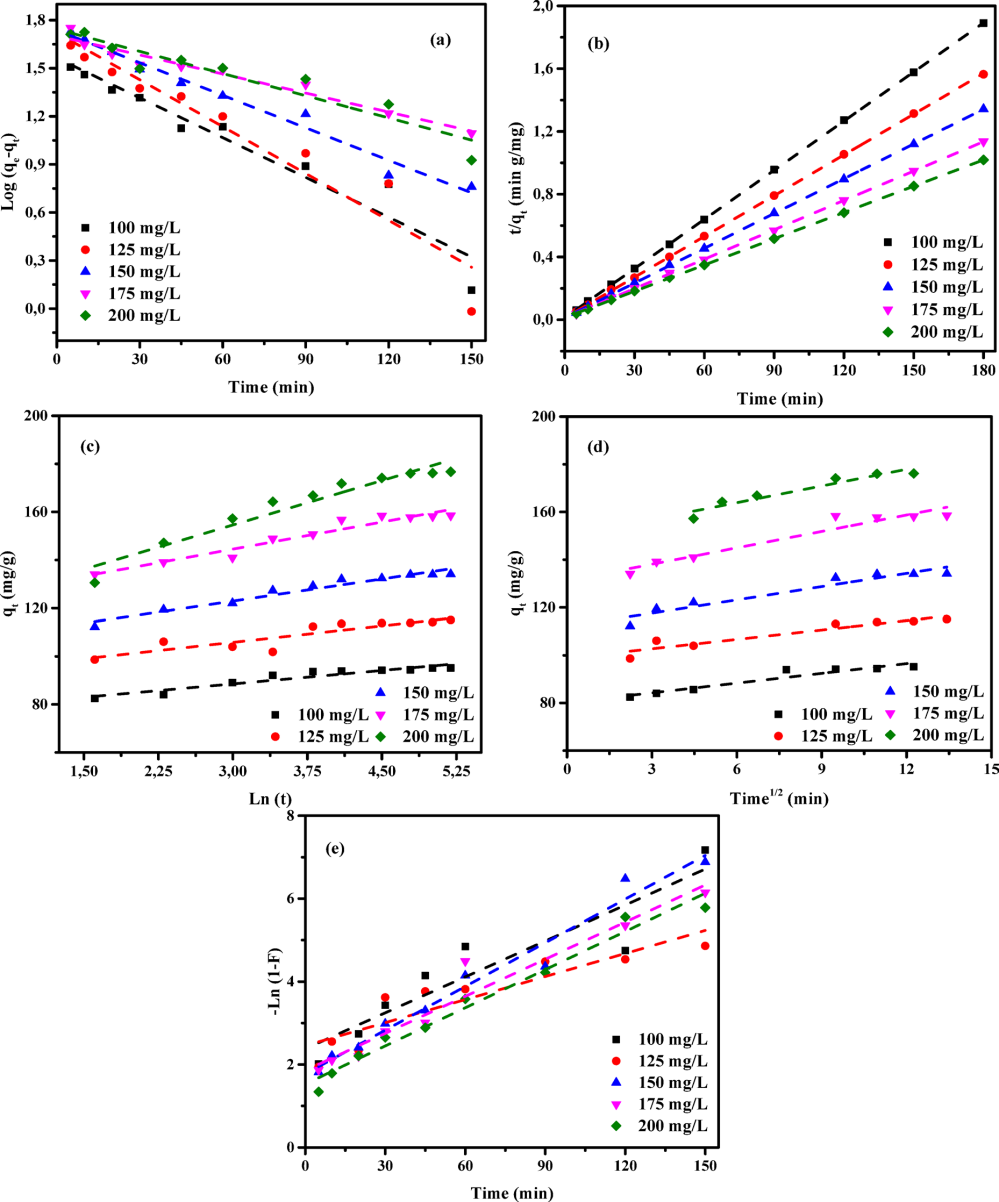
The plot of (**a**) PFOM, (**b**) PSOM, (**c**) EM, (**d**) IPDM, and (**e**) FDM of adsorption of AY36 dye by RAB-A adsorbent (*C*_0,_ dye = (100–200 mg/L), *C*_*0*_, RAB-A = (1.0 g/L), Temp. = 25 °C).

**Fig. 17:**
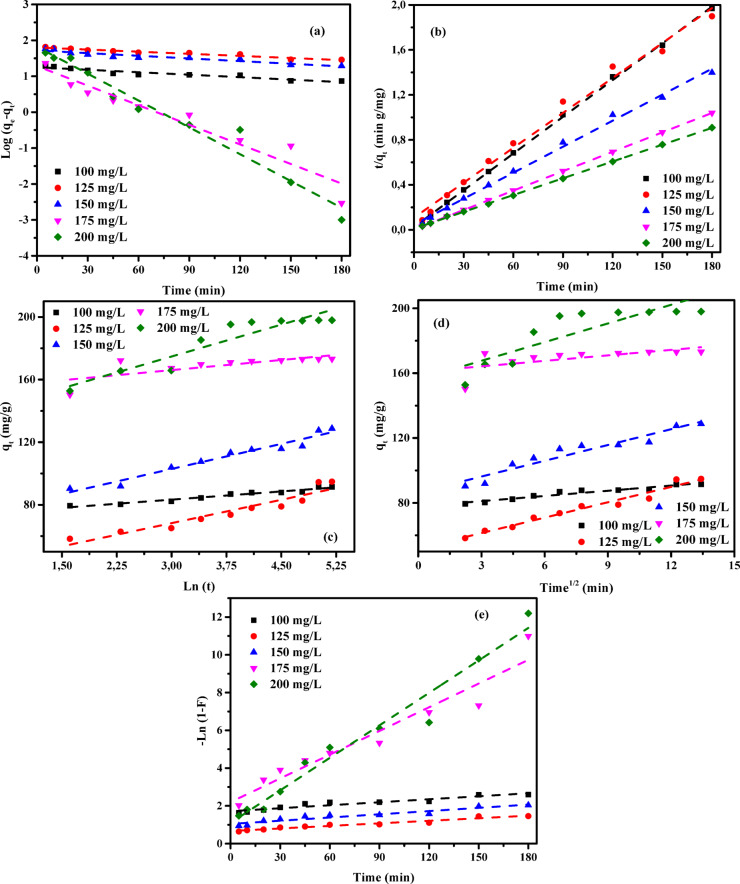
The plot of (**a**) PFOM, (**b**) PSOM, (**c**) EM, (**d**) IPDM, and (**e**) FDM of adsorption of MR dye by RAB-A adsorbent (*C*_0,_ MR dye = (100–200 mg/L), *C*_0_, RAB-A = (1.0 g/L), Temp. = 25 °C).

**Fig. 18:**
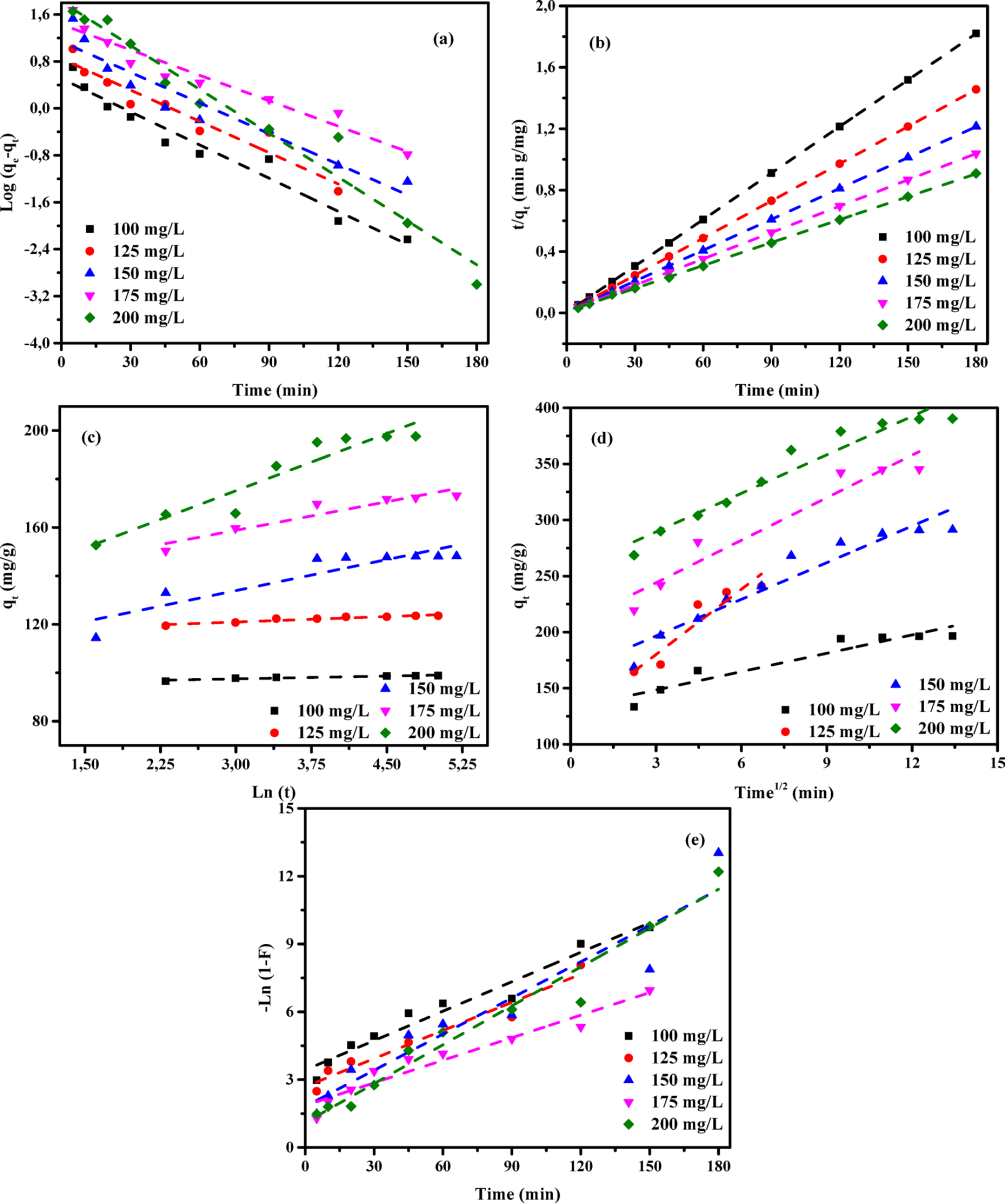
The plot of (**a**) PFOM, (**b**) PSOM, (**c**) EM, (**d**) IPDM, and (**e**) FDM of adsorption of MB dye by RAB-A adsorbent (*C*_0,_ MR dye = (100–200 mg/L), *C*_0_, RAB-A = (1.0 g/L), Temp. = 25 °C).

An in-depth investigation of the adsorption kinetics of AY36, MR, and MB dyes onto RAB-A was performed using multiple models encompassing diffusion, mass transfer, and chemical reaction mechanisms, to determine the optimal conditions for potential batch-scale implementation^57^. A comprehensive assessment of kinetic parameters is essential for reliable determination of adsorption rates and is fundamental to the modeling, optimization, and rational development of adsorption systems. Accordingly, several kinetic models—namely the pseudo-first-order (PFOM)^93^, pseudo-second-order (PSOM)^94^, Elovich model (EM)^76^, Intraparticle diffusion model (IDM)^35^, and film diffusion model (FDM)^34^—were applied to evaluate the removal performance of AY36, MR, and MB dyes using RAB-A. The consistency between experimental data and model predictions was assessed using the correlation coefficient (*R*^2^), and the results are presented in Figs. 16, 17, 18, and summarized in Tables S5-S10.

#### Pseudo-first-order model (PFOM)

The adsorption rate constant was determined using the Lagergren pseudo-first-order model (PFOM)^95^, which serves as an initial approach for characterizing adsorption kinetics in terms of adsorption capacity. The corresponding PFOM kinetic expression is presented in Eq. (14).

14 $$\:\mathrm{log}\left({q}_{\mathrm{e}}-{q}_{\mathrm{t}}\right)=\mathrm{log}\left({q}_{\mathrm{e}}\right)-\frac{{k}_{1}}{2.303}t$$

The variables *q*_t_ and *q*_e_ (mg/g) represent the amounts of ions adsorbed at a given contact time (t) and at equilibrium, respectively. The constant *k*_*1*_ (min^–1^) corresponds to the adsorption rate parameter for PFOM. Values of *k*_*1*_ and *q*_*e*_ were derived from the slope and intercept of the linear plots of log (*q*_*e*_-*q*_*t*_) versus time (*t*), as illustrated in Figs. 16a, 17a, and 18a. As summarized in Tables S5, S7, and S9, the correlation coefficients (*R*^2^) for this kinetic model range from 0 to 1, with higher values indicating a better fit. The consistently high *R*² values, predominantly above 0.850, confirm strong agreement between the experimentally measured and model-predicted adsorption capacities (*q*_*e*_). The predominantly high R^2^ values, exceeding 0.850, demonstrate a strong consistency between the calculated and experimental adsorption capacities (*q*_*e*_). Although a few lower *R*^2^ values (≥ 0.734) were observed for the MR dye, the overall results confirm that the PFOM adequately represents the adsorption kinetics of all dyes on RAB-A. Moreover, the data reported in Tables S5, S7, and S9 reveal no systematic increase or decrease in R^2^ with increasing RAB-A dosage within the 0.5–1.5 g/L range for any of the dyes.

#### Pseudo-second-order model (PSOM)

In the present investigation, the pseudo-second-order model (PSOM) was employed to assess the adsorption behavior of RAB-A toward AY36, MR, and MB dyes. According to the PSOM’s underlying assumption, the adsorption rate is governed by the square of the concentration of available active sites on the adsorbent surface. Furthermore, the overall adsorption kinetics are significantly influenced by the quantity of adsorbate that accumulates on the surface. Mathematically, the PSOM is represented in Eq. (15).

15 $$\:\left(\frac{t}{{q}_{t}}\right)=\frac{1}{{k}_{2}{q}_{e}^{2}}+\frac{1}{{q}_{e}}\left(t\right)$$

where *k*_2_ (g mg^–1^ min^–1^) denotes the equilibrium rate constant for PSOM adsorption.

When the PSOM adequately describes the system, a linear relationship between *t*/*q*_*t*_ and *t* is observed. This linear behavior allows the kinetic rate constant (*k*_2_) and the equilibrium adsorption capacity (*q*_e_) to be determined from the slope and intercept of the relevant plots, respectively, as demonstrated in Figs. 16(b), 17(b), and 18(b) for the adsorption of AY36, MR, and MB dyes onto RAB-A. The PSOM kinetic parameters, including *k*_2_, the calculated and experimental *q*_*e*_ values, and the associated correlation coefficients (*R*^2^), are summarized in Tables S5, S7, and S9. The analysis shows that the *R*^2^ values obtained from the PSOM are very close to unity, indicating strong agreement between the predicted and experimentally measured adsorption capacities across the investigated initial dye concentrations.

The adsorption of organic dyes generally proceeds through a combination of physical interactions, such as electrostatic attraction, π–π interactions, complex formation, hydrogen bonding, pore filling, and network entanglement. In acidic media, the adsorption of anionic dyes onto the adsorbent is mainly controlled by electrostatic attractions resulting from the protonation of amino functional groups. Conversely, at alkaline pH, suppressed protonation of –NH_3_^+^ groups reduces electrostatic attraction, leading to adsorption dominated by chemical interactions^96^. Collectively, these results demonstrate that the PSOM accurately captures the adsorption kinetics of all three dyes on RAB-A and represents the most suitable kinetic model among those examined.

#### Elovich kinetic model (EM)

The Elovich model (EM) was employed to analyze the adsorption behavior of AY36, MR, and MB dyes on RAB-A. Although the model was originally proposed for gas-phase adsorption, it has since been widely and effectively adapted to liquid-phase systems, particularly in wastewater treatment applications. The EM provides important information on changes in activation and deactivation energies, as well as on mass transfer and particle transport phenomena at the solid–liquid interface. According to the theoretical approaches described by Cheung et al. (2000)^97^ and Dotto and Pinto (2011)^98^, the adsorption rate declines exponentially with increasing solute uptake. The EM is therefore well suited for characterizing surface heterogeneity of the adsorbent, and its corresponding mathematical expression is presented in Eq. (16).

16 $$\:{q}_{\mathrm{t}}=\frac{1}{\beta\:}\mathrm{ln}\left(\alpha\:\beta\:\right)+\frac{1}{\beta\:}\mathrm{l}\mathrm{n}\left(t\right)$$

where *α* (mg g^–1^ min^–1^) represents the initial adsorption rate constant, whereas *β* (g mg^–1^) is associated with surface coverage and the activation energy of the chemisorption process. The EM kinetic parameters were determined from the slope and intercept of the linearized plots of $\:{q}_{t}$versus $\:\mathrm{ln}t,$ plots, as shown in Figs. 16c, 17c, and 18c, with the corresponding values summarized in Tables S6, S8, and S10. The correlation coefficients (*R*^2^) obtained for the adsorption of AY36, MR, and MB dyes onto RAB-A ranged from 0.903 to 0.998, 0.549 to 0.986, and 0.829 to 0.983, respectively, indicating an overall satisfactory agreement between the EM predictions and experimental data, particularly for AY36 and MB dyes. However, this level of agreement was not consistently observed for MR dyes. A comparative evaluation of the R² values further shows that, for AY36 adsorption, the EM yielded a slightly better fit than the PFOM, although it remained less accurate than the PSOM, as summarized in Tables S5–S10. For the MR and MB dyes, EM had the lowest fit. Overall, the results suggest that the adsorption of AY36, MR, and MB dyes onto RAB-A is predominantly chemisorption-driven.

#### Intra-particle diffusion model (IPDM)

Pollutant elimination by solid adsorbents proceeds through a sequence of mass-transfer and surface-interaction steps. Initially, contaminants are transported from the bulk solution to the adsorbent’s outer surface via bulk diffusion. Subsequently, film diffusion governs the passage of pollutants across the liquid boundary layer enveloping the adsorbent particles. The next stage involves intraparticle (pore) diffusion, through which pollutants penetrate the internal pore network and interact with internal surfaces. In the final stage, adsorption occurs at the adsorbent’s accessible active sites via physicochemical interactions, such as ion exchange, chelation, and surface complexation^57^. To elucidate mass transfer between the aqueous phase and the adsorbent, the IPDM was employed as described by Kargi and Cikla^99^. Under batch-operating conditions with intense mixing, intraparticle diffusion in an isothermal plug-flow regime may be the rate-determining step governing overall adsorption kinetics^100^. Further validation of the IPDM under these conditions was achieved by applying Eq. (17), originally proposed by Annadurai et al.^101^.

17 $$\:{q}_{t}={K}_{diff}{t}^{0.5}+C$$

where *K*_*diff*_ signifies the rate constant of IPDM (mg g^–1^ min^1/2^) (Figs. 16d, 17d, and 18d).

According to the Weber and Morris IPDM^102^, adsorption is predominantly governed by intraparticle diffusion when the linear plot of $\:{q}_{t}$ and $\:{t}^{1/2}$ intersects the origin, as illustrated in Figs. Figs. 16d, 17d, and 18d. In contrast, when the plots exhibit a noticeable deviation from the origin—particularly characterized by a large intercept (*C*)—the adsorption rate is typically controlled by film diffusion (FDM). In the present work, the adsorption performance of RAB-A toward AY36, MR, and MB dyes was systematically evaluated under varying operational parameters, including adsorbent dosage and initial dye concentration. The Weber–Morris intraparticle diffusion profiles for the adsorption of AY36, MR, and MB dyes are presented in Figs. 16d, 17d, and 18d, respectively. The IPDM rate constants ($\:{K}_{\mathrm{dif}}$) and intercept values (*C*) were extracted from the slopes and intercepts of the linear $\:{q}_{t}$versus $\:{t}^{1/2}\:$plots, with the calculated parameters summarized in Tables S6, S8, and S10. For all three dyes, none of the diffusion lines obtained at different adsorbent dosages intersected the origin, indicating a significant boundary-layer effect. This behavior confirms that film diffusion constitutes a dominant factor in controlling the adsorption kinetics of AY36, MR, and MB dyes onto RAB-A. As depicted in Figs. 16d, 17d, and 18d, the adsorption rates of AY36 and MR increased gradually with prolonged contact time. The IPDM rate constants $\:{K}_{\mathrm{dif}}$ were calculated to fall within the ranges of 0.19–11.61 mg g^–1^ min^–1/2^ for AY36, 0.40-11.45 mg g^–1^ min^–1/2^ for MR dye, and 0.08–19.38 mg g^–1^ min⁻^1/2^ for MB dye.

#### Film diffusion model (FDM)

The film diffusion model (FDM) accounts for the migration of adsorbate species through the liquid boundary layer enveloping the adsorbent particles^57^, and this mechanism is quantitatively described by Eq. (18).

18 $$\:\mathrm{ln}\left(1-F\right)={K}_{FD}\left(t\right)$$

where represents the external film mass transfer coefficient, whereas F corresponds to the ratio of $\:{q}_{t}$ to $\:{q}_{e}$. The $\:{K}_{\mathrm{FD}}$ values were obtained from the slope and intercept of the linearized $\:\left(1-\mathrm{F}\right)$ plots, as shown in Figs. 16e, 17e, and 18e^103^. Among the kinetic models examined, the PSOM was found to most accurately describe the electrochemical adsorption behavior of all three dyes onto RAB-A. This conclusion is supported by the fact that the corresponding linear plots do not pass through the origin, indicating that film diffusion is not the primary rate-limiting step. In addition, PSOM exhibited the highest correlation coefficient (*R*^2^ = 0.999), thus affirming its appropriateness for representing the adsorption kinetics. During the initial adsorption phase, electrostatic interactions are likely to occur between the negatively charged functional groups on the self-doped activated carbon surface and hydrogen ions in the aqueous medium. This interaction pathway aligns well with the observed trends in the PSOM, LIM, and TIM isotherm models. In addition, the presence of multiple nitrogen atoms with lone electron pairs in the adsorbate molecules further promotes their interaction with the adsorbent surface, thereby enhancing adsorption affinity.

### Comparison with the result reported in the literature

A comparative analysis grounded in previously reported studies was conducted to evaluate the efficiency of various adsorbent materials for azo dye removal, with special emphasis on RAB-A. Table 2 summarizes the adsorption capacities (mg/g) obtained in the present work and contrasts them with values reported in the literature. The results demonstrate that RAB-A exhibits high removal efficiency toward AY36, MR, and MB dyes, underscoring its promise as a competitive adsorbent.

**Table 2:** Comparison of the maximum adsorption capacities of RAB-A for the removal of AY36, MR, and MB dyes.

Adsorbent	Pollutant	Q_m_ (mg/g)	Refs.
AC from peanut shells	AY36 dye	66.70	^7^
N-doping activated carbons from fish waste and sawdust	AY36 dye	232.56	^104^
Superabsorbent hydrogel of Poly(3-acrylamidopropyl)-trimethylammonium chloride-co-N, N-DMA	AY36 dye	199.96	^105^
Refused tea waste activated carbon	AY36 dye	71.97	^106^
Green nanoceria (GN) and GN–NH_2_ (AGN) from Prosopis julifora leaves extract	AY36 dye	26.95	^107^
Caraway seeds	MR dye	208.00	^108^
Fennel seeds	MR dye	135.00	^109^
Biogas Plant Waste	MR dye	113.00	^110^
Green tea leaves	MR dye	103.00	^111^
Marigold	MR dye	102.43	^112^
Orange peel	MB dye	280.00	^113^
Activated carbon from rosemary plant	MB dye	110.67	^114^
*Azolla pinnata*	MB dye	80.60	^115^
Jute stick charcoal	MB dye	29.32	^116^
Corn Stigmata	MB dye	106.30	^117^
RAB-A	AY36 dye	222.22	This Study
RAB-A	MR dye	192.31	This Study
RAB-A	MB dye	833.33	This Study

### ANN study

**Fig. 19:**
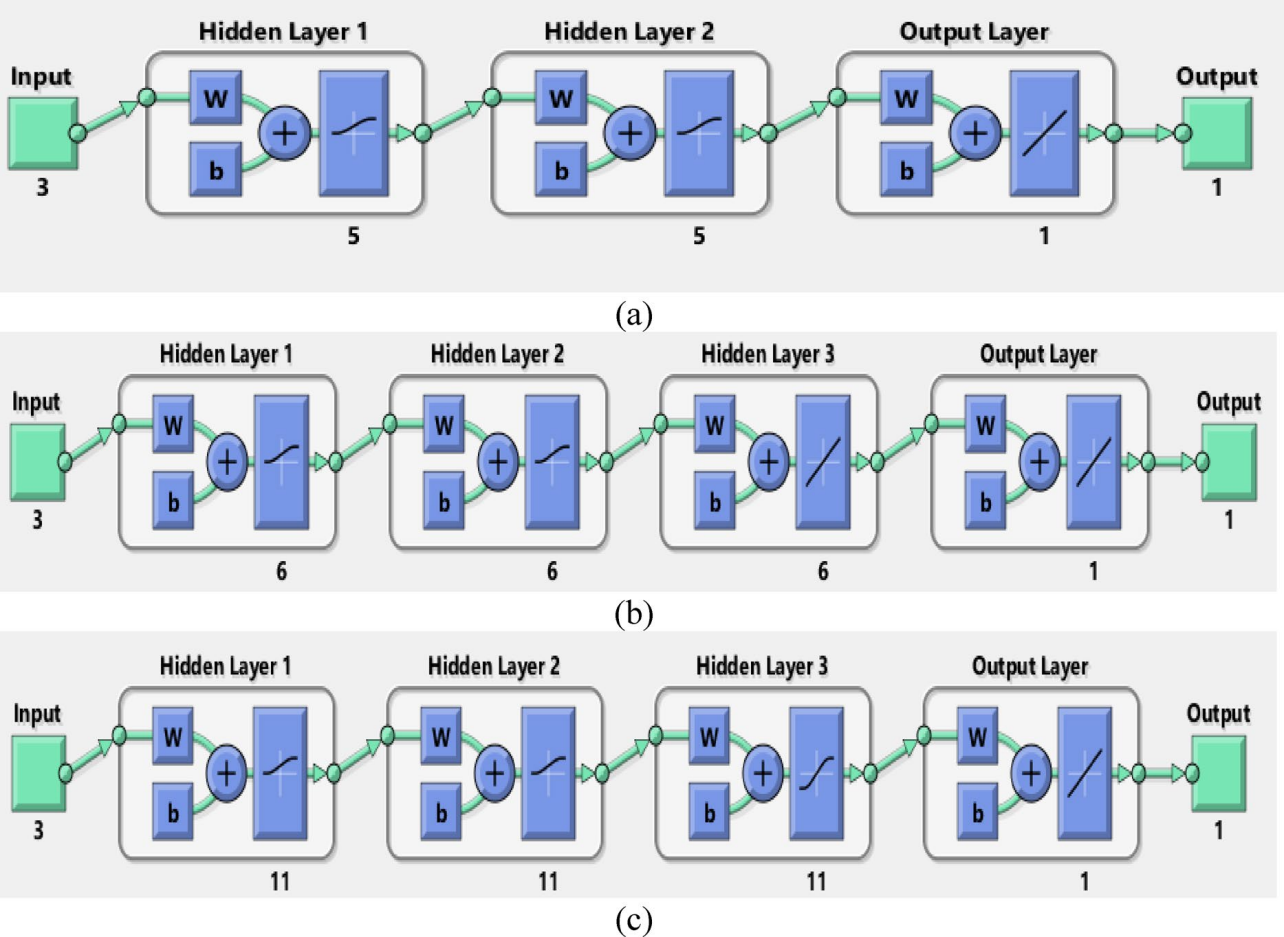
ANN architecture for the elimination of (**a**) AY36 dye, (**b**) MR dye, and (**c**) MB dye.

**Fig. 20:**
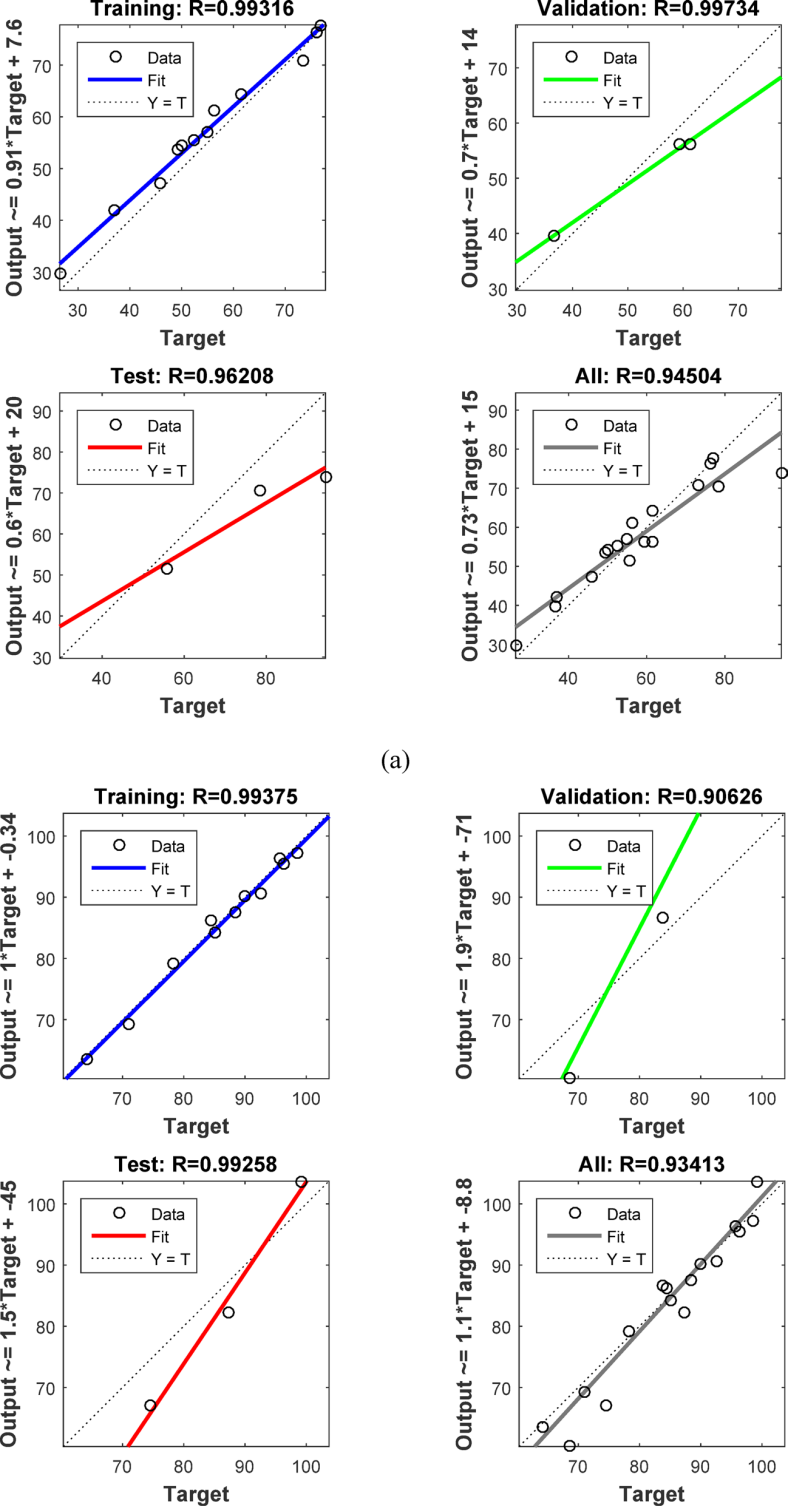

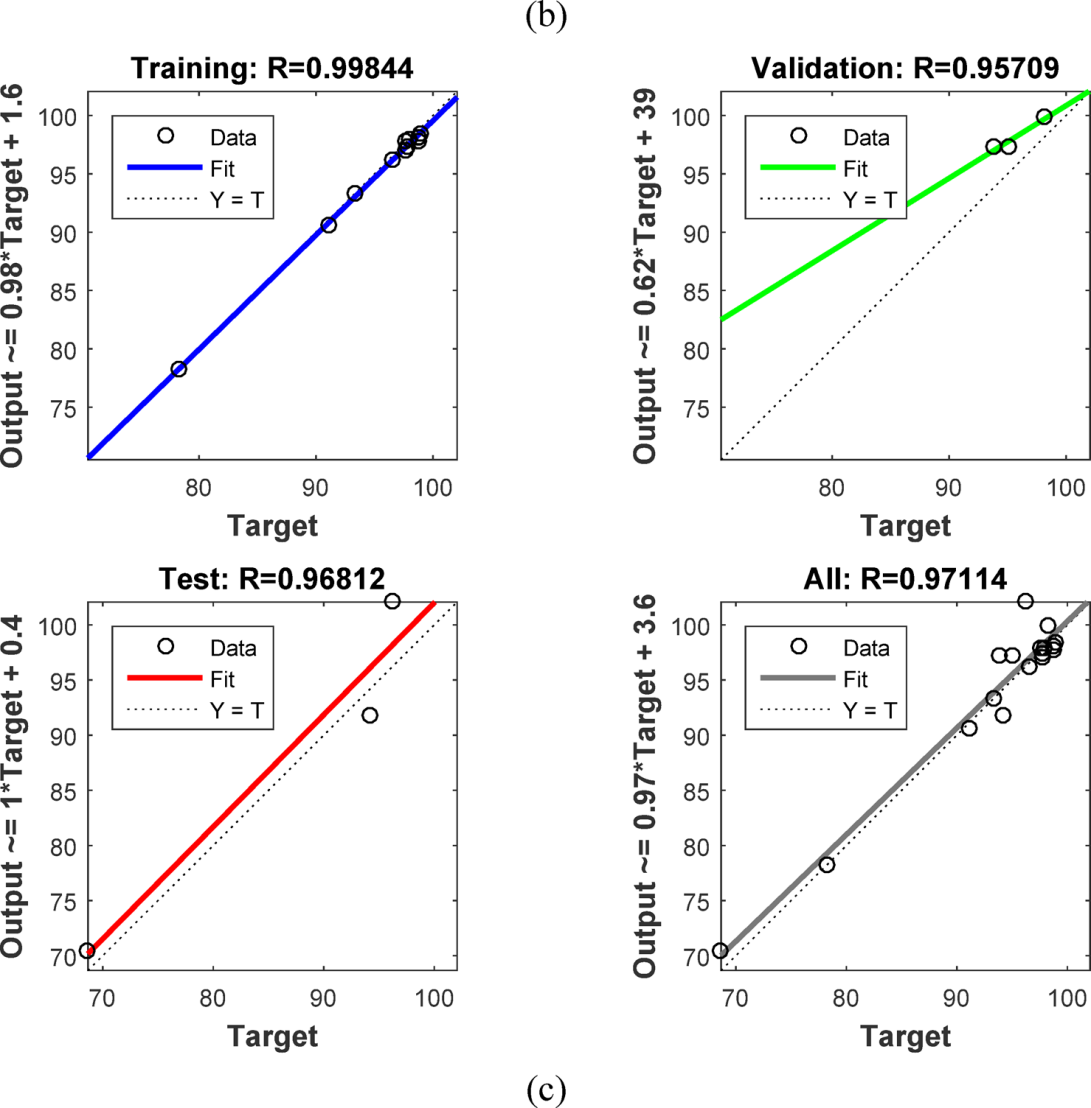
Training, validation, testing, and overall datasets for the LM algorithm of (**a**) AY36 dye, (**b**) MR dye, and (**c**) MB dye.

**Fig. 21:**
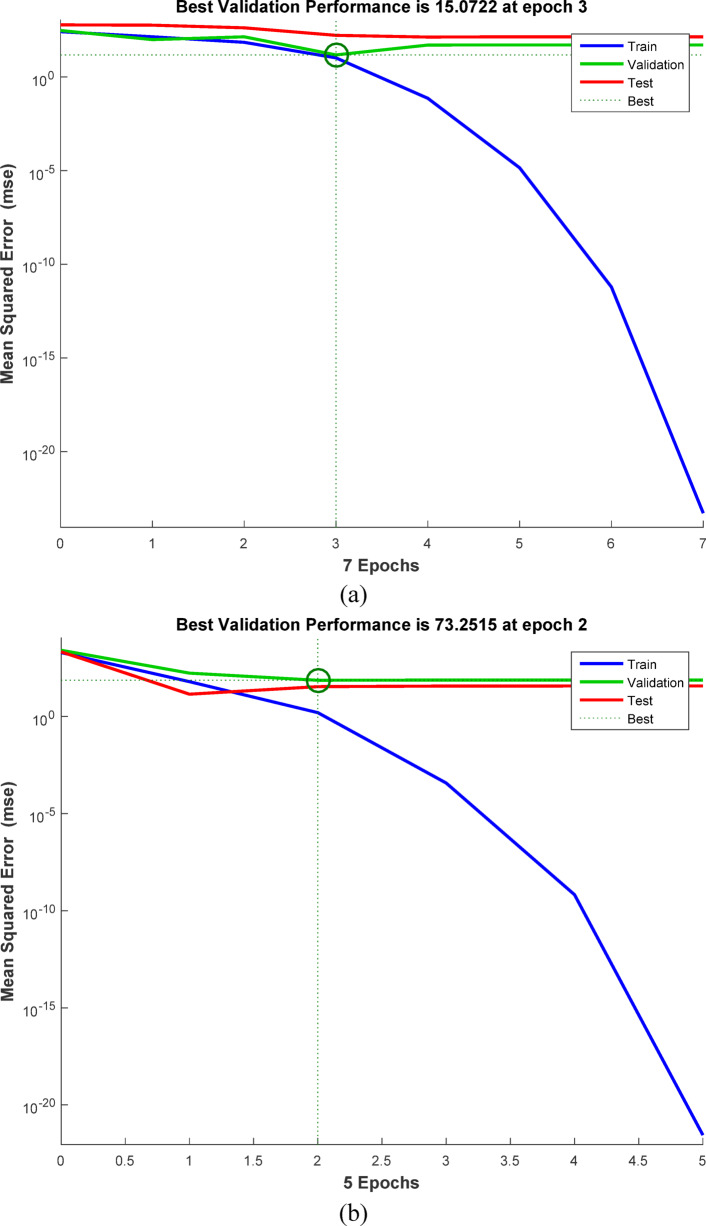

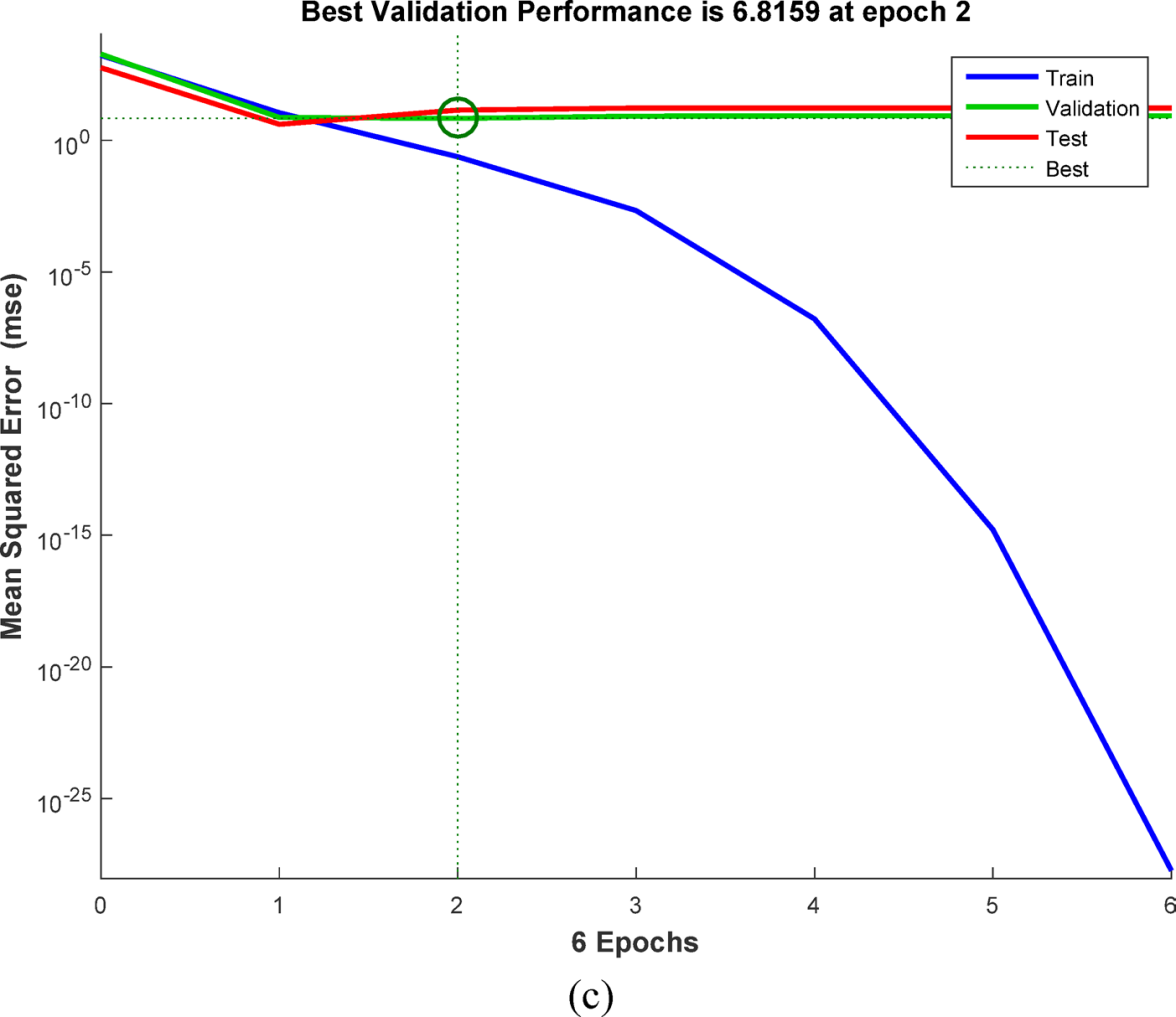
LM algorithm performance of (**a**) AY36 dye, (**b**) MR dye, and (**c**) MB dye.

The ANN model was trained on sample data (training (70%), testing (15%), and validation (15%)) using the backpropagation algorithm. To forecast the removal effectiveness of AY36 dye using RAB-N, the ANN model uses a feedforward architecture with three input layers, two hidden layers, each with five neurons, and one output layer, as shown in Fig. 19a. Three neurons (represented by the letter “3”) are used in the input layer to receive important process variables, including initial dye concentration, adsorbent dosage, and contact time. The output layer predicts the % elimination of AY36 dye using just one neuron (“1”). For nonlinear dye adsorption kinetics, this 3-5-5-1 structure strikes a balance between generalization and complexity. The regression plots are shown in Fig. 20a. *R*^2^ training was 0.99316. *R*^2^ validation and testing were 0.99734 and 0.96208, respectively. The overall model reliability is confirmed by the all-data plot’s *R*^2^ = 0.9540. The overall model reliability is confirmed by the all-data plot’s *R*^2^ = 0.9540. Outliers are likely due to exceptional circumstances, and the residuals cluster around zero. The model’s mean squared error (MSE) was 5.2 × 10^–24^. Log-sigmoid (log-sig) activation functions were employed in both hidden layers, while a pure linear (purelin) function was applied at the output layer. In the optimized ANN, the adsorbent dosage of RAB-A (g/L), contact time (min), and the initial AY36 concentration were selected as the three input parameters, whereas the percentage removal of AY36 served as the model output. Divergence shown in validation curves after epoch 4 is avoided by ending early at epoch 3. This rapid convergence highlights how well the ANN captures the kinetics (PSOM) and adsorption isotherms (e.g., LIM or FIM models) underlying AY36 elimination by RAB-A. The MSE error versus the epoch number for the optimized ANN model stopped after 3 epochs, as shown in Fig. 21a^118^.

By managing multicollinearity among factors such as RAB-A dosage and contact duration, which are crucial for improving AY36 decolorization in textile wastewater, this ANN model outperforms conventional empirical models (such as the response surface approach). Mass transfer constraints and intraparticle diffusion in RAB-A, a sustainable biosorbent derived from agricultural waste, are skillfully modeled using a two-hidden-layer design. Process simulation is enabled by *R*^2^ > 0.95 on combined data, supporting scalable water treatment by forecasting > 95% removal under ideal conditions. Future improvements could include dropout regularization or Levenberg-Marquardt optimization to increase test *R*^2^ to near 0.9, thereby improving generalizability to actual effluents.

In the ANN-based analysis, the dataset was split into training (70%), testing (15%), and validation (15%) subsets, and the network was trained using the backpropagation algorithm. The optimized ANN architecture developed for MR removal by RAB-A consisted of three neurons in the input layer, three hidden layers each containing six neurons, and a single neuron in the output layer, as depicted in Fig. 19b. Regression analysis presented in Fig. 20b revealed high predictive accuracy, with *R*² values of 0.99375 for training, 0.90628 for validation, 0.99258 for testing, and an overall *R*² of 0.93413. The corresponding mean squared error (MSE) was calculated as 2.81 × 10^–22^. Log-sigmoid (log-sig) functions were employed as activation functions in the first and second hidden layers, while purelin functions were used in the third hidden layer and the output layer. The optimized ANN used three input parameters—RAB-A adsorbent dosage (g/L), contact time (min), and initial MR concentration—and the output variable was the MR dye removal efficiency. As illustrated in Fig. 21b, the MSE of the optimized ANN model converged rapidly and stabilized after 2 training epochs^61^.

For the ANN analysis, the dataset was partitioned into training (70%), testing (15%), and validation (15%) subsets, and the network was trained using the backpropagation algorithm. The optimized ANN architecture developed for MB dye removal by RAB-A consisted of three neurons in the input layer, three hidden layers each containing eleven neurons, and a single neuron in the output layer, as illustrated in Fig. 19c. The corresponding regression plots are presented in Fig. 20c, yielding *R*^2^ values of 0.99844 for training, 0.95709 for validation, 0.96812 for testing, and an overall *R*^2^ of 0.97114. The model’s mean squared error (MSE) was 1.84 × 10^–28^. Log-sigmoid (log-sig) activation functions were applied in the first and second hidden layers, while a tan-sigmoid function was employed in the third hidden layer, and a purelin function was used for the output layer. The optimized ANN included three input parameters—RAB-A dosage (g/L), contact time (min), and initial MB concentration—and a single output variable representing MB removal efficiency. As shown in Fig. 21c, the MSE decreased rapidly with increasing epoch number, and the optimized ANN model converged after two epochs^119^.

### Mechanism of adsorption

AY36, MR, and MB’s adsorption behavior onto RAB-A can be understood as a synergistic process controlled by surface charge properties, functional groups comprising oxygen and nitrogen, aromatic carbon domains, and the adsorbent’s mesoporous nature. The pH-dependent surface chemistry of RAB-A, as reflected in its pH_PZC_ value of roughly 8.8, is a major factor in regulating this behavior. The adsorbent surface becomes protonated at pH values below pH_PZC_, creating positively charged sites that facilitate the adsorption of the anionic dyes AY36 and MR via electrostatic interactions with their negatively charged functional groups. The experimental finding that AY36 and MR achieved their maximum removal efficiencies in highly acidic environments is consistent with this explanation.

On the other hand, surface deprotonation leads to the development of negatively charged sites at pH values above pH_PZC_, which promotes adsorption of the cationic MB dye via electrostatic interactions and explains why it is best removed in alkaline media. Dye uptake is anticipated to be influenced by additional intermolecular interactions beyond electrostatic forces. Numerous surface functionalities, such as hydroxyl, carbonyl, and C–O groups, as well as nitrogen-containing species introduced by NH_4_OH modification, such as pyridinic and graphitic nitrogen, were validated by FTIR and XPS investigations. Through specific surface complexation and hydrogen bonding, these functional groups may provide additional adsorption sites.

Additionally, the aromatic structure of the carbon matrix may promote π–π interactions between the aromatic rings of AY36, MR, and MB molecules and the delocalized π-electron system of RAB-A, thereby improving adsorption affinity and stability. The suggested mechanism is further supported by the textural features of RAB-A. The material has a mesoporous structure with an average pore diameter of around 13.9 nm, which is large enough to permit the diffusion and accommodation of dye molecules within the pore network, while maintaining a relatively low BET surface area. As a result, pore filling and diffusion likely work in tandem with surface contacts, thereby enhancing overall adsorption efficacy.

**Fig. 22:**
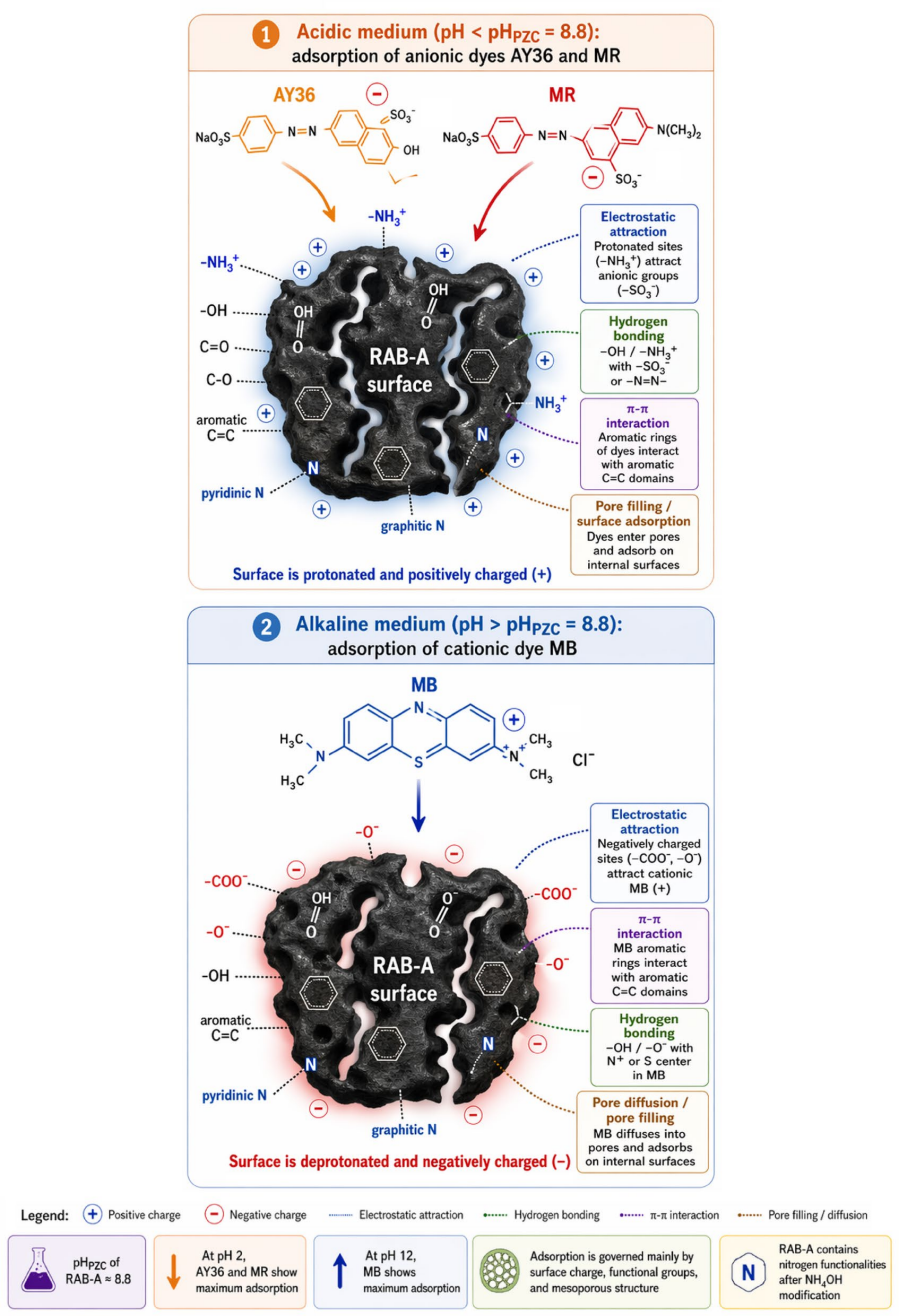
Proposed adsorption mechanism diagram of AY36, MR, and MB dyes on RAB-A surface.

This implies that the accessibility of RAB-A’s pore structure and its surface chemical functionality, rather than just its surface area, determines its adsorption efficiency. When combined, these results show that dye adsorption onto RAB-A occurs via a multi-mechanistic pathway, with hydrogen bonding, π–π interactions, and pore-filling effects contributing in complementary ways, while electrostatic attraction serves as the primary driving force. The suggested technique also emphasizes that ammonia modification can alter the surface chemistry of biochar derived from red algae, thereby enhancing its ability to adsorb both cationic and anionic dyes (Fig. 22).

The techno-economic analysis, with a qualitative/semi-quantitative focus rather than a full financial model, was added to the supplementary materials (Table S11).

### Regeneration and reusability of RAB-A adsorbent

**Fig. 23:**
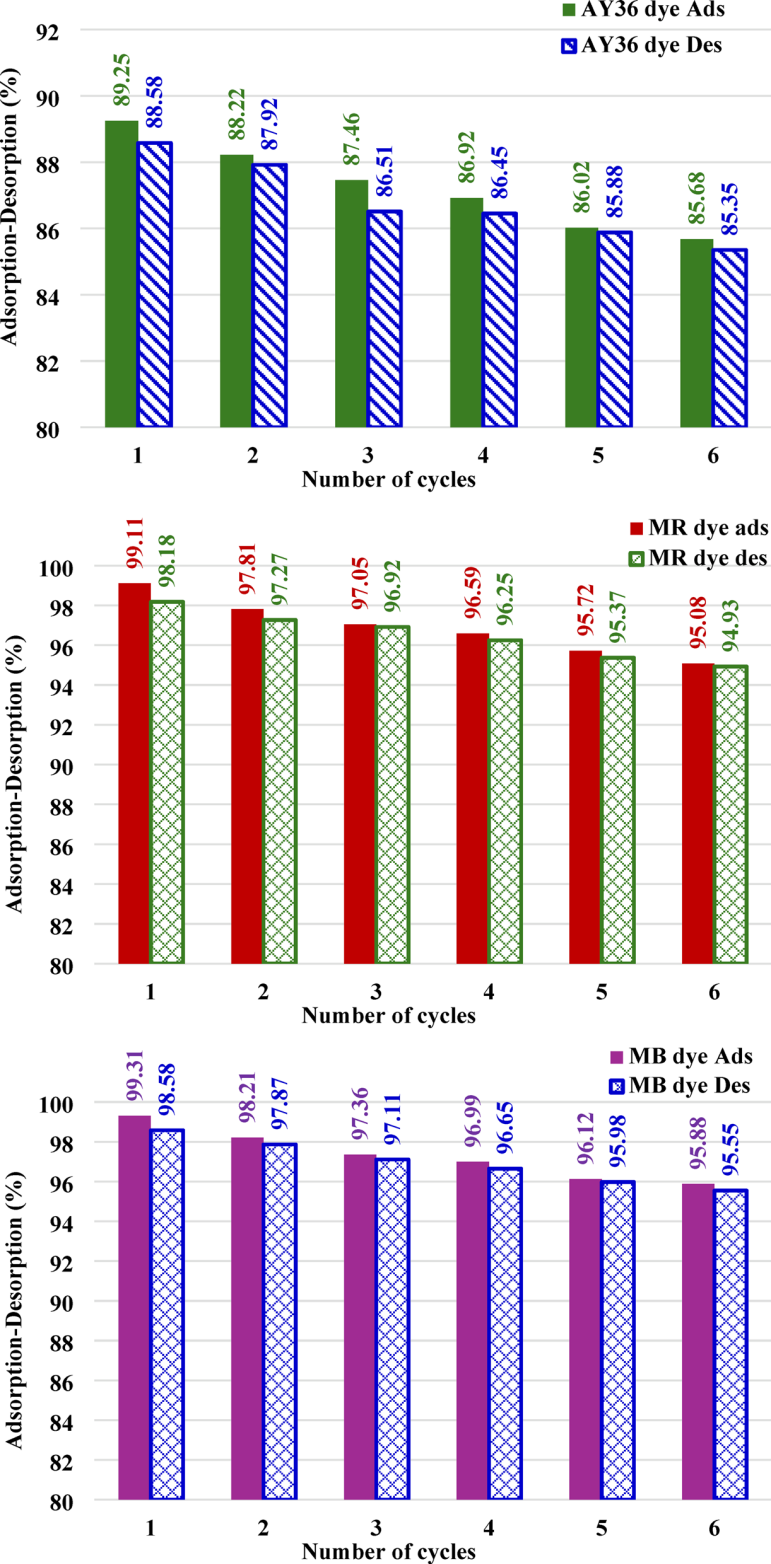
Regeneration data of RAB-A using AY36, MR, and MB dyes using 100, 200, and 200 dye concentrations, respectively, and 1.5 g/L adsorbent concentration.

The regeneration processes of the RAB-A adsorbent were conducted using 0.1 M NaOH (200 mL for 50 min) and 0.1 M HCl (200 mL for 50 min) as elution and regeneration media, respectively, to assess the reusability of RAB-A as an adsorbent. The regeneration efficiency of RAB-A was evaluated across six consecutive adsorption and desorption cycles using AY36, MR, and MB dyes at initial concentrations of 100, 200, and 200 mg/L, respectively, and RAB-A adsorbent dose of 1.5 g/L (Fig. 23). In all cases, the adsorption and desorption efficiency gradually decreased with increasing number of cycles, as expected due to the gradual occupancy of active sites, partial pore blockage, and the potential loss or alteration of surface functional groups after repeated use. However, the decrease was minimal, indicating the stability of the resulting sorbent and its high reusability.

The adsorption efficiency of AY36 decreased from 89.25% in the first cycle to 85.68% in the sixth cycle, while the removal efficiency decreased from 88.58% to 85.35%. This results in a relatively small loss of approximately 3.57% points in adsorption efficiency and 3.23% points in removal efficiency after six cycles. In the case of MR, adsorption decreased from 99.11% to 95.08%, and desorption decreased from 98.18% to 94.93%, resulting in total decreases of 4.03 and 3.25% points, respectively. Similarly, MB dye showed good stability in regeneration, with adsorption decreasing from 99.31% to 95.88% and desorption from 98.58% to 95.55% after six cycles, resulting in losses of 3.43 and 3.03% points, respectively.

These results show that RAB-A retained a significant portion of its initial performance, with dye removal efficiency exceeding 85% for AY36 and 95% for both MR and MB after the sixth cycle. The substantial improvement in regeneration behavior for MR and MB compared to AY36 is attributed to stronger, more reversible bonds between the dye molecules and the available active sites on RAB-A, as well as to the greater accessibility of the intermediate porous structure. Overall, the slight decrease in adsorption and desorption efficiency indicates that the ammonia-modified biochar derived from red algae (RAB-A) possesses good structural stability and high regeneration capacity, making it a suitable adsorbent for repeated use in the treatment of dye-contaminated wastewate^120–122^.

## Conclusion

This study produced ammonia-functionalized biochar from red algae (RAB-A) via a reflux-assisted process using 25% ammonium hydroxide (NH₄OH). This synthesis technique provides an environmentally friendly and economically viable strategy for manufacturing efficient biomass-based adsorbents while addressing challenges related to dye-polluted effluents and biomass waste valorization. The ammoniation procedure altered the biochar’s surface chemistry by introducing nitrogen-bearing functional groups, significantly increasing its adsorption affinity for both anionic dyes (such as AY36 and MR) and the cationic dye MB. This intentional surface functionalization represents a significant conceptual development in the rational design of high-efficiency adsorbent materials. Structural analyses confirmed the successful formation of activated carbon characterized by a high degree of microporosity and an enlarged specific surface area.

The adsorption performance of RAB-A toward AY36, MR, and MB dyes was systematically examined under optimized experimental conditions. To elucidate the adsorption mechanisms, an extensive set of equilibrium and kinetic models—namely the Langmuir, Freundlich, Temkin, Dubinin–Radushkevich, Halsey, Harkins–Jura, pseudo-first-order, pseudo-second-order, Elovich, intraparticle diffusion, and film diffusion models—was applied. This comprehensive modeling framework enabled a more rigorous interpretation of the interactions governing dye uptake. According to the LIM model, the maximum adsorption capacities of RAB-A were determined to be 222.22, 192.31, and 833.33 mg/g for AY36, MR, and MB, respectively. Among the tested equilibrium models, the FIM provided the best fit for MR and MB adsorption, whereas the LIM and TIM models more accurately described the adsorption behavior of AY36.

Kinetic evaluation demonstrated that the adsorption behavior of all three dyes was best described by the PSOM, indicating that chemisorption governs the rate-limiting step of the process. Overall, the ammonia-modified red algae biochar demonstrated exceptional removal efficiencies for both anionic and cationic dyes, underscoring its effectiveness for aqueous-phase remediation. In addition, ANN modeling was employed to predict and optimize the adsorption process. The combination of experimental approaches with advanced computational modeling provides a robust and innovative strategy for precise prediction of dye removal efficiencies and effective optimization of operational parameters in environmental engineering applications.

The efficient removal of AY36, MR, and MB using RAB-A underscores its strong potential as a sustainable, economical, and environmentally benign adsorbent for industrial wastewater treatment applications. Beyond its immediate application, this study contributes to broader objectives related to pollution mitigation and sustainable resource utilization. The detailed characterization and mechanistic understanding established herein provide valuable guidance for the design and development of next-generation biochar-based adsorbents with improved selectivity and adsorption capacity for a wide range of contaminants. The results also encourage continued exploration of red algae and other underutilized biomasses as precursors for advanced functional materials.

The adsorption mechanism involved electrostatic attraction, hydrogen bonding, π-π interaction, and pore-filling effects. Furthermore, the ANN model demonstrated strong predictive ability for dye removal efficiency, demonstrating its utility as a supporting optimization tool for adsorption systems. The scientific significance of this work lies in the creation of a sustainable, algae-derived adsorbent functionalized with ammonia that can effectively remove both anionic and cationic dyes using a single material. This advances our understanding of how nitrogen-containing surface groups and mesoporous biochar structures can be tuned to improve adsorption effectiveness against structurally diverse contaminants. From a practical standpoint, RAB-A’s high adsorption capacity, excellent removal efficiency, and outstanding regeneration performance indicate its promising potential for use in dye-contaminated wastewater treatment. As a result, this study makes a significant scientific contribution to the design of modified biochars and provides a potential foundation for future scale-up and real-effluent treatment studies.

## Supplementary Information

Below is the link to the electronic supplementary material.

Supplementary Material 1

## Data Availability

The datasets utilized in this investigation can be accessed for examination upon request from the corresponding author.
