# Supplementary material for: Acid Yellow 36, Methyl Red, and Methylene Blue adsorption using ammonia-modified red algae biochar: isotherm, kinetic, regeneration, and ANN studies
Source: Sci Rep. 2026 Jun 3;16:17189. doi: 10.1038/s41598-026-55117-4 (PMC13233837; doi:10.1038/s41598-026-55117-4)
Supplement: Supplementary file 1 — Supplementary Material 1 [file 41598_2026_55117_MOESM1_ESM.docx]

**Supplementary data**

**Isotherm, Kinetics, and ANN Analysis of Acid Yellow 36, Methyl Red, and Methylene Blue dyes Adsorption by** **Ammonia-decorated Red-Algae Biochar**

Mohamed A. Hassaan^1^, Murat Yılmaz^2^, Amany El Sikaily^1^, Nehad A. Elmaghraby^1^, Mohamed A. El-Nemr^3,4^, Ahmed El Nemr^1^*

^1^Environment Division, National Institute of Oceanography and Fisheries (NIOF), Kayet Bey, Elanfoushy, Alexandria, Egypt

^2^Osmaniye Korkut Ata University, Bahçe Vocational School, Department of Chemistry and Chemical Processing Technologies, Osmaniye, 80000, Türkiye

^3^Department of Chemical Engineering, Faculty of Engineering, Minia University, Minia 61519, Egypt

^4^The Higher Canal Institute of Engineering and Technology, Al Salam 1-Abu Bakr Al Siddiq Street, Suez, Egypt

Email address: [mhss95@mail.com](mailto:mhss95@mail.com) (M.A.H.); [muratyilmaz@osmaniye.edu.tr](mailto:muratyilmaz@osmaniye.edu.tr) (M.Y.); [dramany_mas@yahoo.com](mailto:dramany_mas@yahoo.com) (A.E.S.); [nehadaly96@yahoo.com](mailto:nehadaly96@yahoo.com) (N.A.E.); [mohamedelnemr1992@yahoo.com](mailto:mohamedelnemr1992@yahoo.com) (M.A.E.-N.)

*Corresponding author: [ahmedmoustafaelnemr@yahoo.com](mailto:ahmedmoustafaelnemr@yahoo.com); [ahmed.m.elnemr@gmail.com](mailto:ahmed.m.elnemr@gmail.com)

**Table S1**. Adsorption isotherm analysis of AY36 dye adsorption by RAB-A at 25 °C.

| **Model** | **Parameters** | **RAB-A (g/L)** | | | | |
| --- | --- | --- | --- | --- | --- | --- |
|  |  | **0.50** | **0.75** | **1.00** | **1.25** | **1.50** |
| LIM | *Q_m_* | 222.22 | 169.49 | 120.48 | 133.33 | 128.21 |
|  | *K_L_* x 10^3^ | 51.49 | 59.96 | 178.88 | 98.17 | 90.70 |
|  | *R*^2^ | 1.000 | 0.985 | 0.972 | 0.983 | 0.980 |
| FIM | *1/n* | 0.21 | 0.23 | 0.24 | 0.21 | 0.40 |
|  | *K_F_ (mg^1-1/n^L^1/n^g^–1^)* | 26.18 | 13.95 | 14.58 | 14.49 | 12.02 |
|  | *R*^2^ | 0.915 | 0.971 | 0.941 | 0.965 | 0.907 |
| TIM | *A_T_* | 956.17 | 12.73 | 1.08 | 4.20 | 21.41 |
|  | *B_T_* | 4.32 | 4.97 | 10.20 | 18.12 | 5.62 |
|  | *b_T_* | 573.90 | 498.92 | 242.97 | 136.73 | 441.19 |
|  | *R*^2^ | 1.000 | 0.987 | 1.000 | 0.992 | 0.995 |
| DRIM | *Q_m_ (mol kg^–1^)* | 307.60 | 228.45 | 175.14 | 144.65 | 89.83 |
|  | *K* × 10^6^ (mol kJ^–1^)^2^ | 1.11x10^-8^ | 4.9x10^-9^ | 2.8x10^-9^ | 1.9x10^-9^ | 1.2x10^-9^ |
|  | *E* (KJ mol^-1^) | 6.712 | 10.102 | 13.363 | 22.942 | 20.412 |
|  | *R*^2^ | 0.974 | 0.965 | 0.985 | 0.991 | 0.951 |
| HIM | *1/n_H_* | 0.37 | 0.43 | 0.38 | 0.29 | 0.49 |
|  | *K_H_* | 1.17x10^5^ | 1.14x10^4^ | 3.42x10^4^ | 1.59x10^6^ | 1.1x10^3^ |
|  | *R^2^* | 0.933 | 0.969 | 0.939 | 0.969 | 0.955 |
| HJIM | *A_HJ_* | 45.87 | 14.64 | 8.61 | 11.83 | 3.19 |
|  | *B_HJ_* | 2.04 | 1.67 | 1.63 | 2.02 | 1.41 |
|  | *R^2^* | 1.000 | 0.932 | 0.974 | 0.819 | 0.995 |

**Table S2**. Adsorption isotherm analysis of MR dye adsorption by RAB-A at 25 °C

| **Model** | **Parameters** | **RAB-A (g/L)** | | | | |
| --- | --- | --- | --- | --- | --- | --- |
|  |  | **0.50** | **0.75** | **1.00** | **1.25** | **1.50** |
| LIM | *Q_m_* | 192.31 | 128.21 | 99.01 | 82.64 | 68.03 |
|  | *K_L_* x 10^3^ | 0.813 | 0.830 | 1.110 | 1.391 | 1.225 |
|  | *R*^2^ | 0.962 | 0.958 | 0.964 | 0.969 | 0,978 |
| FIM | *1/n* | 5.563 | 5.150 | 4.878 | 4.676 | 4.497 |
|  | *K_F_ (mg^1-1/n^L^1/n^g^–1^)* | 1.944 | 2.759 | 3.408 | 3.946 | 4.465 |
|  | *R*^2^ | 0.999 | 0.999 | 0.999 | 0.999 | 1.000 |
| TIM | *A_T_* | 1043 | 507 | 674 | 1291 | 992 |
|  | *B_T_* | 57.34 | 44.73 | 30.31 | 20.66 | 17.77 |
|  | *R*^2^ | 0.510 | 0.700 | 0.690 | 0.558 | 0.556 |
| DRIM | *Q_m_ (mol kg^–1^)* | 217.67 | 137.55 | 103.03 | 82.76 | 71.88 |
|  | *K* × 10^6^ (mol kJ^–1^)^2^ | 0.67 | 0.79 | 0.53 | 0.44 | 0.33 |
|  | *E* (KJ mol^-1^) | 27.379 | 25.158 | 30.715 | 33.710 | 38.925 |
|  | *R*^2^ | 0.420 | 0.679 | 0.777 | 0.801 | 0.702 |
| HIM | *1/n_H_* | 0.209 | 0.243 | 0.215 | 0.178 | 0.184 |
|  | *K_H_* | 3.21x10^12^ | 1.44x10^10^ | 4.93x10^10^ | 1.39x10^12^ | 2.15x10^11^ |
|  | *R^2^* | 0.493 | 0.660 | 0.646 | 0.511 | 0.512 |
| HJIM | *A_HJ_* | 69,93 | 26,95 | 17,86 | 14,81 | 10,05 |
|  | *B_HJ_* | 0,31 | 0,16 | 0,39 | 0,77 | 0,75 |
|  | *R^2^* | 0.426 | 0.542 | 0.525 | 0.399 | 0.401 |

**Table S3**. Adsorption isotherm analysis of MB dye adsorption by RAB-A at 25 °C

| **Model** | **Parameters** | **RAB-A (g/L)** | | | | |
| --- | --- | --- | --- | --- | --- | --- |
|  |  | **0.50** | **0.75** | **1.00** | **1.25** | **1.50** |
| LIM | *Q_m_* | 303.03 | 588.24 | 625.00 | 833.33 | 76.34 |
|  | *K_L_* x 10^3^ | 8.250 | 0.270 | 0.119 | 0.096 | 0.396 |
|  | *R*^2^ | 0.922 | 0.985 | 0.992 | 0.927 | 0.925 |
| FIM | *1/n* | 1.51 | 5.90 | 0.92 | 1.71 | 0.90 |
|  | *K_F_ (mg^1-1/n^L^1/n^g^–1^)* | 82.89 | 76.82 | 18.17 | 97.52 | 57.84 |
|  | *R*^2^ | 0.971 | 0.979 | 0.977 | 0.975 | 0.974 |
| TIM | *A_T_* | 9.74 | 1.44x10^35^ | 21.08 | 775.80 | 231.19 |
|  | *B_T_* | 82.70 | 4.43 | 54.23 | 34.984 | 40.112 |
|  | *b_T_* | 29.96 | 559.71 | 45.69 | 70.82 | 61.77 |
|  | *R*^2^ | 0.970 | 0.938 | 0.906 | 0.912 | 0,987 |
| DRIM | *Q_m_ (mol kg^–1^)* | 90.30 | 19.06 | 128.02 | 8.20 | 18.34 |
|  | *K* × 10^6^ (mol kJ^–1^)^2^ | 0.00 | -0.0012 | 0.00 | -0.0041 | -0.0028 |
|  | *E* (KJ mol^-1^) | -1000.00 | -20.41 | 13.61 | -11.04 | -13.36 |
|  | *R*^2^ | 0.982 | 0.980 | 0.856 | 0.973 | 0.960 |
| HIM | *1/n_H_* | 1.45 | 5.90 | 0.92 | 1.71 | 0.91 |
|  | *K_H_* | 21.66 | 1.63 | 112.58 | 10.72 | 145.22 |
|  | *R^2^* | 0.983 | 0.979 | 0.977 | 0.973 | 0.982 |
| HJIM | *A_HJ_* | 2.10 | 0.47 | 2.11 | 1.85 | 14.88 |
|  | *B_HJ_* | 0.28 | 0.18 | 0.36 | 0.32 | 0.74 |
|  | *R^2^* | 0.921 | 0.923 | 0.913 | 0.999 | 0.926 |

**Table S4.** Error function values of isotherm models applied to the equilibrium adsorption of AY36, MR, and MB dyes on RAB-A.

| **Dye** | **Isotherm Model** | **APE (%)** | **X^2^** | **Hybrid** | **ERRSQ** | **MPSD** | **ARE** | **EABS** | **RMS** |
| --- | --- | --- | --- | --- | --- | --- | --- | --- | --- |
| AY36 | LIM | 0.013 | 0.030 | 0.131 | 3.322 | 0.07 | 0.013 | 9.11 | 0.07 |
|  | FIM | 0.002 | 0.000 | 0.001 | 0.031 | 0.01 | 0.002 | 0.89 | 0.01 |
|  | TIM | 0.012 | 0.023 | 0.098 | 2.244 | 0.06 | 0.012 | 7.49 | 0.06 |
|  | DRIM | 0.010 | 0.024 | 0.105 | 4.160 | 0.05 | 0.010 | 10.20 | 0.05 |
|  | HIM | 0.014 | 0.030 | 0.131 | 2.861 | 0.07 | 0.014 | 8.458 | 0.07 |
|  | HJIM | 0.076 | 1.53 | 6.668 | 263.079 | 0.39 | 0.076 | 81.10 | 0.38 |
| MR | LIM | 1.582 | 618.58 | 2689.48 | 97815 | 8.25 | 1.582 | 1563.77 | 7.91 |
|  | FIM | 4.00 | 516.15 | 2244.13 | 10656 | 20.85 | 4.000 | 516.15 | 20.00 |
|  | TIM | 4.00 | 3544.61 | 15411 | 502571.57 | 20.85 | 4.000 | 3544.61 | 20.00 |
|  | DRIM | 1.40 | 483.8 | 2103.4 | 76500.9 | 7.29 | 1.399 | 1382.9 | 7.00 |
|  | HIM | 5.51 | 7497.3 | 32596.9 | 1185536 | 28.72 | 5.509 | 5444.1 | 27.54 |
| MB | LIM | 0.78 | 123.07 | 535.07 | 23716.512 | 4.04 | 1.140 | 634.97 | 4.70 |
|  | FIM | 5.09 | 1805.56 | 11284.75 | 215092.13 | 22.94 | 5.09 | 1967.65 | 21.62 |
|  | TIM | 1.83 | 685.44 | 2980.17 | 131522.8 | 9.01 | 1.832 | 1701.03 | 8.59 |
|  | DRIM | 3.490 | 1175.14 | 7344.62 | 194457.395 | 15.70 | 3.490 | 1870.89 | 14.81 |
|  | HIM | 23.46 | 15311.76 | 90069.16 | 620859.50 | 108.13 | 17.83 | 3434.58 | 102.28 |
|  | HJIM | 0.18 | 3.16 | 19.72 | 523.33 | 0.81 | 0.18 | 97.06 | 0.77 |

**Table S5**. Estimated vs. Observed *q_e_* values and PFOM/PSOM adsorption rate constants for different AY36 dye and RAB-A concentrations

| **Parameter** | | |  | **PFOM** | | |  | **PSOM** | | | |
| --- | --- | --- | --- | --- | --- | --- | --- | --- | --- | --- | --- |
| **RAB-A**  **(g/L)** | **AY36 dye**  **(mg/L)** | ***q*_e_ (exp.)** |  | ***q*_e_ (calc.)** | ***k*_1_ × 10^3^** | ***R*^2^** |  | ***q*_e_**  **(calc.)** | ***k*_2_ × 10^3^** | ***h* × 10^3^** | ***R*^2^** |
| 0.50 | 100 | 178.22 |  | 93.39 | 42.38 | 0.968 |  | 185.19 | 1.05 | 3.6x10^4^ | 1.000 |
|  | 125 | 223.56 |  | 71.73 | 23.26 | 0.992 |  | 227.27 | 0.86 | 4.46x10^4^ | 0.999 |
|  | 150 | 256.16 |  | 111.25 | 25.33 | 0.990 |  | 263.16 | 0.55 | 3.83x10^4^ | 0.998 |
|  | 175 | 288.49 |  | 191.78 | 26.95 | 0.953 |  | 294.12 | 0.33 | 2.83x10^4^ | 0.995 |
|  | 200 | 304.11 |  | 449.47 | 65.87 | 0.919 |  | 312.50 | 0.37 | 3.65x10^4^ | 0.998 |
| 0.75 | 100 | 124.84 |  | 10.57 | 26.71 | 0.969 |  | 126.58 | 12.48 | 2.00x10^5^ | 1.000 |
|  | 125 | 154.70 |  | 25.27 | 17.73 | 0.984 |  | 156.25 | 2.38 | 5.81x10^4^ | 1.000 |
|  | 150 | 176.71 |  | 32.05 | 20.50 | 0.956 |  | 178.57 | 1.96 | 6.25x10^4^ | 1.000 |
|  | 175 | 206.58 |  | 151.25 | 52.05 | 0.921 |  | 212.77 | 1.03 | 4.65x10^4^ | 1.000 |
|  | 200 | 231.96 |  | 69.23 | 29.25 | 0.905 |  | 238.10 | 1.03 | 5.85x10^4^ | 1.000 |
| 1.00 | 100 | 95.21 |  | 14.85 | 40.07 | 0.952 |  | 96.15 | 8.45 | 7.81x10^4^ | 1.000 |
|  | 125 | 92.04 |  | 25.96 | 49.74 | 0.934 |  | 88.50 | 11.30 | 8.85x10^4^ | 1.000 |
|  | 150 | 134.49 |  | 47.99 | 55.73 | 0.914 |  | 135.14 | 4.42 | 8.06x10^4^ | 1.000 |
|  | 175 | 158.49 |  | 22.37 | 23.03 | 0.893 |  | 161.29 | 3.31 | 8.62x10^4^ | 1.000 |
|  | 200 | 176.71 |  | 30.13 | 23.26 | 0.916 |  | 178.57 | 2.24 | 7.14x10^4^ | 1.000 |
| 1.25 | 100 | 95.21 |  | 4.37 | 8.29 | 0.984 |  | 76.34 | 40.86 | 2.38x10^5^ | 1.000 |
|  | 125 | 92.77 |  | 8.19 | 6.25 | 0.932 |  | 92.59 | 9.04 | 7.75x10^4^ | 1.000 |
|  | 150 | 107.95 |  | 22.02 | 11.60 | 0.984 |  | 108.70 | 13.65 | 1.61x10^5^ | 1.000 |
|  | 175 | 130.69 |  | 17.77 | 15.02 | 0.912 |  | 131.58 | 6.02 | 1.04x10^5^ | 1.000 |
|  | 200 | 143.56 |  | 17.04 | 15.61 | 0.900 |  | 144.93 | 7.94 | 1.67x10^5^ | 1.000 |
| 1.50 | 100 | 63.93 |  | 4.37 | 8.29 | 0.984 |  | 64.10 | 44.25 | 1.82x10^5^ | 1.000 |
|  | 125 | 77.95 |  | 6.25 | 11.75 | 0.932 |  | 78.13 | 106.39 | 6.49x10^5^ | 1.000 |
|  | 150 | 90.00 |  | 11.60 | 49.28 | 0.984 |  | 90.09 | 24.64 | 2.00x10^5^ | 1.000 |
|  | 175 | 107.85 |  | 15.02 | 27.18 | 0.912 |  | 107.53 | 26.21 | 3.03x10^5^ | 1.000 |
|  | 200 | 123.15 |  | 15.61 | 40.99 | 0.900 |  | 121.95 | 10.04 | 1.49x10^5^ | 1.000 |

**Table S6**. Adsorption rate constants of EM, IPDM, and FDM models at varying initial concentrations of AY36 dye and RAB-A.

| **RAB-A**  **(g/L)** | **AY36 dye**  **(mg/L)** |  | **EM** | | |  | **IPDM** | | |  | **FDM** | | |
| --- | --- | --- | --- | --- | --- | --- | --- | --- | --- | --- | --- | --- | --- |
|  |  |  | ***β*** | ***α*** | ***R*^2^** |  | ***K*_dif_** | ***C*** | ***R*^2^** |  | ***K*_FD_** | ***C*** | ***R*^2^** |
| 0.50 | 100 |  | 0.04 | 2.32x10^2^ | 0.974 |  | 10.64 | 86.06 | 0.905 |  | 0.04 | 0.66 | 0.968 |
|  | 125 |  | 0.05 | 1.90x10^4^ | 0.975 |  | 5.61 | 155.32 | 0.941 |  | 0.02 | 1.16 | 0.987 |
|  | 150 |  | 0.04 | 1.72x10^3^ | 0.984 |  | 8.30 | 155.03 | 0.933 |  | 0.02 | 1.09 | 0.933 |
|  | 175 |  | 0.026 | 3.11x10^2^ | 0.975 |  | 11.61 | 137.10 | 0.931 |  | 0.01 | 0.86 | 0.934 |
|  | 200 |  | 0.03 | 8.73x10^2^ | 0.974 |  | 11.19 | 168.05 | 0.962 |  | 0.07 | 0.39 | 0.919 |
| 0.75 | 100 |  | 0.30 | 6.86x10^14^ | 0.950 |  | 1.09 | 114.74 | 0.929 |  | 0.04 | 0.97 | 0.957 |
|  | 125 |  | 0.13 | 2.62x10^7^ | 0.979 |  | 1.86 | 130.77 | 0.969 |  | 0.03 | 1.07 | 0.973 |
|  | 150 |  | 0.09 | 2.95x10^5^ | 0.974 |  | 3.14 | 140.66 | 0.916 |  | 0.03 | 1.41 | 0.926 |
|  | 175 |  | 0.05 | 3.89x10^3^ | 0.968 |  | 3.65 | 161.78 | 0.937 |  | 0.02 | 1.82 | 0.974 |
|  | 200 |  | 0.05 | 7.88x10^3^ | 0.969 |  | 4.33 | 179.81 | 0.940 |  | 0.03 | 2.48 | 0.969 |
| 1.00 | 100 |  | 0.27 | 3.89x10^9^ | 0.903 |  | 1.34 | 80.46 | 0.909 |  | 0.03 | 2.25 | 0.885 |
|  | 125 |  | 0.23 | 1.13x10^10^ | 0.904 |  | 1.28 | 98.69 | 0.904 |  | 0.02 | 2.45 | 0.822 |
|  | 150 |  | 0.16 | 1.62x10^8^ | 0.952 |  | 1.85 | 112.04 | 0.917 |  | 0.04 | 1.77 | 0.926 |
|  | 175 |  | 0.13 | 8.23x10^7^ | 0.942 |  | 2.29 | 131.20 | 0.930 |  | 0.03 | 1.86 | 0.953 |
|  | 200 |  | 0.01 | 3.20x10^2^ | 0.936 |  | 2.34 | 149.90 | 0.920 |  | 0.03 | 1.53 | 0.977 |
| 1.25 | 100 |  | 0.56 | 1.13x10^17^ | 0.916 |  | 1.08 | 69.31 | 0.944 |  | 0.02 | 2.51 | 0.921 |
|  | 125 |  | 0.51 | 3.03x10^18^ | 0.975 |  | 0.58 | 85.02 | 0.910 |  | 0.03 | 2.26 | 0.952 |
|  | 150 |  | 0.35 | 7.25x10^14^ | 0.915 |  | 1.32 | 97.71 | 0.970 |  | 0.05 | 2.26 | 0.971 |
|  | 175 |  | 0.21 | 2.28x10^10^ | 0.918 |  | 0.72 | 121.73 | 0.925 |  | 0.01 | 2.70 | 0.932 |
|  | 200 |  | 0.31 | 3.54x10^17^ | 0.914 |  | 0.50 | 137.07 | 0.932 |  | 0.08 | 2.36 | 0.985 |
| 1.50 | 100 |  | 1.80 | 5.00x10^47^ | 0.921 |  | 0.20 | 61.87 | 0.884 |  | 0.03 | 3.45 | 0.916 |
|  | 125 |  | 0.55 | 4.94x10^16^ | 0.944 |  | 0.45 | 72.18 | 0.940 |  | 0.02 | 2.48 | 0.949 |
|  | 150 |  | 0.83 | 3.78x10^30^ | 0.989 |  | 0.19 | 87.55 | 0.932 |  | 0.02 | 3.36 | 0.930 |
|  | 175 |  | 1.15 | 3.26x10^51^ | 0.998 |  | 0.84 | 101.20 | 0.959 |  | 0.06 | 2.81 | 0.979 |
|  | 200 |  | 0.46 | 2.27x10^22^ | 0.997 |  | 0.58 | 115.45 | 0.900 |  | 0.01 | 2.78 | 0.906 |

**Table S7**. Comparison of predicted and experimental qₑ values for MR dye at varying initial concentrations and RAB-A dosages, along with PFOM and PSOM rate constants

| **Parameter** | | |  | **PFOM** | | |  | **PSOM** | | |
| --- | --- | --- | --- | --- | --- | --- | --- | --- | --- | --- |
| **RAB-A**  **(g/L)** | **MR dye**  **(mg/L)** | ***q*_e_ (exp.)** |  | ***q*_e_ (calc.)** | ***k*_1_ × 10^3^** | ***R*^2^** |  | ***q*_e_**  **(calc.)** | ***k*_2_ × 10^3^** | ***R*^2^** |
| 0.50 | 100 | 185,10 |  | 49,93 | 6,68 | 0,909 |  | 181,82 | 1,20 | 0,997 |
|  | 125 | 180,12 |  | 146,99 | 4,15 | 0,881 |  | 181,82 | 0,36 | 0,959 |
|  | 150 | 236,96 |  | 134,77 | 4,61 | 0,734 |  | 232,56 | 0,48 | 0,957 |
|  | 175 | 345,40 |  | 95,61 | 52,51 | 0,916 |  | 344,83 | 2,00 | 1,000 |
|  | 200 | 390,41 |  | 196,56 | 35,70 | 0,957 |  | 400,00 | 0,43 | 0,999 |
| 0.75 | 100 | 118,55 |  | 31,35 | 5,30 | 0,884 |  | 119,05 | 2,54 | 0,999 |
|  | 125 | 123,14 |  | 88,70 | 4,15 | 0,860 |  | 123,46 | 0,72 | 0,981 |
|  | 150 | 168,85 |  | 66,73 | 4,61 | 0,933 |  | 200,00 | 0,75 | 0,996 |
|  | 175 | 230,35 |  | 37,23 | 41,68 | 0,960 |  | 232,56 | 30,82 | 1,000 |
|  | 200 | 262,69 |  | 125,98 | 41,68 | 0,962 |  | 270,27 | 0,78 | 0,999 |
| 1.00 | 100 | 91,46 |  | 17,75 | 5,30 | 0,906 |  | 91,74 | 4,71 | 0,999 |
|  | 125 | 94,79 |  | 63,33 | 4,61 | 0,952 |  | 96,15 | 1,00 | 0,987 |
|  | 150 | 128,75 |  | 52,22 | 5,76 | 0,908 |  | 129,87 | 1,31 | 0,996 |
|  | 175 | 173,17 |  | 10,73 | 37,31 | 0,838 |  | 172,41 | 9,34 | 1,000 |
|  | 200 | 197,96 |  | 65,77 | 57,34 | 0,966 |  | 200,00 | 2,31 | 1,000 |
| 1.25 | 100 | 76,33 |  | 10,34 | 7,60 | 0,904 |  | 76,34 | 75,60 | 0,999 |
|  | 125 | 78,07 |  | 49,22 | 5,07 | 0,944 |  | 80,00 | 86,81 | 0,994 |
|  | 150 | 103,56 |  | 42,35 | 6,22 | 0,956 |  | 105,26 | 21,49 | 0,998 |
|  | 175 | 138,54 |  | 8,11 | 61,72 | 0,978 |  | 138,89 | 20,74 | 1,000 |
|  | 200 | 158,70 |  | 9,39 | 44,22 | 0,890 |  | 158,73 | 10,44 | 1,000 |
| 1.50 | 100 | 63,75 |  | 7,49 | 6,91 | 0,913 |  | 64,10 | 104,00 | 0,999 |
|  | 125 | 67,20 |  | 33,97 | 4,61 | 0,916 |  | 68,03 | 2,08 | 0,994 |
|  | 150 | 91,39 |  | 21,32 | 5,53 | 0,918 |  | 91,74 | 3,35 | 0,998 |
|  | 175 | 115,71 |  | 6,05 | 53,43 | 0,954 |  | 116,28 | 32,16 | 1,000 |
|  | 200 | 132,28 |  | 9,51 | 53,43 | 0,786 |  | 133,33 | 14,42 | 1,000 |

**Table S8**. Comparison of kinetic parameters (EM, IPDM, FDM) for MR dye adsorption on RAB-A under varying starting concentrations

| **RAB-A**  **(g/L)** | **MR dye**  **(mg/L)** |  | **EM** | | |  | **IPDM** | | |  | **FDM** | | |
| --- | --- | --- | --- | --- | --- | --- | --- | --- | --- | --- | --- | --- | --- |
|  |  |  | ***β*** | ***α*** | ***R*^2^** |  | ***K*_dif_** | ***C*** | ***R*^2^** |  | ***K*_FD_** | ***C*** | ***R*^2^** |
| 0.50 | 100 |  | 0,113 | 2,05x10^7^ | 0,862 |  | 2,880 | 141,31 | 0,955 |  | 0,005 | 1,433 | 0,965 |
|  | 125 |  | 0,053 | 4,9x10^2^ | 0,731 |  | 6,390 | 84,99 | 0,864 |  | 0,004 | 0,526 | 0,806 |
|  | 150 |  | 0,051 | 8,11x10^3^ | 0,642 |  | 6,300 | 142,57 | 0,708 |  | 0,004 | 0,788 | 0,594 |
|  | 175 |  | 0,054 | 1,87x10^7^ | 0,876 |  | 5,110 | 289,70 | 0,687 |  | 0,041 | 1,750 | 0,963 |
|  | 200 |  | 0,027 | 7,74x10^3^ | 0,967 |  | 11,450 | 255,07 | 0,939 |  | 0,036 | 0,686 | 0,957 |
| 0.75 | 100 |  | 0,175 | 2,38x10^7^ | 0,951 |  | 1,760 | 95,50 | 0,941 |  | 0,005 | 1,435 | 0,884 |
|  | 125 |  | 0,078 | 5,36x10^2^ | 0,861 |  | 4,110 | 65,38 | 0,918 |  | 0,004 | 0,621 | 0,860 |
|  | 150 |  | 0,097 | 3,93x10^5^ | 0,874 |  | 3,340 | 122,29 | 0,959 |  | 0,005 | 1,085 | 0,933 |
|  | 175 |  | 0,081 | 1,63x10^7^ | 0,881 |  | 3,360 | 193,99 | 0,687 |  | 0,042 | 1,823 | 0,960 |
|  | 200 |  | 0,042 | 5,29x10^-2^ | 0,948 |  | 7,110 | 180,65 | 0,890 |  | 0,042 | 0,735 | 0,962 |
| 1.00 | 100 |  | 0,286 | 3,97x10^9^ | 0,957 |  | 1,070 | 77,90 | 0,936 |  | 0,006 | 1,700 | 0,884 |
|  | 125 |  | 0,101 | 4,81x10^2^ | 0,908 |  | 3,170 | 51,93 | 0,966 |  | 0,005 | 0,663 | 0,930 |
|  | 150 |  | 0,094 | 8,09x10^3^ | 0,955 |  | 3,250 | 86,51 | 0,922 |  | 0,006 | 1,035 | 0,868 |
|  | 175 |  | 0,232 | 1,11x10^16^ | 0,549 |  | 1,130 | 160,79 | 0,359 |  | 0,029 | 3,128 | 0,770 |
|  | 200 |  | 0,074 | 2,62x10^5^ | 0,879 |  | 3,840 | 156,12 | 0,734 |  | 0,053 | 1,280 | 0,953 |
| 1.25 | 100 |  | 0,368 | 2,52x10^10^ | 0,979 |  | 0,800 | 66,47 | 0,887 |  | 0,008 | 2,015 | 0,880 |
|  | 125 |  | 0,110 | 2,12x10^2^ | 0,960 |  | 2,820 | 41,51 | 0,967 |  | 0,005 | 0,684 | 0,928 |
|  | 150 |  | 0,110 | 4,11x10^3^ | 0,985 |  | 2,790 | 68,60 | 0,967 |  | 0,007 | 1,008 | 0,957 |
|  | 175 |  | 0,381 | 2,37x10^21^ | 0,777 |  | 0,690 | 131,28 | 0,560 |  | 0,068 | 2,598 | 0,991 |
|  | 200 |  | 0,162 | 9,93x10^9^ | 0,602 |  | 1,560 | 142,47 | 0,401 |  | 0,044 | 2,827 | 0,890 |
| 1.50 | 100 |  | 0,543 | 1,09x10^13^ | 0,986 |  | 0,550 | 57,00 | 0,907 |  | 0,008 | 2,155 | 0,897 |
|  | 125 |  | 0,180 | 3,74x10^3^ | 0,901 |  | 1,740 | 43,79 | 0,933 |  | 0,005 | 0,882 | 0,878 |
|  | 150 |  | 0,244 | 7,39x10^7^ | 0,938 |  | 1,250 | 74,37 | 0,922 |  | 0,006 | 1,541 | 0,868 |
|  | 175 |  | 0,660 | 2,23x10^31^ | 0,787 |  | 0,400 | 111,42 | 0,577 |  | 0,046 | 3,233 | 0,957 |
|  | 200 |  | 0,247 | 7,18x10^12^ | 0,732 |  | 1,050 | 121,28 | 0,513 |  | 0,052 | 2,735 | 0,674 |

**Table S9**. Comparison of predicted and experimental qₑ values for MB dye at varying initial concentrations and RAB-A dosages, along with PFOM and PSOM rate constants

| **Parameter** | | |  | **PFOM** | | |  | **PSOM** | | | |
| --- | --- | --- | --- | --- | --- | --- | --- | --- | --- | --- | --- |
| **RAB-A**  **(g/L)** | **MB dye**  **(mg/L)** | ***q*_e_ (exp.)** |  | ***q*_e_ (calc.)** | ***k*_1_ × 10^3^** | ***R*^2^** |  | ***q*_e_**  **(calc.)** | ***k*_2_ × 10^3^** | ***h*** | ***R*^2^** |
| 0.50 | 100 | 196.43 |  | 59.48 | 37.08 | 0.983 |  | 200.00 | 1.59 | 63.69 | 0.999 |
|  | 125 | 131.60 |  | 125.03 | 72.31 | 0.987 |  | 250.00 | 1.60 | 100.00 | 1.000 |
|  | 150 | 98.84 |  | 155.42 | 31.09 | 0.987 |  | 303.03 | 0.43 | 39.84 | 0.998 |
|  | 175 | 79.25 |  | 118.22 | 46.52 | 0.958 |  | 357.14 | 0.89 | 113.64 | 1.000 |
|  | 200 | 99.25 |  | 159.11 | 29.25 | 0.984 |  | 400.00 | 0.43 | 68.49 | 0.999 |
| 0.75 | 100 | 246.01 |  | 9.69 | 51.82 | 0.976 |  | 131.58 | 16.04 | 277.78 | 1.000 |
|  | 125 | 164.73 |  | 10.96 | 37.77 | 0.912 |  | 163.93 | 12.40 | 333.33 | 1.000 |
|  | 150 | 123.01 |  | 47.56 | 34.08 | 0.933 |  | 200.00 | 1.70 | 68.03 | 1.000 |
|  | 175 | 98.95 |  | 63.84 | 37.08 | 0.963 |  | 232.56 | 1.44 | 78.13 | 0.999 |
|  | 200 | 82.47 |  | 125.98 | 41.68 | 0.962 |  | 270.27 | 0.78 | 56.82 | 0.999 |
| 1.00 | 100 | 291.27 |  | 3.22 | 43.53 | 0.958 |  | 99.01 | 42.50 | 416.67 | 1.000 |
|  | 125 | 197.53 |  | 7.01 | 40.99 | 0.926 |  | 123.46 | 17.73 | 270.27 | 1.000 |
|  | 150 | 148.13 |  | 13.70 | 40.07 | 0.915 |  | 149.25 | 7.13 | 158.73 | 1.000 |
|  | 175 | 118.75 |  | 26.94 | 33.39 | 0.939 |  | 175.44 | 3.46 | 106.38 | 1.000 |
|  | 200 | 99.03 |  | 65.77 | 57.34 | 0.966 |  | 200.00 | 2.31 | 92.59 | 1.000 |
| 1.25 | 100 | 345.40 |  | 1.34 | 30.63 | 0.970 |  | 79.37 | 72.16 | 454.55 | 1.000 |
|  | 125 | 230.29 |  | 2.65 | 44.22 | 0.939 |  | 99.01 | 37.78 | 370.37 | 1.000 |
|  | 150 | 173.17 |  | 12.36 | 67.71 | 0.904 |  | 119.05 | 16.80 | 238.10 | 1.000 |
|  | 175 | 138.84 |  | 16.83 | 54.81 | 0.934 |  | 138.89 | 8.79 | 169.49 | 1.000 |
|  | 200 | 115.64 |  | 12.76 | 53.43 | 0.920 |  | 158.73 | 10.44 | 263.18 | 1.000 |
| 1.50 | 100 | 390.41 |  | 0.86 | 15.20 | 0.968 |  | 66.23 | 73.55 | 322.58 | 1.000 |
|  | 125 | 262.69 |  | 1.35 | 25.10 | 0.907 |  | 82.64 | 732.05 | 5000.00 | 1.000 |
|  | 150 | 197.96 |  | 3.95 | 48.82 | 0.960 |  | 99.01 | 44.35 | 434.78 | 1.000 |
|  | 175 | 158.703 |  | 4.54 | 46.06 | 0.923 |  | 116.28 | 35.22 | 476.19 | 1.000 |
|  | 200 | 132.27 |  | 12.09 | 58.04 | 0.987 |  | 133.33 | 14.42 | 256.41 | 1.000 |

**Table S10**. Comparison of kinetic parameters (EM, IPDM, FDM) for MB dye adsorption on RAB-A under varying starting concentrations

| **RAB-A**  **(g/L)** | **MB dye**  **(mg/L)** |  | **EM** | | |  | **IPDM** | | |  | **FDM** | | |
| --- | --- | --- | --- | --- | --- | --- | --- | --- | --- | --- | --- | --- | --- |
|  |  |  | ***β*** | ***α*** | ***R*^2^** |  | ***K*_dif_** | ***C*** | ***R*^2^** |  | ***K*_FD_** | ***C*** | ***R*^2^** |
| 0.50 | 100 |  | 0.05 | 4973.19 | 0.939 |  | 5.46 | 132.06 | 0.904 |  | 0.04 | 1.19 | 0.983 |
|  | 125 |  | 0.03 | 1092.59 | 0.907 |  | 19.38 | 121.88 | 0.892 |  | 0.06 | 0.94 | 0.973 |
|  | 150 |  | 0.03 | 731.35 | 0.981 |  | 10.90 | 163.86 | 0.920 |  | 0.03 | 0.63 | 0.987 |
|  | 175 |  | 0.026 | 2686.71 | 0.952 |  | 12.65 | 206.07 | 0.934 |  | 0.05 | 1.23 | 0.951 |
|  | 200 |  | 0.03 | 7738.97 | 0.967 |  | 11.45 | 255.07 | 0.939 |  | 0.03 | 0.90 | 0.985 |
| 0.75 | 100 |  | 0.24 | 1.97x10^12^ | 0.830 |  | 3.01 | 113.05 | 0.833 |  | 0.05 | 2.61 | 0.976 |
|  | 125 |  | 0.24 | 1.06x10^16^ | 0.901 |  | 2.36 | 147.83 | 0.844 |  | 0.02 | 2.90 | 0.923 |
|  | 150 |  | 0.05 | 4295.05 | 0.914 |  | 11.51 | 112.30 | 0.948 |  | 0.04 | 1.22 | 0.960 |
|  | 175 |  | 0.05 | 20983.71 | 0.935 |  | 9.37 | 150.44 | 0.919 |  | 0.04 | 1.28 | 0.963 |
|  | 200 |  | 0.04 | 10676.23 | 0.948 |  | 9.08 | 170.63 | 0.940 |  | 0.04 | 0.73 | 0.962 |
| 1.00 | 100 |  | 0.00 | 7810.77 | 0.900 |  | 0.96 | 92.66 | 0.818 |  | 0.04 | 3.42 | 0.958 |
|  | 125 |  | 0.66 | 5.86x10^33^ | 0.904 |  | 1.51 | 112.71 | 0.743 |  | 0.04 | 2.69 | 0.953 |
|  | 150 |  | 0.12 | 2749208.11 | 0.834 |  | 6.83 | 106.60 | 0.800 |  | 0.05 | 1.81 | 0.910 |
|  | 175 |  | 0.13 | 231740327.1 | 0.905 |  | 9.18 | 114.04 | 0.845 |  | 0.03 | 1.86 | 0.939 |
|  | 200 |  | 0.06 | 52826.26 | 0.908 |  | 6.67 | 142.42 | 0.877 |  | 0.06 | 1.10 | 0.966 |
| 1.25 | 100 |  | 2.20 | 2.59x10^73^ | 0.920 |  | 0.21 | 77.43 | 0.855 |  | 0.03 | 4.08 | 0.970 |
|  | 125 |  | 1.28 | 5.81x10^52^ | 0.935 |  | 0.35 | 95.79 | 0.857 |  | 0.04 | 3.62 | 0.939 |
|  | 150 |  | 0.85 | 1.11x10^42^ | 0.950 |  | 0.77 | 112.34 | 0.680 |  | 0.07 | 2.26 | 0.904 |
|  | 175 |  | 0.24 | 8.74x10^12^ | 0.870 |  | 1.22 | 127.28 | 0.709 |  | 0.05 | 2.11 | 0.934 |
|  | 200 |  | 0.39 | 1.77x10^25^ | 0.905 |  | 0.75 | 151.68 | 0.775 |  | 0.05 | 2.52 | 0.920 |
| 1.50 | 100 |  | 3.83 | 1.45x10^107^ | 0.983 |  | 0.08 | 65.17 | 0.927 |  | 0.02 | 4.34 | 0.945 |
|  | 125 |  | 2.27 | 7.46x10^78^ | 0.905 |  | 0.24 | 80.52 | 0.868 |  | 0.03 | 3.94 | 0.942 |
|  | 150 |  | 1.48 | 2.50x10^59^ | 0.829 |  | 0.99 | 94.02 | 0.738 |  | 0.05 | 3.28 | 0.988 |
|  | 175 |  | 0.49 | 1.04x10^23^ | 0.859 |  | 0.71 | 108.73 | 0.785 |  | 0.02 | 3.56 | 0.907 |
|  | 200 |  | 0.15 | 61566656.72 | 0.876 |  | 3.53 | 110.70 | 0.802 |  | 0.06 | 2.39 | 0.987 |

**
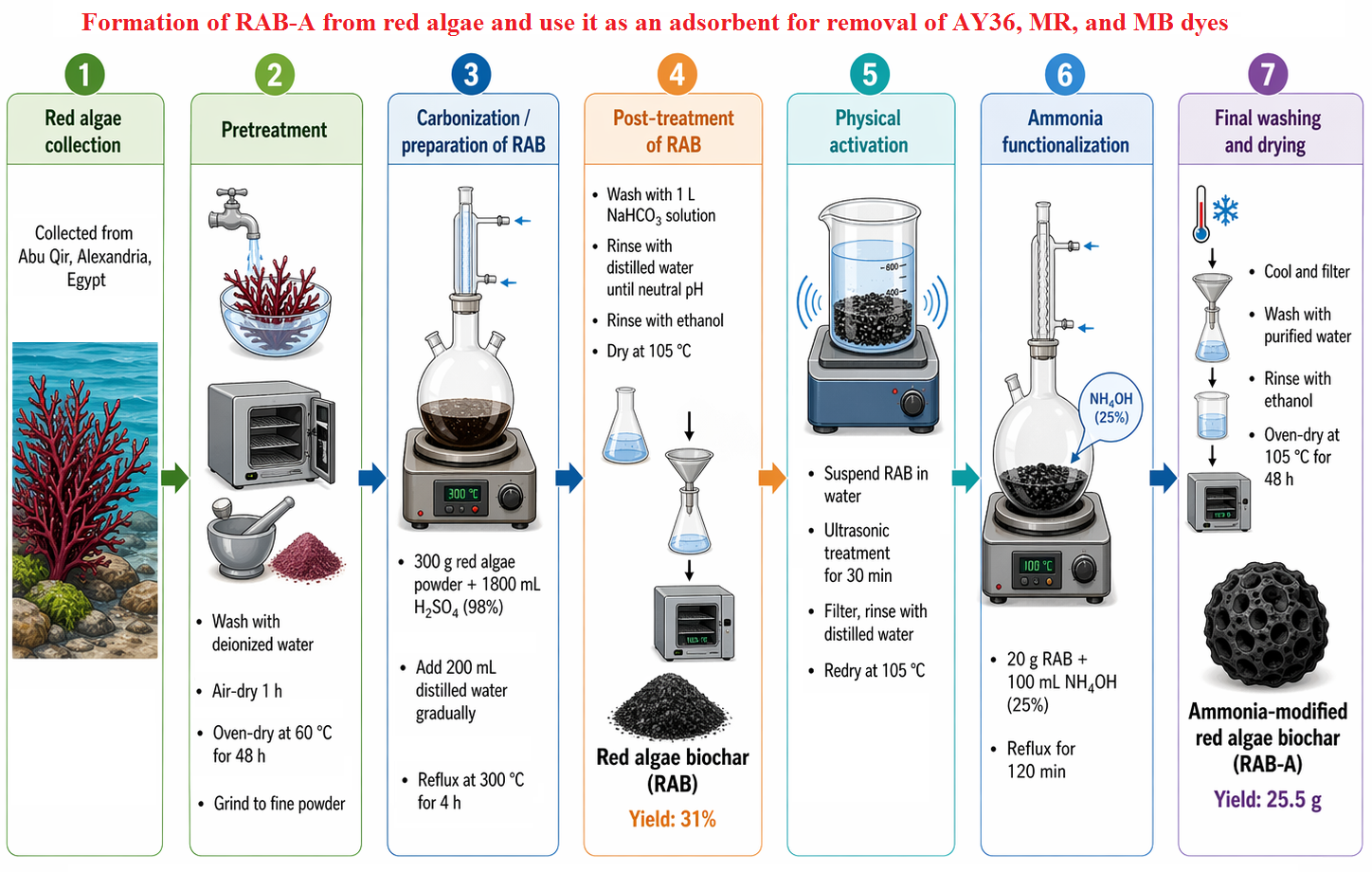
**

**Fig. S1** Schematic illustration of the preparation of ammonia-modified red algae biochar (RAB-A).

**Cost–benefit analysis**

The analysis is techno-economic in nature, with a qualitative/semi-quantitative focus rather than a full financial model. The current system has a favorable cost-benefit profile in terms of adsorbent performance and raw-material sustainability, however some preparation processes may increase manufacturing costs on a bigger scale. On the plus side, RAB-A is generated from red algae, a renewable and environmentally sustainable feedstock, and the final material demonstrated high adsorption capability against three structurally distinct colors. The maximal absorption capabilities reached 222.22 mg/g for AY36, 192.31 mg/g for MR, and 833.33 mg/g for MB. Effective treatment was accomplished with relatively modest adsorbent dosages of 0.5-1.5 g/L. These data indicate that a relatively small mass of the sorbent is capable of removing a significant amount of dye, a key operational advantage in wastewater treatment. Based on Langmuir's reported capacities, the theoretical amount of sorbent required to remove one kilogram of dye is approximately 4.50 kg of RAB-A for AY36, 5.20 kg for MR, and only 1.20 kg for MB, highlighting strong economic potential for removing cationic dyes. Another important advantage is that the sorbent is multifunctional and not specific to any one dye. It has proven effective with both anionic and cationic dyes, with AY36 and MR exhibiting optimal adsorption under acidic conditions, while MB showed optimal adsorption under alkaline conditions. This broad applicability boosts RAB-A's practical utility since it may eliminate the need to prepare separate adsorbents for different kinds of dye pollutants. Furthermore, the ANN results show that process settings can be computationally adjusted, thereby reducing the need for experimental trial and error and improving process control during scale-up. However, the cost of the process is not insignificant. RAB-A preparation needs intense H_2_SO_4_, NaHCO_3_ washing, repeated rinsing with water, ethanol washing, ultrasonic treatment, NH_4_OH functionalization, and many drying/heating processes. In instance, the carbonization technique entails treating 300 g of red algae powder with 1800 mL of 98% H_2_SO_4_, gradually adding water, refluxing at 300 °C for 4 hours, and oven drying at 105 °C. The following ammonia-functionalization step requires 100 mL of 25% NH_4_OH for 20 g RAB, followed by filtration, washing, ethanol rinsing, and oven drying at 105 °C for 48 hours. These chemical and energy inputs are likely to be the most significant cost consequences of the process, particularly if the material is produced on an industrial scale. Furthermore, the optimal pH values increase operating costs because AY36 and MR removal were highest at pH 2, whereas MB removal was maximized at pH 12, implying acid/base usage for wastewater conditioning. From a practical standpoint, the total economic appeal of this activity is therefore determined by the balance between these two factors: high adsorption effectiveness and low adsorbent dosage on the one hand, and chemical-intensive synthesis and pH-adjustment requirements on the other. The strongest economic rationale appears to be for applications that require high-value treatment performance, particularly for MB-contaminated streams with excellent adsorption capability. The technique would become more competitive for real-world industrial implementation if the synthesis route was streamlined, solvent and water consumption were reduced, and adsorbent regeneration/reuse was proved. Thus, the current findings suggest RAB-A as a promising high-performance adsorbent, but a thorough techno-economic evaluation should still include chemical prices, energy consumption, regeneration efficiency, and sludge/disposal costs before large-scale deployment can be completely justified.

## **Table S11. Qualitative/semi-quantitative cost–benefit analysis of RAB-A for dye removal**

| Aspect | Cost implication | Evidence from this study | Benefit implication | Overall assessment |
| --- | --- | --- | --- | --- |
| **Raw material** | **Low** | RAB-A was prepared from **red algae** | **High** – low-cost, sustainable precursor | **Favorable** |
| **Preparation chemicals** | **High** | Synthesis required **98% H_2_SO_4_**, **NaHCO₃**, **ethanol**, and **25% NH₄OH** | **Moderate** – improves surface functionality and adsorption performance | **Mixed** |
| **Energy demand** | **High** | Preparation involved **reflux at 300 °C for 4 h**, drying at **105 °C**, and ultrasonic treatment | **Moderate** – contributes to adsorbent formation and activation | **Cost-sensitive** |
| **Adsorption capacity** | **Low operating cost per unit dye removed** | Langmuir maximum capacities were **222.22 mg/g (AY36)**, **192.31 mg/g (MR)**, and **833.33 mg/g (MB)** | **Very high** – strong adsorption efficiency, especially for MB | **Highly favorable** |
| **Adsorbent dose requirement** | **Low to moderate** | Effective performance observed at **0.5–1.5 g/L**; best results at **1.5 g/L** | **High** – relatively small mass can treat significant dye concentration | **Favorable** |
| **Removal efficiency** | **Low** | At 100 mg/L, maximum removals reached **88.43% (AY36)**, **99.25% (MR)**, and **99.25% (MB)** | **High** – strong treatment efficiency | **Favorable** |
| **pH adjustment requirement** | **Moderate to high** – acid | Optimum adsorption occurred at **pH 2** for AY36 and MR, and **pH 12** for MB | **High** – allows efficient removal under controlled conditions | **Moderate** |
| **Versatility of adsorbent** | **Low** | Same adsorbent removed both **anionic dyes (AY36, MR)** and **cationic dye (MB)** | **High** – one adsorbent can treat different dye classes | **Highly favorable** |
| **Textural/functional advantages** | **Moderate preparation cost** | RAB-A contained oxygenated groups and nitrogen functionalities, with mesoporous structure | **High** – promotes electrostatic attraction, H-bonding, π–π interaction, and pore filling | **Favorable** |
| **Adsorbent requirement per kg dye removed (theoretical)** | **Low to moderate** | Approx. **4.50 kg RAB-A/kg AY36**, **5.20 kg RAB-A/kg MR**, and **1.20 kg RAB-A/kg MB** | **High**, particularly for MB removal | **Favorable** |
| **Overall techno-economic outlook** | **Moderate to high** | High adsorption performance but chemically and energetically intensive synthesis | **High** | **Promising, especially if synthesis and pH-adjustment costs are optimized** |
